# Recovery of mitogenomes from whole genome sequences to infer maternal diversity in 1883 modern *taurine* and *indicine* cattle

**DOI:** 10.1038/s41598-022-09427-y

**Published:** 2022-04-04

**Authors:** Jigme Dorji, Christy J. Vander Jagt, Amanda J. Chamberlain, Benjamin G. Cocks, Iona M. MacLeod, Hans D. Daetwyler

**Affiliations:** 1 School of Applied Systems Biology, La Trobe University, Bundoora, VIC 3083 Australia; 2 Agriculture Victoria, AgriBio, Centre for AgriBioscience, Bundoora, VIC 3083 Australia

**Keywords:** Agricultural genetics, Genotype, Haplotypes, Population genetics

## Abstract

Maternal diversity based on a sub-region of mitochondrial genome or variants were commonly used to understand past demographic events in livestock. Additionally, there is growing evidence of direct association of mitochondrial genetic variants with a range of phenotypes. Therefore, this study used complete bovine mitogenomes from a large sequence database to explore the full spectrum of maternal diversity. Mitogenome diversity was evaluated among 1883 animals representing 156 globally important cattle breeds. Overall, the mitogenomes were diverse: presenting 11 major haplogroups, expanding to 1309 unique haplotypes, with nucleotide diversity 0.011 and haplotype diversity 0.999. A small proportion of African *taurine* (3.5%) and *indicine* (1.3%) haplogroups were found among the European *taurine* breeds and composites. The haplogrouping was largely consistent with the population structure derived from alternate clustering methods (e.g. PCA and hierarchical clustering). Further, we present evidence confirming a new indicine subgroup (I1a, 64 animals) mainly consisting of breeds originating from China and characterised by two private mutations within the I1 haplogroup. The total genetic variation was attributed mainly to within-breed variance (96.9%). The accuracy of the imputation of missing genotypes was high (99.8%) except for the relatively rare heteroplasmic genotypes, suggesting the potential for trait association studies within a breed.

## Introduction

Based on archaeogenetic evidence, modern day cattle originated from at least two distinct wild aurochsen (*Bos primigenius*) following two separate domestication events: one in the Fertile Cresent approximately 10,000 years ago and the second in the Indus Valley some 8000 years ago^1–4^. After domestication, cattle spread to Europe with human migration mainly along the Mediterranean coastline and the Danube River^5,6^ to reach the British Isles (6500 years ago). These cattle populations also expanded to the Iberian Peninsula following the northern coastal region of Africa^5,7^. Similarly, cattle from the Indus Valley spread to China and South-East Asia^8^ and Africa (~ 2500–3500 years ago)^9–11^. The two genetically distinct major cattle sub-species from these two early domestication sites still predominate in modern day cattle as *Bos taurus taurus* and *Bos taurus indicus* along with their widespread crossbreds.

An important part of the molecular evidence for the origin of cattle has been based on mitochondrial DNA (mtDNA) studies. The mitochondrial genome is small (16.34 kb), circular, haploid, non-recombining and maternally inherited^12^. Mitochondrial genome diversity can be described at three levels: nucleotide positions, haplotypes (unique sequences of nucleotides) and haplogroups (higher level of related groups among the haplotypes). Nucleotide diversity describes the average number of nucleotide differences per site in pairwise comparisons among DNA sequences^13^. Haplotype diversity is the probability that two randomly sampled alleles are different^13^. Mitochondrial haplotype clustering^14^ and mitochondrial haplogroups based on a set of known and pre-defined mutations point to plausible maternal origins and evolutionary history. The compiled haplogroup trees and the corresponding mutations were based on 233 cattle previously used for haplogrouping^15–17^ available from GenBank and are publicly available as a resource called DomeTree^18^. The mitochondrial haplogroups represent a group of haplotypes with a similar combination of SNPs arising from the ancestral mtDNA with sequential accumulation of mutations through maternal lineages^19^. That is, haplogroups define distinct maternal lineages in wild and domesticated cattle.

While the mitochondrial genetic diversity of many cattle breeds has been previously characterized^20^, there is increasing interest in the role of mitochondrial diversity on important traits in both humans and livestock animals. In humans, mitochondrial mutations have been associated with several conditions such as LHON (Leber hereditary optic neuropathy), MELAS (mitochondrial myopathy, encephalopathy, lactic acidosis and stroke-like episodes), MIDD (maternally inherited diabetes and deafness) as reviewed in^21^. In livestock, there is no clear evidence of causality, but mitochondrial haplotypes/mutations have been associated with meat quality^22^, litter size^23^, and reproductive capacity^24^ in pigs, as well as increased milk production in cattle^25^. At a cellular level, mitochondrial haplotypes have been shown to influence DNA methylation and gene expression in embryonic stem cells^14^, as well as metabolic traits in porcine and bovine cybrids (cybrids is a cytoplasmic hybrid cell lines containing different cytoplast against uniform nuclear background)^26,27^.

To date, most mitochondrial molecular diversity studies in cattle are primarily evaluated based on the non-coding hypervariable control region (D-loop) or involved limited breeds either within a country or within a region^15,20,28,29^. While the partial or whole D-loop region is informative for population genetics because it is hyper-variable, whole mitochondrial genome sequences are more likely to reflect the full range of mitochondrial genomic diversity^30^. Now that large sequence databases for cattle are available, it is timely to undertake a comprehensive study involving worldwide breeds, countries and continents for a holistic understanding of the mitochondrial genomic landscape in modern cattle. One such database available for cattle is from the 1000 Bull Genomes project^31^.

The nuclear DNA variants from the 1000 Bull Genomes project have been extensively used in genomic analyses, particularly for imputation, genome-wide association and genomic predictions in dairy and beef cattle^32–36^. The variants from autosomal chromosomes have also been used to determine population structure and ancestry of bulls^37^. On the other hand, the 1000 Bull Genomes project mitochondrial sequence variants have not been used in mitochondrial diversity studies. While the imputation of mitochondrial variants for population genetics studies is not recommended, it is clearly of interest to empirically test the accuracy of imputation of mitochondrial variants. Large scale data sets of imputed mitogenomes could contribute in predicting and associating phenotypes to the mitochondrial haplotypes in parallel to the variants from autosomal chromosomes.

The use of mitochondrial variants for mitochondrial diversity from the 1000 Bull Genomes project requires close attention to two key aspects of the data. First, the short-read sequence data are not specific to the mitochondrial genome only and some nuclear mitochondrial sequences (NUMTs) can potentially be wrongly aligned to the mitochondrial genome. This may manifest as heteroplasmy (multiple alleles observed within an animal at a given MT position) but the expectation is that for most tissues, the MT allele reads will be more numerous than NUMT alleles. Thus, a read depth filter could help mitigate this issue. However, due to the low number of mitochondria in sperm cells, the wrongly aligned NUMTs will be harder to be distinguished from true mitochondrial reads due to more even read depth at heteroplasmic sites. This necessitates strict quality control and filters to minimize the impact of NUMTs on the analysis. It should also be noted that true heteroplasmy in MT genomes does exist due to multiple mitogenome copies sometimes carrying different mutations (reviewed in^38^). Further, the format in which the 1000 Bull Genomes Project data is presented (VCF) is not a standard input format for most of the available mtDNA analysis tools. The format conversion must consider the attributes specific to mtDNA (haploid, missing bases, heteroplasmy, and INDELs). Currently, there are tools (e.g. BCFtools)^39^ to derive a consensus sequence from VCF format to the routinely used fasta format, but for mitochondrial variants these tools lack description, particularly on the handling of heteroplasmy. Furthermore, the allocation of the allelic base call at heteroplasmic positions in haploid genotypes needs careful consideration because heteroplasmy is not generally considered in diversity analyses.

We used the mtDNA variants from the 1000 Bull Genomes Project to:

- develop an approach to pre-process and filter the MT sequence data from the 1000 Bull Genomes project to remove samples that may be contaminated with NUMTs,
- evaluate cattle mitochondrial diversity, haplotypes, and haplogroups across and within cattle breeds,
- compare unsupervised clustering techniques to conventional mitochondrial grouping tools using whole mitogenomes, and
- investigate the accuracy of imputation of sporadic missing mitochondrial variants for inclusion in haplogroup assignment.

## Materials and methods

### Sequence data and filtering

Our study utilised whole mitochondrial genomes dataset from Run 8 of the 1000 Bull Genomes Project^31^ (henceforth referred to as “Run 8”). Run 8 included 4931 animals representing over 200 *taurine* and *indicine* breeds and their crosses. Prior to our study, as part of the standardised processing for Run 8, all mitochondrial sequence data was aligned to the latest Bovine Reference Genome, ARS-UCD1.2_Btau5.0.1Y.fa. This reference combines ARS_UCD1.2^40^ with the Y chromosome assembly from Btau5.0.1^41^ because the ARS-UCD-1.2 animal was female and includes the mitochondrial (M) genome version from the ARS-UCD-1.2 assembly. The sequence processing for Run 8 from raw fastq sequence files through to variant calling and creation of the VCF files (that we used in this study) is described here (Supplementary Methods 1). The average read coverage per animal across the mitochondrial genome was 12.34. The raw vcf file included over 6000 mtDNA variants: 5420 SNPs and 836 INDELs. Heteroplasmy (due to a mixture of two or more mitochondrial genomes or NUMT interference) was observed at almost all variant positions (5119 out 5943). The mean number of heteroplasmy per SNP and per animal was 253.0 and 302.2, respectively. The mean number of missing genotypes per SNP was 464.0, and the mean number of missing genotypes per animal was 160.0.

In order to obtain a high quality and reliable dataset for the analysis, we applied quality filters at both site and individual animal levels. The site quality control thresholds used were similar to those applied in nuclear sequences of the 1000 Bull Genomes Project (Supplementary Methods 1). We applied thresholds per site of: minimum phred-score quality of 30 (Q30), minimum mapping quality of 30 (MQ30), minimum minor allele count of 2 (AC2) and maximum read depth (DP) of mean + 3 SDs using VCFtools and BCFtools^39,42^. This preliminary filtered dataset consisted of 3394 polymorphic sites, including heteroplasmic sites and INDELs. We then filtered out INDELs and variant sites with missing genotypes because these are not efficiently handled in conventional mtDNA analysis tools and are generally discarded from the analysis. Thus, no microsatellites were included. Further, we imposed an individual animal filter based on average read depth coverage and heteroplasmic sites. The animals with low average read coverage (DP < 10) across all remaining sites were removed, such that 2176 remained out of 4931 animals. Further, to develop filters to remove animals that may have excessive and/or questionable heteroplasmy due to contamination from NUMTs, animals were evaluated in two groups as:

- males whose DNA samples were either from semen or from unknown tissue (Semen group), and
- females and males with DNA sampled from known tissues other than semen (Non-semen group).

The distribution of the number of heteroplasmic sites per individual between groups was compared. The heteroplasmic site distribution in the non-semen group approached a maximum of 150 heteroplasmic sites per individual compared to a maximum of over 700 heteroplasmic sites per individual in the semen group (Fig. S2). We therefore applied a maximum threshold of 150 heteroplasmic sites per animal in the semen group, removing 293 animals to leave 1883 animals in the data set for our analyses. Details of the breed groups available in this final study set are shown in Table S3.

Further, the allelic ratios of the major to minor alleles at the heteroplasmic positions in the semen group showed an increase following the application of this filter (Fig. S3) so that the major allele count was nearly twice that of the minor alleles.

### Data processing

The existing mitochondrial DNA analysis tools either require a continuous stretch of mitochondrial DNA sequence from a specific region (D-loop, COX2, CYTB etc.) or a whole mitochondrial genome in prescribed formats. We adopted a genotype-based allele assignment approach for the conversion to a homoplasmic variant sequence. Homoplasmic variants (0/0, 1/1, 2/2 etc.) were directly assigned the corresponding alleles, while the heteroplasmic sites (0/1, 0/2, 0/3, 1/3 etc.) were assigned a homoplasmic status for the most abundant allele based on read depth. In other words, the allele (reference—REF or alternative—ALT) with a higher read depth was chosen as the most representative base for a sequence at heteroplasmic positions. In cases where the allele read depth of REF and ALT alleles were equal, the ALT allele was chosen as the base for the position in the sequence. It was assumed that this strategy would be more informative of the existing allelic diversity and would help avoid reference bias. In addition, we also generated a complete genome length sequence (16,340 bp in fasta format) using bases for variant positions and inserting "N" (missing base) in non-variant positions because this full mitochondrial genome length sequence format was required to predict the haplogroups using traditional tools (maternal origin and lineages).

### Analysis

#### Mitochondrial DNA polymorphism, diversity and haplotypes

The variant sequences (derived from VCF) were used for the description of the overall DNA polymorphism for all animals and the evaluation of nucleotide and haplotype diversity using the DnaSP program^43^ for 15 selected breeds (with N ≥ 20 animals). We conducted an analysis of molecular variance (AMOVA) using Arlequin 3.5^44^ to detect population differentiation: an approach based on a matrix of squared differences among all pairs of haplotypes within selected breeds. We used the maximum likelihood tree implemented in the MEGA X program^45^ to derive a phylogenetic relationship among the breeds. MEGA X conducts a statistical analysis of molecular evolution across the maternal lineages to construct the phylogenetic tree. The haplotype network within breed was constructed by employing the median joining tree approach in the PopART program^46^. This program enables construction and visualization the relationship between individual haplotypes but the network does not explicitly reconstruct the evolutionary history.

#### Mitochondrial haplogroups

Mitochondrial haplogroups were predicted using the MitoToolPy program^18^ using the whole mitochondrial genome sequence (fasta) prepared as described above. At the time of development (2015) this tool captured a list of variant positions across the mitogenome that published studies had used to define distinct bovine haplogroups. The corresponding sequences identified to a haplogroup which were available in the public repositories are used as the reference. The aim of the tool was to automate assigning of haplogroups and to standardise the haplogroup definitions regardless of whether a study used a specific region or the entire mitogenome. The tool aligns the query sequences to the bovine reference sequence V00654 (hereafter referred to as BRS) which was generated using a shotgun DNA sequencing strategy^12^. It then compares the alleles at all variant sites that overlap the predetermined list to assign individuals to specific haplogroups. In total, MitoToolPy covers all previously defined haplogroups; I1, I2, T1 to T7, P1, P2, Q1, Q2, R1 and R2. The tool output also provides lists of missing variants in the query sequence for an assigned haplogroup and a list of new variants not in an assigned haplogroup, but present in query sequence as private variants. The private variant output from the tool provides the opportunity to infer a new subgroup within a haplogroup and to annotate the variants specific to a haplogroup and breed.

Additionally, because of the extensive use of D-loop sequences in determining mitochondrial diversity and haplogroups in the past, mitochondrial variant sequences from only the D-loop region were also used to predict the mitochondrial haplogroups in MitoToolPy as a comparison. The outputs from the MitotoolPy (private and missing variants) had slightly altered nucleotide positions due to being aligned to an older reference genome (BRS) incorporated in the software. To enable the annotation of variants to the latest reference (ARS-UCD1.2_Btau5.0.1Y_M.fa, hereafter known as ARS-UCD1.2_M), the haplogrouping variants in the MitoToolPy were lifted over (positions and bases) to ARS-UCD1.2_M, and the reference genome for alignment within the tool was changed to the ARS-UCD1.2_M.fa. The two reference genomes differed in their length (BRS 16,338 bp, ARS-UCD1.2_M 16,340 bp) resulting from two deletions in the former as well as having nucleotide base differences at 12 positions (Table 1). Briefly, all haplogroup determining variants in MitoToolPy after 222 bp were incremented by + 1 up to 588 bp and positions after 588 bp (BRS) by + 2 to correct for the two deletions. Further, bases were changed as appropriate, i.e. five variants among the haplogroup determining variants were the same base as ARS-UCD1.2_M and thus removed as they were no longer variant when lifted over to the ARS-UCD1.2_M (Table S1). The position changes resulting from the manual liftover were confirmed by aligning 218 complete mitochondrial DNA sequences (previously used to derive variants for haplogroups in MitoToolPy available from NCBI under the same accession number) to the ARS-UCD1.2_M and these conformed to Table S1 and showed additional variants (Table S2).

**Table 1:** Equivalent positions and reference (Ref) alleles differing between ARS-UCD1.2 (ARS) and Bovine reference sequence (BRS) relative to ARS-UCD1.2 and indicating whether variant positions belong to the pre-defined haplogroup variant set as defined in cattleTree_whole.txt file of MitotoolPy.

ARS position & Ref. allele	BRS position & Ref. allele	Haplogroup variant in MitoToolPy	Comments
222 C	– (deletion)	No	Deletion at position 222
364 G	363 C	No	
589 C	– (deletion)	No	Deletion at position 589
2538 A	2536 C	Yes (2536 A)	ARS and HG_variants have same base
3345 G	3343 C	No	
3387 C	3385 T	No	
3541 A	3539 G	No	
4321 C	4319 T	No	
8190 C	8188 T	Yes (8188 C)	ARS and HG_variants have same base
8712 T	8710 C	No	
9684 C	9682 G	Yes (9682 C)	ARS and HG_variants have same base
12,167 C	12,165 T	Yes (12,165 C)	ARS and HG_variants have same base
13,312 C	13,310 A	Yes (13,310 C)	ARS and HG_variants have same base
15,637 T	15,635 C	No	

### Private variants and annotation

We investigated private variants specific to certain individuals within a haplogroup and within individuals within a breed to investigate whether they may be biologically meaningful. The private variants specific to a particular group within a haplogroup/breed in this study were annotated using SNPeff^47^. The importance of the coding variants was predicted by SNPeff as being either high (e.g. stop gained), moderate (missense variants) or low (synonymous variant) and non-coding variants were annotated as modifier (e.g. upstream/downstream variants).

### Unsupervised clustering

The overall mitochondrial population structure was also investigated through three unsupervised clustering approaches. First, clustering based on principal components was derived from a genomic relationship matrix (GRM) generated between all animal pairs from all filtered polymorphic variants. Fasta files were converted to .bed format using Plink ver1.9^48^ and these genotypes were used to generate a haploid GRM (***make-grm-xchr*** option in GCTA^49^) to use as input for a principal component analysis (PCA) also completed with GCTA. Principal components (PCs) 1, 2 and 3 were plotted using scatterplot3d^50^, and the clustering was interactively visualised using the *rgl* package in R^51^. Second, we determined the individual ancestry and population structure of the animals using Admixture^52^. Admixture software employs maximum likelihood to estimate the proportion of each individual’s alleles (using SNP genotypes) that are ancestral to one of *k* populations. Clearly then, from our filtered set of 3069 SNP on this haploid non-recombining mitogenome, we expected that Admixture would only be able to separate broad population clusters such as haplogroups, where there might be an adequate number of alleles to enable differentiation. The pruning of variants for strong LD in diploid genotype data is optional for Admixture and was not undertaken here (within haplogroups many variants are in strong to perfect linkage disequilibrium (LD) because there is no recombination). The estimate of *k,* the expected number of population (sub)groups, must be provided in advance to run Admixture. This was determined using the Admixture cross-validation errors approach, and also an a priori population structure was implemented with k ranging from 2 to 6. The third approach was hierarchical clustering implemented in the R package dendextend^53^ using a matrix of nucleotide differences between each pair of sequences (calculated using an in-house python script). The hierarchical clusters were implemented at the highest (2 groups) as well as the lowest levels (0 nucleotide difference). To check for the concordance between these three unsupervised clustering methods, the resulting clusters/groups were annotated according to the individual's predicted haplogroups from MitoToolPy.

### Imputation of missing genotypes and haplogrouping

The accuracy of imputation of sporadic missing mitochondrial genotypes and the effect of this imputation on haplogroup assignment were investigated. The empirical accuracy of imputation was tested using the filtered sequence dataset that had no missing genotypes. The 1,883 individuals in the filtered data were split into two random groups consisting of 333 (I) and 1,550 (II) animals. A random 10% of the genotypes of individuals in Group I were masked (set to missing) at random sites and then imputed using Beagle 4.0^54^ following the *gt* and *ref* options and providing Group I (as *gt*) and Group II (as *ref*) accordingly. The Beagle software phases genotypes and then imputes the ungenotyped markers or missing markers. Beagle will impute bi- and multi-allelic variants. The accuracy of imputation in Group I was evaluated as the proportion of imputed genotypes in agreement with original genotypes at the masked and unmasked positions separately. The Beagle estimate of alternate allele dose (DS) and genotype probabilities (GP) were used to define the most likely base call at heteroplasmic positions. For example, for an imputed heteroplasmic genotype with a reference and alternate allele of 0|1, 0|2, or 0|3 etc. (where 1, 2 & 3 represent alternate alleles for multi-allelic sites), if the DS is < 1, a Ref allele is assigned while DS = 1 is assigned the Alt allele. In rare cases where heteroplasmy was imputed as two alternate alleles (e.g. GT:DS:GP 1|2:2:0,0,0,0,1,0) where 1 is ALT1, 2 is ALT2 and DS = 2 (which presents equal probabilities for both alternate alleles), the base was then set to missing and when DS is < 2, the more frequent allele (summed across genotype probabilities) was assigned as the base for this position. These variant sequences with imputed sporadic missing genotypes were then reconstituted to a full genome sequence in fasta format by adding Ns at other non-variant positions and then used for re-predicting the haplogroups. The extent of agreement between an individual's haplogroup using the real and partially imputed genotypes was examined. The mean accuracies of imputation and the predicted haplogroup were calculated from 50 repeats of this cross-validation (i.e. resampling Group I and Group II animals and following the above steps). The empirical accuracy of imputation was assessed as the concordance between real and imputed genotypes.

## Results

### General description of variants

The raw variant call dataset was filtered to have high quality SNP genotypes, and both SNP and animals with missing genotypes were removed. This not only reduced the overall number of animals and sites substantially, but also reduced the number of heteroplasmic genotypes, improved the average read depth and retained higher quality sites (Table 2, Fig. S1). However, when we compared the levels of heteroplasmy per individual separately in the Semen and Non-semen tissue groups, heteroplasmy was much higher in the Semen derived samples.

**Table 2:** Summary of the parameters of raw and filtered variant datasets before and after removing of sites with missing data (Site) and removing both sites and animals with missing data (Site & Ani).

Parameters	Raw dataset (unfiltered)	Dataset filtered by	
		Site	Site and Ani
No. of Animals (Ani) in dataset	4931	4931	1883
Total No. of POS in dataset	5903	3394	3069
Total No. of POS with at least one Het_GT animal	5201	3394	1227
Mean No. of Ani with Het_GT per POS (med)	253 (5)	388 (12.5)	2 (0)
No. of Ani with at least one Het_GT	3934	3717	712
Mean No. of POS with Het_GT per Ani (med)	302.2 (278)	266 (245)	3.5 (0)
No. of POS_Missing GT	5903	3394	0
Mean No. of Ani with Missing GT per POS (med)	420 (409)	232.3 (175)	0
No. of Ani with Missing GT	3299	2748	0
Mean No. of POS_Missing GT across all Ani (med)	251.3 (7)	159.9 (3)	0
Mean read depth per POS (across all Ani) (med)	284.5 (299.8)	287 (299.9)	699 (723)
Mean read depth per Ani (across all POS) (med)	284 (18.9)	287 (18.9)	699 (597)

*Ani* animal, *POS* nucleotide position, *GT* genotype, *Het_GT* heteroplasmic genotype, *med* median.

We therefore imposed a strong filter based on the maximum number of heteroplasmic sites per individual, to result in a similar distribution of heteroplasmy and allelic ratios in both the Semen and Non-semen groups (Figs. S2, Fig. S3). Overall, in the final set of 1,883 individuals, the per site heteroplasmy count was considerably reduced and there was a slight improvement in the average read depth coverage in the final dataset (Fig. S4).

### Mitochondrial haplogroups, population structure and admixture

#### Haplogroups using MitoToolPy

The haplogroup membership for each of the 1883 animals in the filtered set was predicted in MitoToolPy using the ARS-UCD1.2_M reference, and the lifted over variants that MitoToolPy uses to define haplogroups (Table S1). MitoToolPy detected 11 major pre-defined haplogroups (I1, I2, T1, T2, T3, T4, T5, T6, P, Q1, Q2) based on variants from the whole genome sequences (16,340 bp). Overall, T3 was the predominant haplogroup (1502 animals) with about 15 subgroups within T3. While the dominant subgroup was the original T3 (N = 752), the next most dominant subgroup was T3r (N = 547) (Fig. S5). In most cases, the predicted haplogroup of each animal was as would be expected based on the breed and sub-species (Table S3). Only a single occurrence of P2 and T4 were observed (a Korean Hanwoo and a Wagyu breed animal respectively) and seven T6 in Angus origin animals. All the African cattle breeds (Ankole, Afrikander, Ndama, Benishangul, Goffa, Kenana, Muturu) were classified as the T1 haplogroup that is fixed in African *taurine* breeds^18^. Generally, the *indicine* cattle breeds belonged to major haplogroup I and modern *taurine* cattle to haplogroup T, although there were some exceptions. As expected, the composite breeds mostly sourced from Australia were unpredictable. Notably, the haplogroups of Brahman cattle (N = 18) were mostly T1 (N = 12), T3 (N = 5) and one indicus (I). In some animals of European breed origin and their composites (N = 1302), the integration of T1 (3.5%), I1 and I2 (1.3%) haplogroups was also observed (Tables 3 and 4). For example, several Holstein (N = 5) and Jersey cattle (N = 4) from Australia were of T1 origin. This was further confirmed by checking the original haploid genotypes for heteroplasmy across the haplogroup determining positions. For Jersey belonging to T1, the haplogroup determining positions were all homoplasmic (except 1 position in one animal) (Table S4). Altogether, the T1 haplogroup was observed in about 13 European *taurine* breeds and composites. Within Australian sourced cattle, T1 had considerable influence on Holstein, Jersey and composite breeds (36 animals). Similarly, the I Haplogroup was present in Holstein animals from China (N = 5/12), Herefords from New Zealand (N = 3/4) and, as expected, in composite taurus x indicus breeds from Australia 4/12 (Table 4).

**Table 3:** Prevalence of T1 (African *taurine*) haplogroup in non-African cattle breeds and composites.

Origin of sample	Breed	Sub-species	n/N	Sex
Australia	Angus Lowline	*taurus*	2/2	2F
	Beefmaster	*taurus *× *indicus*	1/2	1M
	Brahman	*indicus *× *taurus*	12/18	1F, 10M, 1U
	Dexter	*taurus*	1/2	1F
	Holstein	*taurus*	5/5	4F, 1M
	Jersey	*taurus*	4/8	4M
	Senepol	*European taurus *× *African taurus*	5/12	5U
	Composite		6/13	6M
Germany	Holstein Red	*taurus*	1/3	1M
France	Blonde d’Aquitaine	*taurus*	2/16	1F, 1M
	Brown Swiss	*taurus*	1/1	1M
Korea	Hanwoo	*taurus*	2/21	2U
Unknown	Holstein	*taurus*	1/67	1F
	Romagnola	*taurus*	2/10	1M, 1U
	San Martinero	*taurus*	1/2	1M
	Limonero	*taurus*	1/9	1U

F female, M male, U unknown, n no. of animals showing T1 haplogroup, N no. of animals in a breed sampled from the specified country.

**Table 4:** Prevalence of *indicine* haplogroup (I) in European *taurine* breeds and composites.

Origin of sample	Breeds	n/N	Sex	Haplogroup
Australia	Composite	4/12	M	I1
	Brahman	1/18	M	I1
	Belted Galloway	1/2	F	I1
China	Holstein	5/12	F	I1
New Zealand	Hereford	1/4	M	I2
	Hereford	2/4	M	I1
Unknown	Shorthorn	1	F	I1

F female, M male, U unknown, n no. of animals showing I haplogroup, N no. of animals in a breed sampled in a country.

In the past, sequences from D-loop region (910 bp long) have been extensively used in the prediction of haplogroups^1,18^. However, using our filtered D-loop genotype data, MitoTool.py could not differentiate between the two major I and T haplogroups likely because some variants used in previous studies were filtered out of our variant set. In our dataset prior to any filtering, there were 206 D-loop variants compared to 153 D-loop variants in the pre-defined set that MitotoolPy uses for prediction of haplogroups but only 87 variants overlapped. Further, in our filtered set, only 60 D-loop variants overlapped with the 153 MitoToolPy D-loop variants, suggesting that this was the main contributing factor resulting in poor resolution of haplogroups using only the D-loop variants. On the other hand, using our filtered set of sequence variants from the non-D-loop region, MitoToolPy could distinguish the major haplogroups (I, T, P and Q) but did not resolve haplogroup sub-levels. For example, the incidence of unresolved haplogroups was more than 60% of the animals between T1 and T3 (1280), and T3 and T4 (N 15). This indicates that the D-loop variants in our set played a key role in defining the sub-haplogroups when used together with the non-D-loop. This is not unexpected because the higher mutation rate in the D-loop region is more likely to resolve the sub-haplogroup levels (i.e. more recently diverged groups).

### Principal component analysis

**Figure 1:**
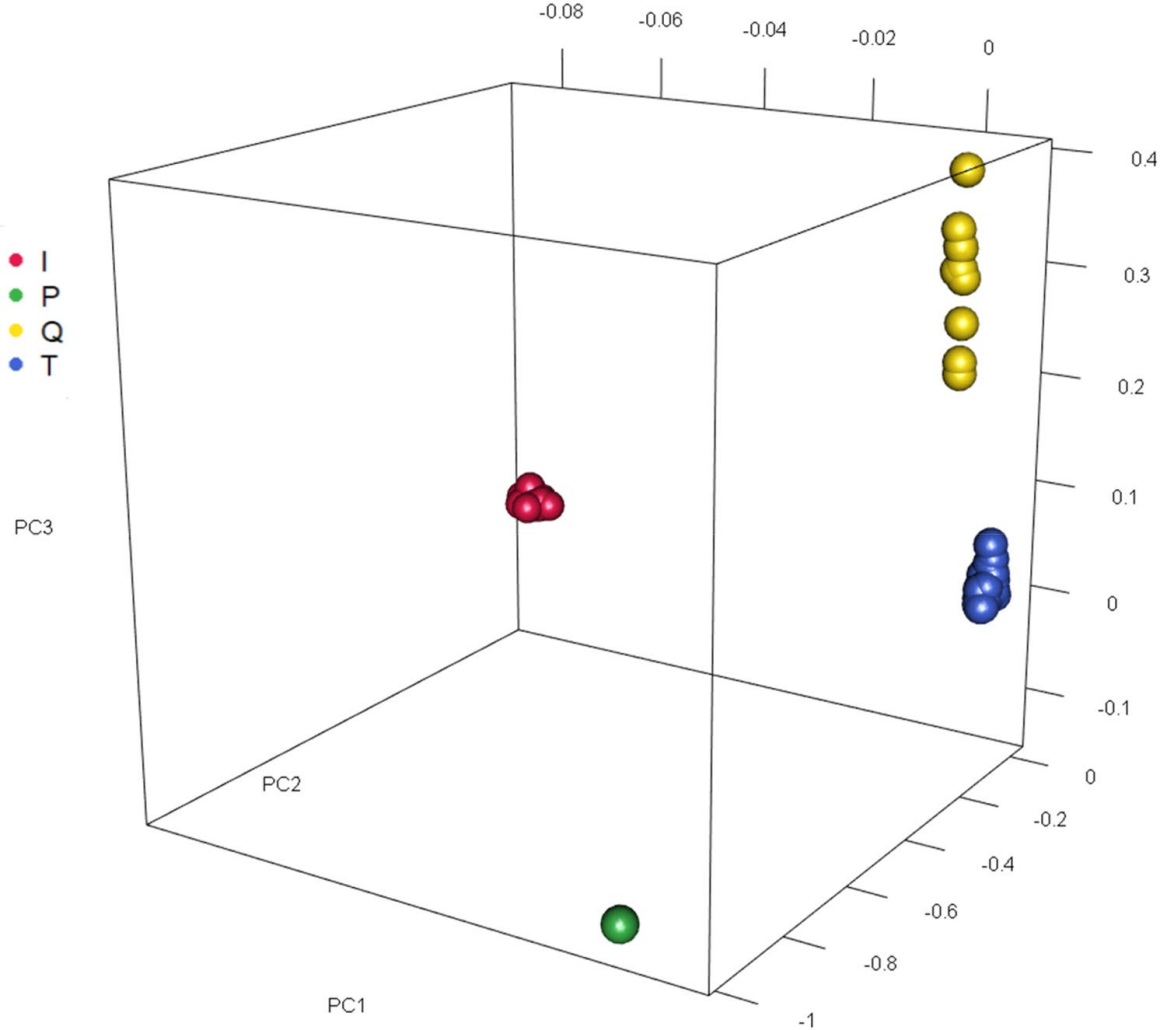
Principal components (PC1, 2, and 3) plot based on mitochondrial genomic relationship matrix showing the grouping of I, P, Q and T major haplogroups.

The PCA of the GRM derived from all filtered mitochondrial variants (whole sequence) revealed distinct clusters that corresponded to the I, T and Q major haplogroups after annotation with MitoToolPy results (Fig. 1). However, sub-clustering within the major haplogroups T and T3 was not entirely resolved, despite the tendency to marginally separate T1 and T2’s (Fig. S6a), as well as T3 and T3r (Fig. S6b).

A GRM of only D-loop variants was also used for PCA and revealed the same two major clusters (T and I, Fig. S7a). Within the I cluster, sub-clusters of I1 and I2 were separated to some extent while T1 and T3 did not separate clearly. Similarly, the variants from the non-D-loop region could segregate T and I haplogroups into separate clusters but did not resolve further into sub-clusters of haplogroups (Fig. S7b).

### Population structure using Admixture

**Figure 2:**
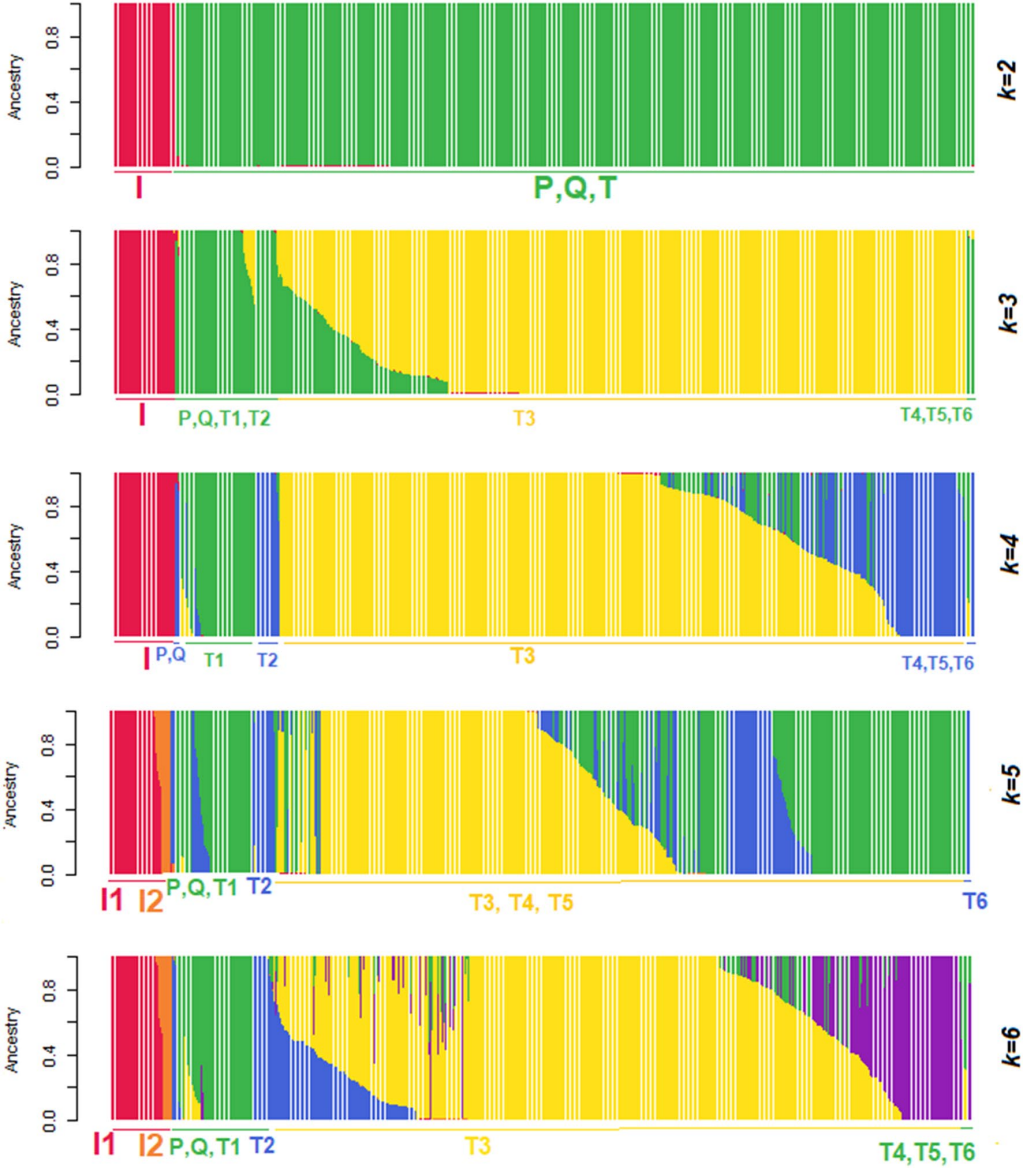
Population structure of cattle mitochondrial sequence variants using Admixture for a pre-defined number of populations (*k*) ranging from 2 to 6. Population structure annotated with individual animal haplogroups (I1, I2, P, Q, T1, T2, T3, T4, T5, T6) determined from MitoToolPy.

The population structure based on all mitochondrial sequence variants was determined using Admixture^52^, where each animal is assigned a proportional membership of a predetermined number of *k* population groups (e.g. sub-species, breeds). Depending on the *k* value used (2–6), the major haplogroups were progressively split (Fig. 2). Admixture estimated the optimal a priori k value to define population groups (based on the changes in cross-validation errors) as four (*k* = 4) (Fig. S8). When annotated with the predicted MitoToolPy haplogroups, the population structure with *k* = 3 showed I separating from two further subpopulations within the T haplogroup. Further sub-groups were apparent at higher *k* values and these corresponded to sub-haplogroups within T.

### Hierarchical clustering

**Figure 3:**
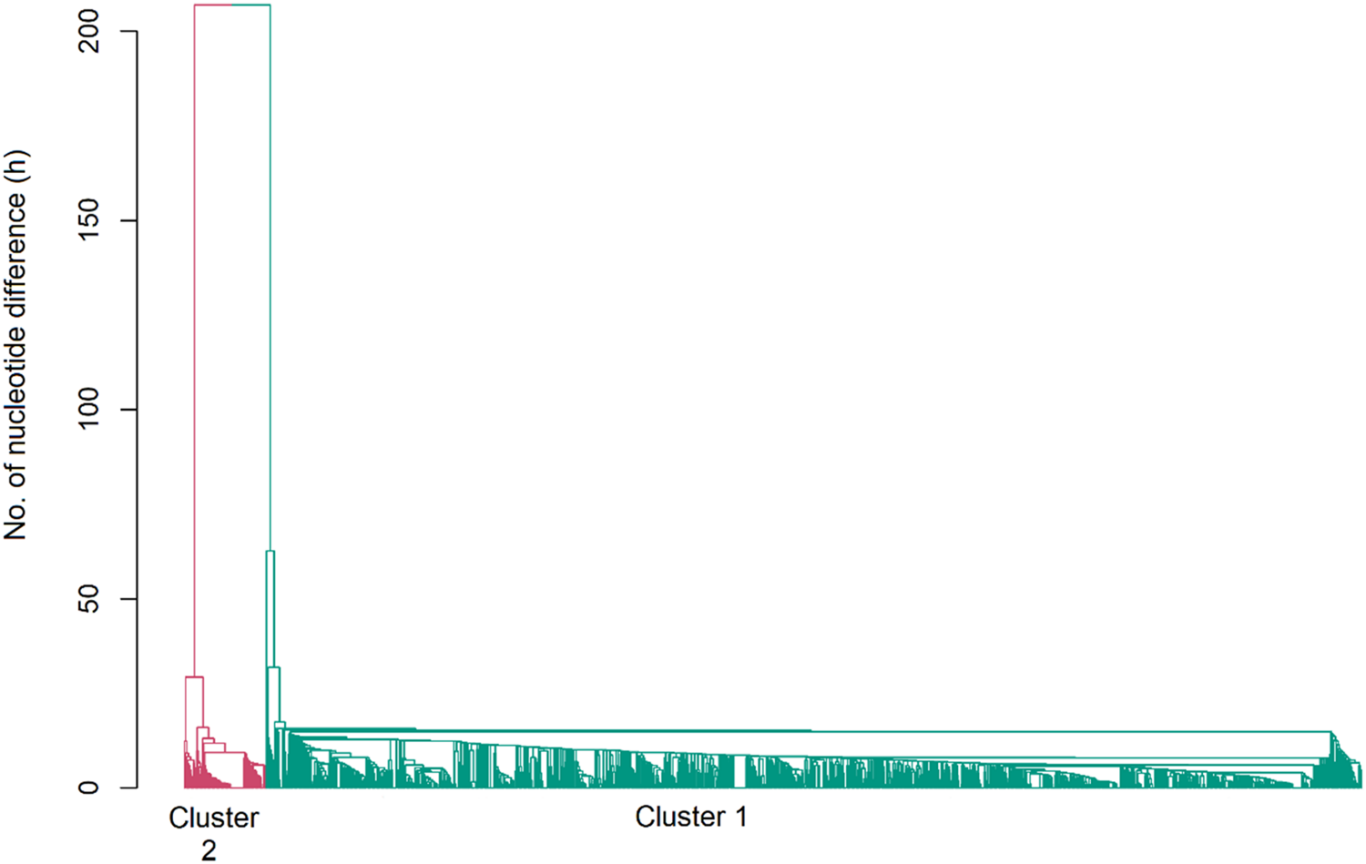
Hierarchical clustering of animals based on the number of nucleotide differences between the pair of mitochondrial sequences. Cluster 1 and 2 corresponded to *taurus* and *indicus* cattle, respectively.

The nucleotide differences between each pair of whole mtDNA variant sequences was calculated using an in-house script. The mean nucleotide difference across all pair combinations was 36 but ranged from 0 to 224. Hierarchical clustering, based on the nucleotide differences matrix between individuals, again presented two broad and distinct clusters (Fig. 3: Cluster 1 and 2). The individuals in Cluster 1 and 2 were from the major haplogroups T and I, respectively and Cluster 1 also included animals belonging to the P and Q haplogroups.

### Private variants

Private variants are additional variants present in a query mitochondrial sequence but not in the list of haplogroup determining variants. They are of interest because they can provide insights into plausible subgroups that have not been previously catalogued within the pre-defined haplogroups. We therefore examined the distribution of these private variants (output from MitoToolPy) within a haplogroup and/or breed(s). Some of the private variants were specific to a group of animals within a haplogroup (Table S5). For the most part, private variants were transition mutations from the reference allele. Four breeds had members of a sub-haplogroup that showed a specific set of private variants (Table 5). Almost 50% of private variants (N = 43) were specific to particular haplogroups, annotated either as missense (50%), upstream/downstream (30%) or synonymous (16%) gene variants (Table S5). Several haplogroups were found to possess specific private variants: 2 SNPs in I1, 1 SNP in I2, 5 SNPs in T1, 2 SNPs in T1b1b1, 1 SNP in T1c, 1 SNP in T2 and 1 SNP in T3. Interestingly, a number of the private variants were annotated as missense, and it is therefore possible that these mutations could have downstream effects on phenotypes.

**Table 5:** Breeds found to have specific private (one breed only) mitogenome variants with annotation of the variants (position, type and genes affected) and number of animals sharing the private variants within each breed.

Breed	Haplo-group	Source of sample	n/N^a^	Annotation: variant position (bp), type, gene
NDama	T1	Benin, Guinea	7/12	2579, NCTE, rRNA
				4714, Missense, ND2
				6882, Missense, COX1
				10,435, Missense, ND4L
Holstein	T3	Switzerland, Canada	6/111	7948, Missense, COX2
	T3d1	United Kingdom	5/7	9807, NCTE, tRNA
				13,277, Missense, ND5
Hereford miniature	T3	Australia	2/2	5603, Synonymous, ND2
Senepol	T1	Australia	3/5	6388, Synonymous, COX1

^a^N = total number of animals in a breed in the sub-haplogroup; n = number of animals with private variants in a breed within the haplogroup; NCTE = non-coding transcript exon.

**Figure 4:**
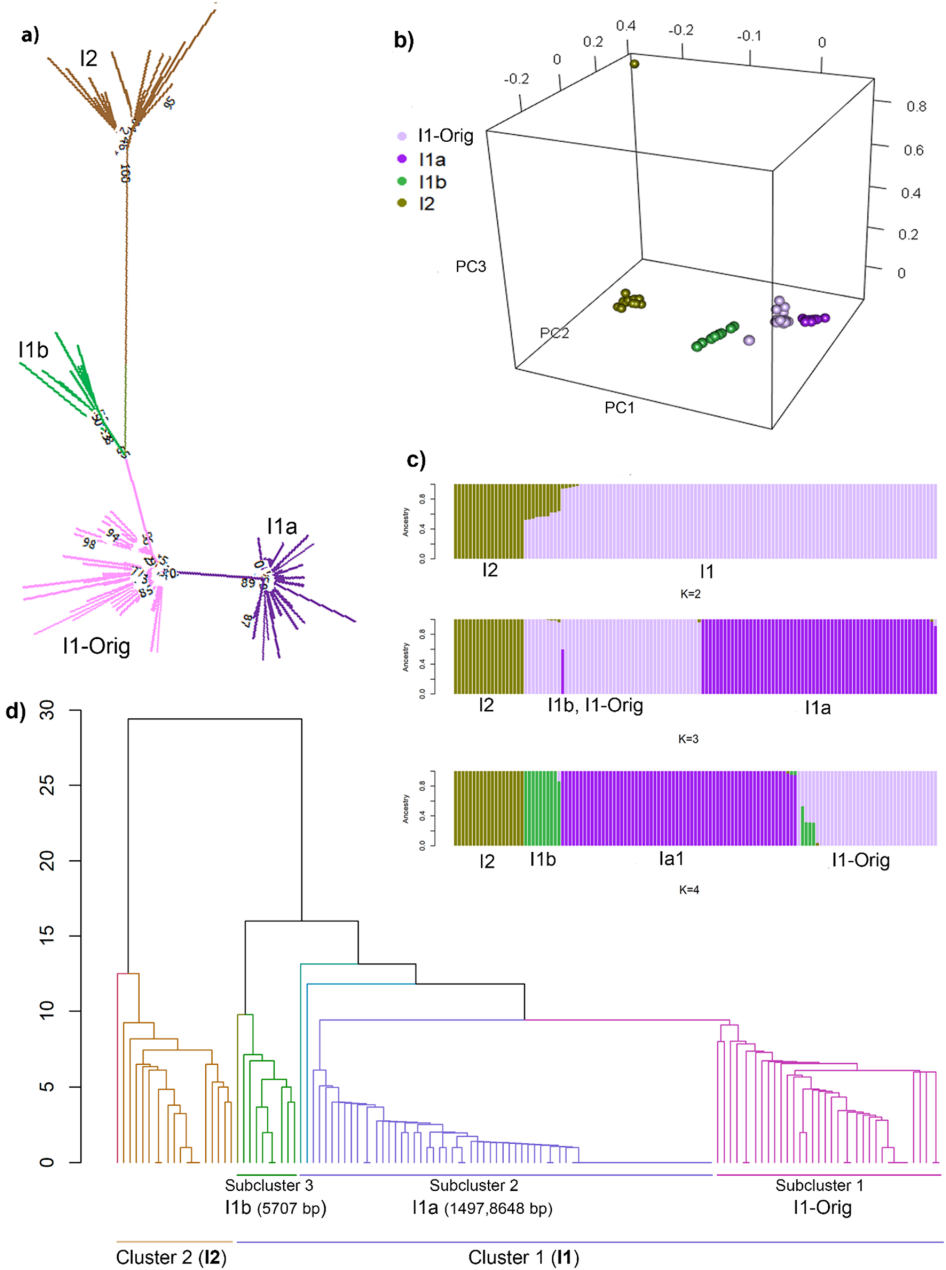
Subgrouping animals under I haplogroup into I2, I1 and subgroups within I1 (I1-Orig, I1a and I1b) using conventional Maximum Likelihood method (**a**) and alternate clustering techniques: principal component analysis (**b**), Admixture software (**c**) and hierarchical clustering based on the number of nucleotide difference between the sequences of pair of animals (**d**). *Base pair position of private variants relative to ARS-UCD1.2_M. I1-Orig is group of animals under previous I1 haplogroup not assigned to either I1a or I1b (i.e., remaining animals in I1 Cluster1).

A maximum-likelihood tree was constructed for whole mitogenomes of only the animals belonging to I haplogroup using MEGA X. This analysis showed four distinct clusters, one cluster corresponded to the I2 haplogroup and the three other clusters were annotated to I1 haplogroup (Fig. 4a). The subclusters within I1 haplogroup were labelled as I1a, I1b and I1-Orig. The cluster I1a consisted of a group of 64 animals which were characterised by two group specific (private) variants (1497 bp and 6848 bp). The cluster I1b contained a group of 10 animals with one group specific variant (5707 bp) (Table 6).

**Table 6:** Breed annotation and the number of animals within subclusters of the indicus (I) cluster based on alternate clustering techniques.

Cluster I2 (N = 19) (p/q)^a^	Cluster I1b (N = 10) (p/q)	Cluster I1a (N = 64) (p/q)	Cluster *I1-Orig (N = 38) (p/q)
Achai (1/4)	Bhagnari (1/4)	Bohai Black (2/5)	Achai (1/4)
Bhagnari (1/4)	Cholistani (2/5)	Buryat (2/21)	Bhagnari (2/4)
Cholistani (1/5)	Dajal (2/4)	Chaidamu Yellow (2/5)	Brahman (1/29)
Dhanni (2/5)	Dhanni (1/5)	Dabieshan (2/3)	Cholistani (2/5)
Dianzhong (1/5)	Gabrialli (1/5)	Dianzhong (1/5)	Dajal (2/4)
Gabrialli (1/5)	Hariana (1/1)	Guangfeng (3/4)	Dhanni (2/5)
Gir (1/1)		Jian (3/3)	Dianzhong (1/5)
Kangayam (1/1)	Composite (2/13)	Jiaxian Red (2/5)	Jiaxian Red (1/5)
Nari Master (1/4)		Jinjiang (3/4)	Kazakh (2/9)
Red Sindhi (1/3)		Leiqiong (3/3)	Lohani (1/1)
Sahiwal (5/7)		Lingnan (6/7)	Mongolian (1/7)
Vechur (1/1)		Luxi (5/5)	Nari Master (2/4)
		Nanyang (3/3)	Red Sindhi (2/3)
		Sichuan Indigenous (1/1)	Sahiwal (2/7)
		Wandong (2/2)	Tharparkar (8/8)
		Wannan (3/7)	Zebu Indian (1/1)
Hereford (1/48)		Weining (3/4)	Composite (2)
Unknown (1)		Wenshan (4/6)	Galloway Belted (1/3)
		Xuanhan (2/5)	Shorthorn (1/1)
		Zaobei (4/5)	Hereford (2/48)
			Unknown (1)
		Holstein (5/267)	
		Unknown (3)	

^a^N = total number of animals in the cluster, p = No. of animals within breed in the haplogroup, q = total No. of animals within the breed, *I1-Orig = remaining animals under I1 haplogroup after assignment of other animals to I1a and I1b.

The third I1 cluster, I1-Orig, consisted of the remaining 38 animals under I1 haplogroup in which the private variants specific to I1a and I1b were not present. The cluster I1a was mainly composed of Chinese *indicine* breeds except for two Buryat animals (Russia), while I2, I1-Orig and I1b were mostly *indicine* breeds from the Indian subcontinent and Chinese *indicine* breeds (Table 6).

Further, to explore the substructure of the I haplogroups revealed by the phylogenetic tree, the mitogenomes of only the animals assigned to haplogroup I (by MitoToolPy) were reanalysed using PCA of the GRM, Admixture and hierarchical clustering. The PC plot also showed sub-grouping of the I1 haplogroup into three well-separated clusters that were distinct from I2 (Fig. 4b). Similarly, using Admixture with k set to 2, 3 or 4, there was distinct substructure within the I haplogroups (Fig. 4c). With *k* = 2, Admixture separated I2 and I1, and with *k* = 4 there was further clear separation of I2, I1a, I1b and I1-Orig, in agreement with the PC plot.

The hierarchical clustering analysis (based on animals' pairwise nucleotide differences) showed two main clusters (1 and 2 in Fig. 4d). Further, distinct sub-clusters were observed within both Cluster 1 and Cluster 2, with three main subclusters under I1 haplogroups that matched those identified from the other methods (Fig. 4d). The I2 cluster showed one outlier that was in agreement with the PC plot outlier (i.e. the same animal). In all the above unsupervised clustering analyses, sub-clusters I1a and I1b were comprised of the same group of animals. Interestingly, all three unconventional mitochondrial clustering methods reproduced the same grouping of these animals as with maximum likelihood method.

### Mitochondrial haplotype diversity

**Figure 5:**
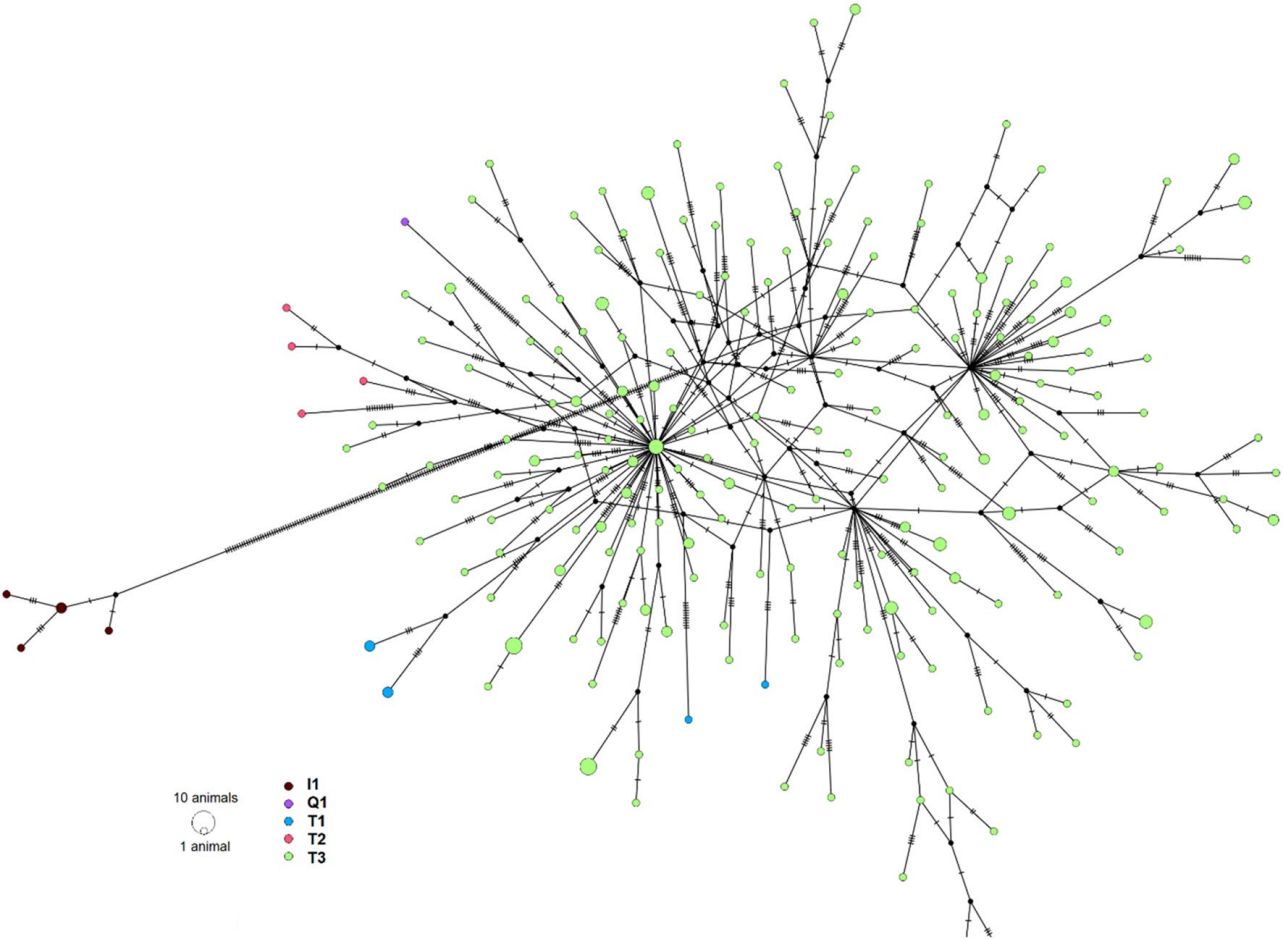
Haplotype network consisting of 210 haplotypes in the Holstein population (N = 267) using the median-joining network in PopART and annotated with haplogroups predicted from MitoToolPy. The size of the circles is proportional to the number of animals carrying the same haplotype.

Overall, across 1883 animals, 1309 whole mitochondrial genome haplotypes were identified. Haplotype diversity (*Hd*) which is calculated based on the number of haplotypes and the proportion of the population under each haplotype was high (0.999, SD 0.0001). Of the 1309 haplotypes, 1010 were singletons (i.e., one animal per haplotype) indicating considerable diversity. The remaining haplotypes (299) were shared by 2–23 animals (Fig. S9). The shared haplotypes were approximately 60% within a breed and 25% between the breeds. The haplotype diversity within breed was generally high and ranged from 0.932 to 0.998 (Table 7). The shared haplotypes specific to a breed were also found across animals sampled in several different countries. Two haplotypes distinct to Angus were present in animals sourced from Canada and USA. Additionally, some haplotypes were shared among several breeds and across several countries. For example, one haplotype was identified in 23 animals from a wide range of breeds including Holsteins sourced from China and a number of other breeds mostly of Asian origin (Luxi, Lingan, Zaobei, Weining, Wannan, Jian, Jinjiang, Wenshan, Nanyang, Xuanhan, Leiqiong and Bohai Black). Similarly, another haplotype was shared by 23 animals in Angus (Canada), Brown Swiss (USA), Charolais (France), Deutsches Schwarzbuntes Niederungsrind (Germany), Gelbvieh (Canada), Hereford (Australia, Russia and USA), Holsteins (Netherlands and USA), Rodkulla (Sweden), Red Angus (USA), Hanwoo (Korea), Belgian Blue and a composite breed (Australia). Among the breeds, Holstein was the most numerous breed in our study (N = 267), therefore it was of interest to examine the network of haplotypes within Holsteins from a total of 210 haplotypes (168 singletons and 42 shared) (Fig. 5). Haplotypes from T3 and subgroups formed the core of the network with side branches in agreement with MitoToolPy I and T1 haplogroup allocations.

**Table 7:** Mitochondrial DNA sequence polymorphism and diversity (standard deviation) of selected breeds with a sample size of 20 or more.

Breed	No. of Sequences	No. of segregating sites	Average no. of difference	No. of haplotypes (H)	Haplotype diversity (Hd)	Nucleotide diversity (π)
Holstein	267	697	16.96	210	0.998 (0.001)	0.0055 (0.0010)
Jersey	27	57	9.62	16	0.937 (0.031)	0.0031 (0.0004)
Brown Swiss	84	202	8.99	64	0.993 (0.003)	0.0029 (0.0001)
Simmental	32	80	7.55	24	0.976 (0.002)	0.0025 (0.0002)
Norwegian Red	222	338	10.51	180	0.998 (0.001)	0.0034 (0.0001)
Holstein Friesians	35	121	9.94	31	0.992 (0.010)	0.0032 (0.0003)
DSN^a^	47	154	10.2	40	0.992 (0.007)	0.0033 (0.0002)
Angus	103	122	7.65	45	0.935 (0.014)	0.0025 (0.0001)
Yakut	35	68	9.44	15	0.938 (0.018)	0.0031 (0.0003)
Hereford	44	312	33.84	33	0.979 (0.012)	0.011 (0.0043)
Charolais	33	114	8.856	31	0.996 (0.009)	0.0028 (0.0002)
Limousin	27	100	8.80	25	0.994 (0.012)	0.0029 (0.0002)
Modern Danish Red	23	73	11.55	19	0.980 (0.020)	0.0038 (0.0002)
Hanwoo	24	146	15.59	23	0.996 (0.013)	0.0051 (0.0013)
Buryat	20	250	48.16	12	0.932 (0.035)	0.0157 (0.0070)

^a^DSN Deutsches Schwarzbuntes Niederungsrind.

In addition to the established methods and tools to identify haplotypes, this study tested the ability of a naïve hierarchical clustering approach to differentiate haplotypes across all animals based on the nucleotide difference between each pair of individuals. We used the cut-off height of the cluster (*h*) corresponding to the nucleotide difference of 0 between two haplotype pairs. This method resulted in approximately the same number of singleton haplotype clusters as singleton haplotypes (1032) determined from the DnaSP software. At least 132 clusters and haplotypes had substantial memberships in common (Table S7) to the haplotypes from DnaSP. For example, Cluster 328 and Haplotype-324 (with 23 each), Cluster-7 and Haplotype-7 (21 animals each) and all other cluster-haplotype combinations with more than five animals (total 30) had 100% of the same individuals except for five groups. This demonstrates a high concordance between determination of haplotypes by hierarchical cluster and the traditionally determined haplotypes.

### Mitochondrial DNA polymorphism and nucleotide diversity

We investigated mitochondrial nucleotide diversity in animals from breed groups with N ≥ 20 animals. Overall, there were 1825 segregating sites, nucleotide diversity (π) was 0.012, and the average nucleotide difference between the pair of sequences was 35.5. The nucleotide diversity within breed was high in Buryat and Hereford compared to other breeds contributing substantially to the overall nucleotide diversity. Other breeds had low but comparable nucleotide diversity ranging from 0.002 to 0.005. The analysis of molecular variance (AMOVA) showed that the percentage of genetic variation from among and within breed components was 3.1% and 96.9%, respectively, indicating high within breed genetic diversity.

### Imputation and MitoToolPy haplogroup prediction

The routine practice of discarding the sites with missing genotypes from all sequences in the mtDNA analysis results in loss of information, particularly when the proportion of missing genotypes in an individual were low. In this case, the imputation of sporadic missing genotypes could increase the number of animals and sites for analysis, but the empirical accuracy of mitochondrial imputation in cattle is unknown. To test this, we masked 10% of known genotypes (307 sites) in a random 20% of animals (333 out of 1,883). Then we imputed the masked genotypes using Beagle (version 4.0) using the *gt* and *ref* option using the remaining 1550 animals with all genotypes present as a reference for imputation. The overall concordance of this imputation was 99.8%, although concordance for heteroplasmic sites was approximately 66% (Table 8). There was a tendency for imputation to bias heteroplasmic and homoplasmic alternate genotypes towards the homoplasmic reference genotypes (0/0). The genotype likelihood ‘*gl*’ option also produced a similar concordance of 99.5%.

**Table 8:** Empirical accuracy of imputing sporadic missing genotypes in mitogenomes. Number of correctly imputed genotypes (percentage correct in brackets) on the diagonals and number of genotypes wrongly imputed shown on the off-diagonals. Assessment was based on randomly masking of 10% of positions (307) per animal in 20% of animals (333).

**Imputed genotype**	**Original genotype**						Total genotypes corectly imputed
	0|0	0|1	0|2	1|1	2|2	3|3	
0|0	101,089	39		73		1	
	(99.9%)						
0|1	37	82		11			
		(65.6%)					
0|2	2		3		2		
			(100%)				
1|1	64	4		800	1		
				(90.4%)			
2|2	4			1	16		
					(84.2%)		
3|3						2	
						(67.0%)	
Total	101,196	125	3	885	19	3	
							101,992/102,231 (99.8%)

To evaluate the effect of imputation on haplogroup prediction, we re-analysed the animal haplogroups in the imputed dataset and compared these to their haplogroup prediction from the original dataset. This was replicated 50 times with a new random set of animals chosen for masking genotypes for imputation. The predicted haplogroups matched in 99.7% of the individuals when compared to their haplogroup predicted from the full set of real genotypes. The accuracy of imputation and the predicted haplogroup for the masked dataset showed little variation across the 50 replications (Table S8). This suggests that missing genotypes can be imputed and used for prediction of haplogroups with high but not perfect accuracy. These results are provided for information only, that is, no imputed data was used elsewhere in this study.

## Discussion

Our study undertook a comprehensive analysis of mitochondrial genome sequence diversity in 1883 cattle, including the most important global cattle breeds and sub-species in a single study. This represents one of the single largest studies of this kind demonstrating the use of short read mitochondrial sequence data from general whole genome sequencing after stringent quality controls. Our use of the entire mitogenome enabled a more in-depth study of the full range of diversity, that may be important in future studies of the potential impact of mitochondrial variants on phenotypes. Our large sample size enabled identification of all major haplogroups (I1, I2, P, Q, T1 to T6) except for R, subgrouping within haplogroups and breeds as well as annotation of the private variants. The R haplogroup is relatively rare and previously has been identified occasionally in Italian breeds^30,55^. The low representation of P, Q, T4, T5 and T6 was also in line with a recent study by Cubric-Curik et al.^30^. Like us, Cubric-Curik and colleagues found that MitoToolpy assigned T6 to some Angus animals, but they reported no statistical evidence for T6 using BEAST software^56^ which assigned the same animals to the T3 group (agreement was good for most other haplogroups). In agreement with Cubric-Curik et al.^30^, we recommend using whole mitogenomes with MitoToolpy because some haplogroups failed to separate using only the D-loop region. In addition to the conventional diversity indices, we have investigated alternate ways of analysing population structure and haplotypes of the entire mitogenome, that do not rely on predefined haplogroups and therefore capture a broader spectrum of the diversity.

### Introgression of African taurus and indicus haplogroups into European taurus

Most breeds belonged to their anticipated haplogroup except for some animals of European breeds and composites that were mostly allocated to African taurus (T1) and relatively few to indicus (I). This is not surprising, as the T1 haplogroup has been previously reported in European cattle breeds (1–30%) from France, Spain, Portugal, Italy, Balkan and Greece^5,19,57,58^ and America (Creole cattle)^59^. The detection of T1 haplogroups in Iberia^60^ and Sicily and southern Italy, according to^61^ may be the influence of migration of African cattle into southern Europe via the Mediterranean Sea coastline. The African T1 sequence was also found in Iberian Bronze age cattle^62^. Interestingly, although all African breeds were assigned as T1, we observed that their nuclear DNA was an admixture of *taurus* and *indicus* (Fig. S10) as reported in a recent study^63^.

The breeds with no previous report of T1 haplogroup but found in our study are Jersey and Holstein: those showing the T1 haplotype were sourced mainly from Australia (9 out of 10). Australia has a recent history of crossbreeding European breeds with more heat-tolerant imported breeds to develop cattle better adapted to the tropical environment in northern Australia^64^. Australia imported Jersey from the Channel Islands and Holsteins from the Netherlands in 1850. It is possible that some of the first African cattle arriving in Australia were Afrikander (8 bulls and 2 cows) imported from South Africa in the early 1950s^65^ and other breeds (Boran, Bonsmara etc.) in the late 1980s. However, details on the sex of imported animals are not available making it difficult to confirm the most likely maternal route of T1 mitogenome transmission. In 1990, the embryos from Boran and Tuli (African) cattle were imported^66^. While the attributes of heat tolerance and tick resistance were sought after under the extensive tropical beef production system, the presence of the T1 haplogroup in dairy breeds (Holstein and Jersey) in Australia suggests these animals may be the result of upgrading from cows carrying the T1 mitochondrial lineages or sporadic cases of cross breeding to improve heat tolerance but this warrants further investigation.

The *indicine* haplogroup (I) in Holsteins in this study were largely in female samples originating from China. This is not surprising as the I haplogroup has been previously reported in Chinese Holstein^67^ and at least three haplotypes were shared among Chinese Holstein and native cattle (22 animals)^68^. Imported purebred Holsteins were used to grade-up local cows as well as for the development of the Chinese Black and White cattle breed^69^.

There is a possibility that the breed origin was incorrectly labelled on some samples, therefore we undertook a PCA of all the taurus animals (N = 1451) based on a genomic relationship matrix derived from 45,000 autosomal SNPs. The PC plot of Holstein and Jersey breeds shows tight clustering of these animals regardless of the MT haplogroup (Fig. S11), which supports that the *indicine* and the African *taurine* maternal lines in these breeds are likely due to upgrading.

In composite breeds such as Brahman, the mitochondrial haplotypes were mostly *taurine* in this study, which is interesting because the breed's nuclear DNA is primarily of indicus origin in the 1000 Bull Genomes project^31^. The Australian Brahman cattle in this study were approximately 97% *taurine* (T1 47% and T3 50%) and about 3% indicus (I) haplogroups. Compared to our study, Brahman from China were reported with lower representation of T1 (35%) and T3 (26%), but higher in I (39%) haplogroups^29^, while American Brahman showed lower T1 (30%) but higher T3 (70%)^70^. Originally, Brahman cattle were introduced into Australia from the USA in 1933^64^. In fact, in America, Brahmans were developed from the crossing/upgrading of B. taurus females (often Creole cattle) with Guzerat, Nellore, Gyr and Krishna valley cattle. As such, haplogroups in the *indicine* breeds in Americas were reported to be largely *taurine* (T3 50%, T1 48%) and rarely *indicine* (I) (1 in 66 *indicine* animals)^71^.

The inter-breed introgression of haplogroups was also supported by sharing of the diverse haplotypes among breeds. This again points to the common practice of upgrading. Mitochondrial haplotypes were also shared across countries, and to a higher degree between countries in close proximity, indicating the movement of female animals. However, the shared haplotypes between more distant countries (e.g. USA and Australia) suggests the movement of foundation females or, more recently, embryos.

### Subgroup of II Chinese *indicus* (I1a)

The presence of both I1 and I2 indicus haplogroups with the predominance of I1 recorded in the current study agrees with the previous studies^72–74^. The I1 haplogroup originated in the Indus valley, while the I2 haplogroup is believed to have originated in northern India^75^. Interestingly, within the large subcluster of I1, we consistently identified a sub-cluster (I1a) comprising mainly of Chinese *indicine* breeds (19/20 breeds) (Fig. 4). This sub-cluster (I1a) had two mutations specific to the subgroup, one in a rRNA and another within the ATP6 genes. These mutations were annotated as non-coding transcript exonic and missense variants, respectively. There has been a previous report of specific I1 haplotype common among the Chinese breeds indicative of a nucleus of Chinese indicus, but this was based on D-loop sequences^73^. Another study employing whole mitochondrial genome also reported a specific group under I1 (characterised by 6 mutations) for a breed not in the current study (Yunling cattle)^29^. The two specific mutations characterising the I1a subgroup in our study were also reported in the Yunling cattle subgroup, while the other four were found non-specific to I1a group in our study. These findings, together with results from our study, suggest the presence of a unique I1 subgroup (I1a) specific to breeds emanating from China. Further, five Holstein animals in our study that originated from China also had the I1a sub-haplogroup, indicating there may have been *adhoc* or controlled upgrading of indicus females that carry I1a.

While I1a in this study may not be a separate haplogroup, a distinct cattle haplogroup "C" and a separate domestication event in north-eastern China during the early Holocene has been proposed by^76^. However, their proposed new haplogroup C sequence did not match our I1a subgroup sequences. The presence of conventional I1and I2 haplogroups support the consensus among the published literature that the indicus cattle population in China is a result of migration and spread from India. The *Bos indicus* are reported to have been introduced into China between 2000 and 200 BC and currently there is no zooarchaeological or genetic evidence for the origin of domestic cattle in ancient China^72,77^ suggesting genetic drift as a contributing factor to the formation of the subgroup (I1a). Another possibility, considering the specificity to Chinese breeds (not present in Indian *indicine* breeds), is the potential restocking of auroch female lines from the wild in China and establishing nucleus or base for the *indicine* breeds in China. There is molecular evidence of aurochs in China's northeast during the Neolithic period^78^. Therefore, these hypotheses need further investigation. The subgroup (I1b) under I1 characterised by a specific mutation (5707 bp) did not exist in any breeds from China, while the entire I1 group included breeds from both India and China. There are shared haplogroups (I1 and I2) between breeds of the two countries and also sub-groups specific to the region.

There are fewer studies within *indicine* haplogroups compared to *taurine* and previous studies classified them into only two broad haplogroups (I1 and I2). Despite several previous studies on mtDNA of the Chinese cattle, subgroupings under I1 were not reported except by^29^. One possible reason is that the location of the mutations defining subgroup under I1, is in the coding region of the mitochondrial genome, while most studies in the past were mainly based on the D-loop. Complete mitochondrial genome sequences better define the full spectrum of mitochondrial diversity compared to using the D-loop only, and may uncover mutations in coding regions that affect specific phenotypes.

### Imputation of mitogenome variants

Genetic variation was mainly within breed (97%, AMOVA), and high haplotype diversity and multiple haplogroups exist within breeds. For example, Holstein animals (N = 267) belonged to at least 15 subgroups and 210 haplotypes (Table 7, Fig. 5). The haplogroup and haplotypes within a breed are of strong interest for phenotype association studies and in this study, we found several private variants in groups of animals that were annotated as missense in MT genes (Table S5). In humans, the association of mitochondrial haplogroups (H and R) to specific phenotypes such as risk to diseases^79–81^, metabolic disorders^82,83^ and athletic endurance^84,85^, is more advanced than in domestic animals. Although two recent studies examined the relationship between mitochondrial haplogroups and litter size^23^ and other phenotypic traits^24^ in pigs, these studies lack sufficient power to distinguish or pinpoint specific mutations (causal) affecting the trait.

Association studies of traits could include variants from the mitochondrial genome, if it is possible to sequence and impute large numbers of animals for MT variants, but this has not yet been done in livestock. While whole-genome sequences are now regularly imputed and exploited for association studies of production traits across livestock, mitochondrial genomes are excluded from these studies and literature on mitochondrial genome imputation is scarce or non-existent for livestock. In humans, the imputation of the mitogenome from ancient remains showed that the accuracy of mitogenome imputation, like the nuclear counterpart, benefitted from having a large and diverse reference sequence^86^. Thus, utilising the existing resources such as the data from the 1000 Bull Genomes Project could potentially provide a reference set for mitogenome imputation from lower density SNP arrays. However, we would recommend following our rigorous filtering to minimize the impact of NUMTs and wherever possible using only non-semen male tissue samples or female tissue samples. The first step towards large scale imputation of MT sequence is to confirm that sporadic missing genotypes in the mitogenomes can be accurately imputed. This would then provide a complete and large mitogenome reference population to use for imputation of animals with lower density MT SNP panel genotypes up to MT sequence genotypes. In the current study, the accuracy of imputation of sporadic missing genotypes (99.8%) was comparable to results in humans that used tools specifically for imputation of the mitochondrial genome such as MitoIMP^87^. This indicates that existing tools may be applied to mitochondrial genome imputation with customization. We are currently investigating the accuracy of imputation of mitochondrial SNPs from lower density SNP panels to mitochondrial sequence.

### Applicability of unconventional mitochondrial DNA analysis tools

The current study utilised conventional tools for mtDNA analysis (DnaSP, MitoToolPy, MEGA X) but also compared these results with alternative tools such as GRM based PCA, Admixture, and hierarchical clustering based on nucleotide differences. Our primary interest in use of the alternative tools was to better quantify the full spectrum of genetic diversity across the entire mitogenome, rather than simply place animals into the higher level haplogroups. However, as expected, the results from the less conventional mitochondrial tools were mostly in agreement with haplogrouping. Therefore, alternative clustering methods, specifically the hierarchical clustering based on nucleotide differences, may be used as grouping techniques that is equivalent to haplotypes for use in trait/phenotypic association studies.

## Conclusions

There is high mitochondrial genomic diversity among modern cattle and a large proportion of this genetic variation is within breeds. The introgression of African *taurine* and *indicine* mitochondrial haplogroups into European *taurine* breeds occurred at low frequencies. The patterns of population structure and haplogroups from conventional tools were very similar to results of non-traditional mitochondrial methods developed for autosomal DNA. We provide additional evidence of a new indicus I1 haplogroup subgroup (I1a) in Chinese *indicine* breeds. Within breed mitochondrial diversity (haplotypes/ haplogroups) is likely at a level sufficient to conduct trait association studies. Imputation of sporadic missing genotypes in the mitochondrial genome was highly accurate with the exception of heteroplasmic sites. This could enable larger data sets to be used for population studies through recovery of sites or animals with low levels of missing genotypes. Potentially, it would also provide a diverse reference population for large scale imputation of mitogenomes from lower density panels with MT SNP.

## Supplementary Information

Supplementary Figures.

Supplementary Information 1.

Supplementary Information 2.

Supplementary Tables.

Supplementary Tables.

Supplementary Tables.

## Data Availability

A large number of the animal sequence genotypes (1832) are available at the European Variation Archive (PRJEB42783) and we thank all institutions and members of the 1000 Bull Genomes Project that have deposited their sequences in public archives. This study makes 958 mitochondrial fasta genome sequences available that are critical to the findings of the study as Supplementary Tables S9 and Supplementary File 1 (Biosample ID are provided for access to fastq data). Some sequences are not yet public but access to the data could be requested through membership of the 1000 Bull Genomes Project.
