# Supplementary material for: Recovery of mitogenomes from whole genome sequences to infer maternal diversity in 1883 modern taurine and indicine cattle
Source: Sci Rep. 2022 Apr 4;12:5582. doi: 10.1038/s41598-022-09427-y (PMC8980051; doi:10.1038/s41598-022-09427-y)
Supplement: Supplementary file 1 — Supplementary Figures. [file 41598_2022_9427_MOESM1_ESM.pdf]

Supplementary Figures

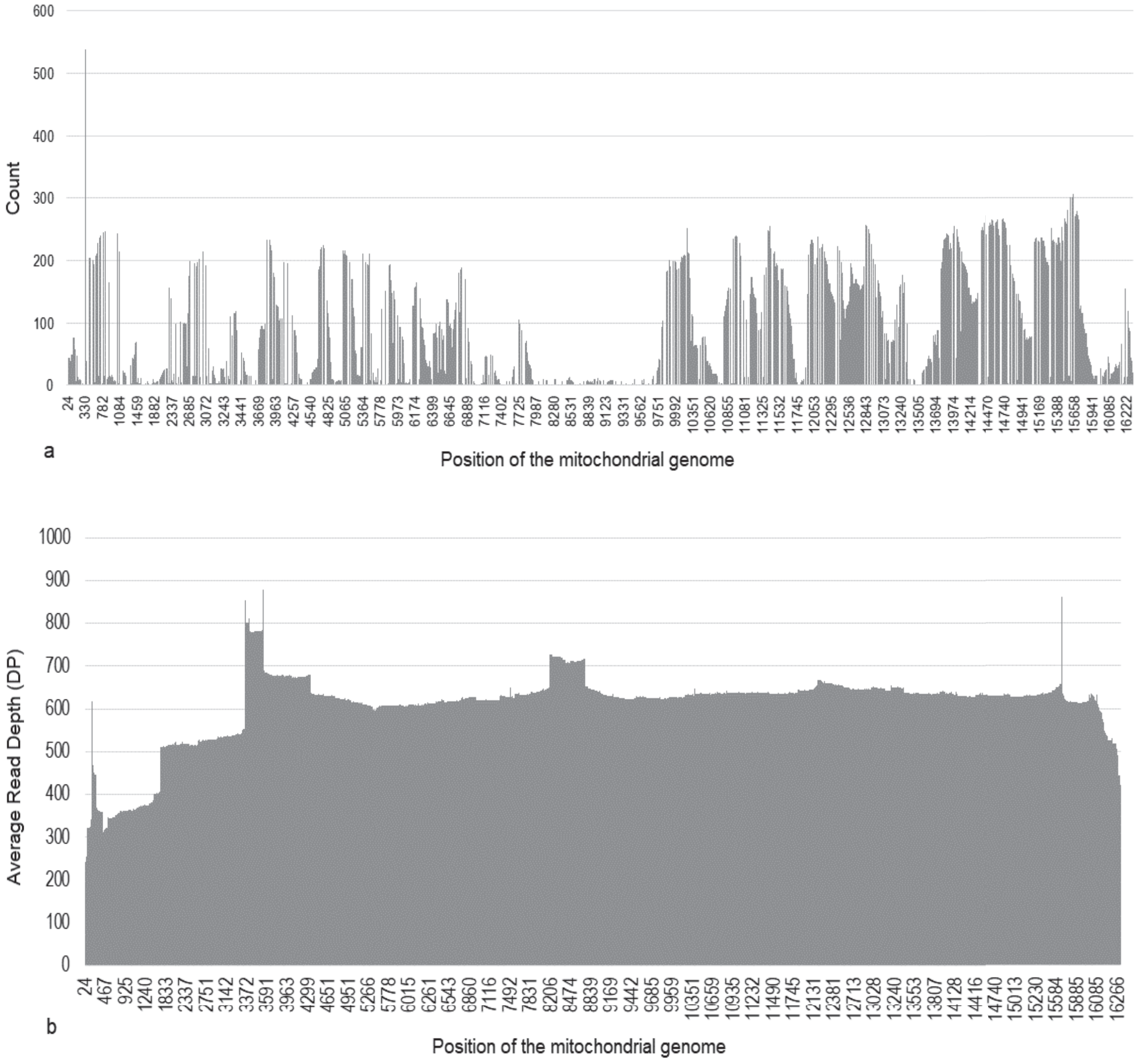

Figure S1. Distribution of heteroplasmic mitochondrial variants and average read depth in the filtered dataset of 2176 animals (sites with missing genotypes removed). Count of heteroplasmy in a position on cattle mitochondrial genome (a) and mean read depth (DP) across all animals at the positions in the mitochondrial genome (b).

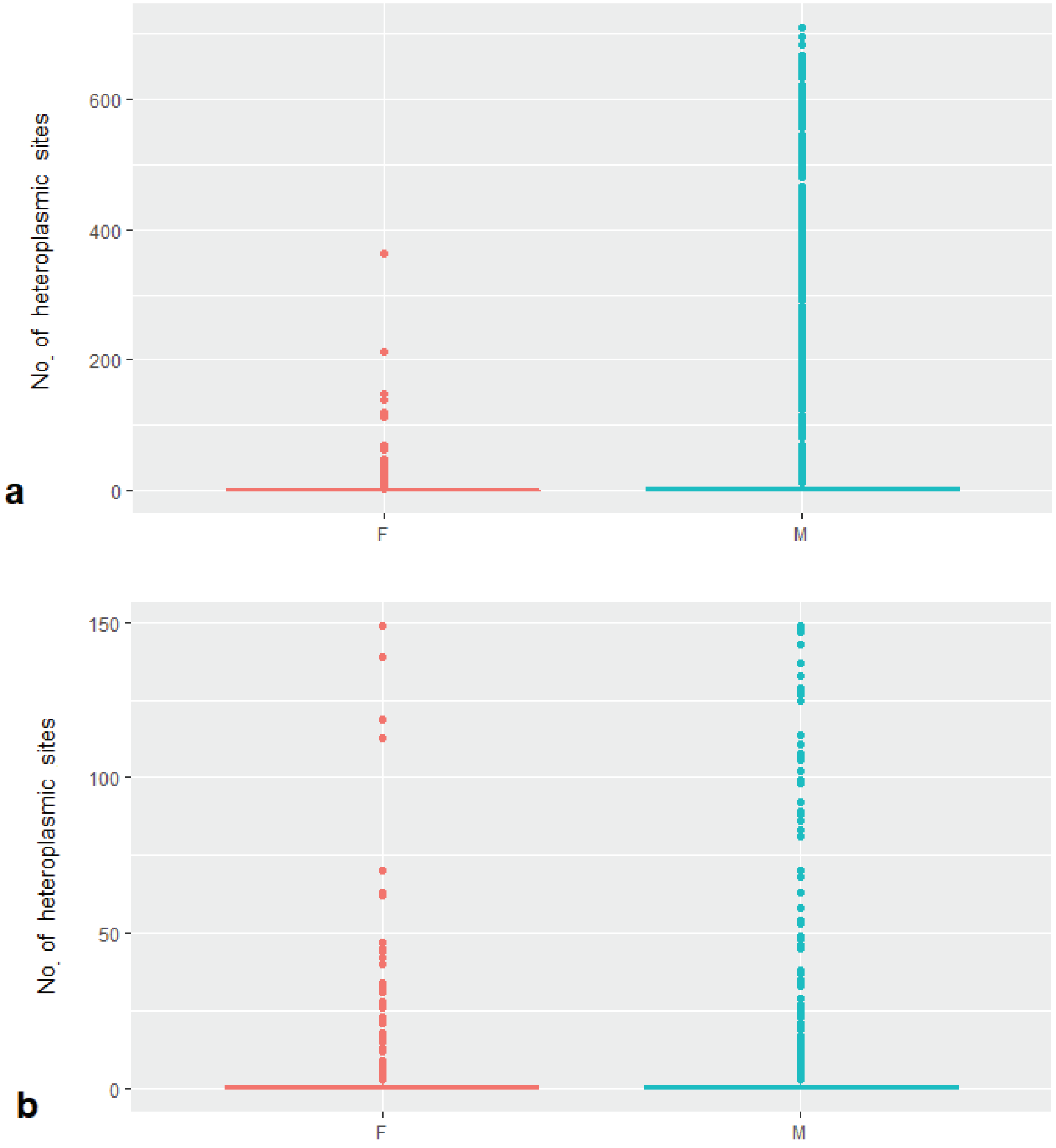

Figure S2. Box plots of the number of heteroplasmic sites per animal in individuals of two groups; F (non-semen: females and males with non-semen tissues sampled for DNA extraction) and M (males with either semen for DNA extraction or unknown tissue sample origin) before (a) and after (b) filtering out animals with > 150 heteroplasmic sites.

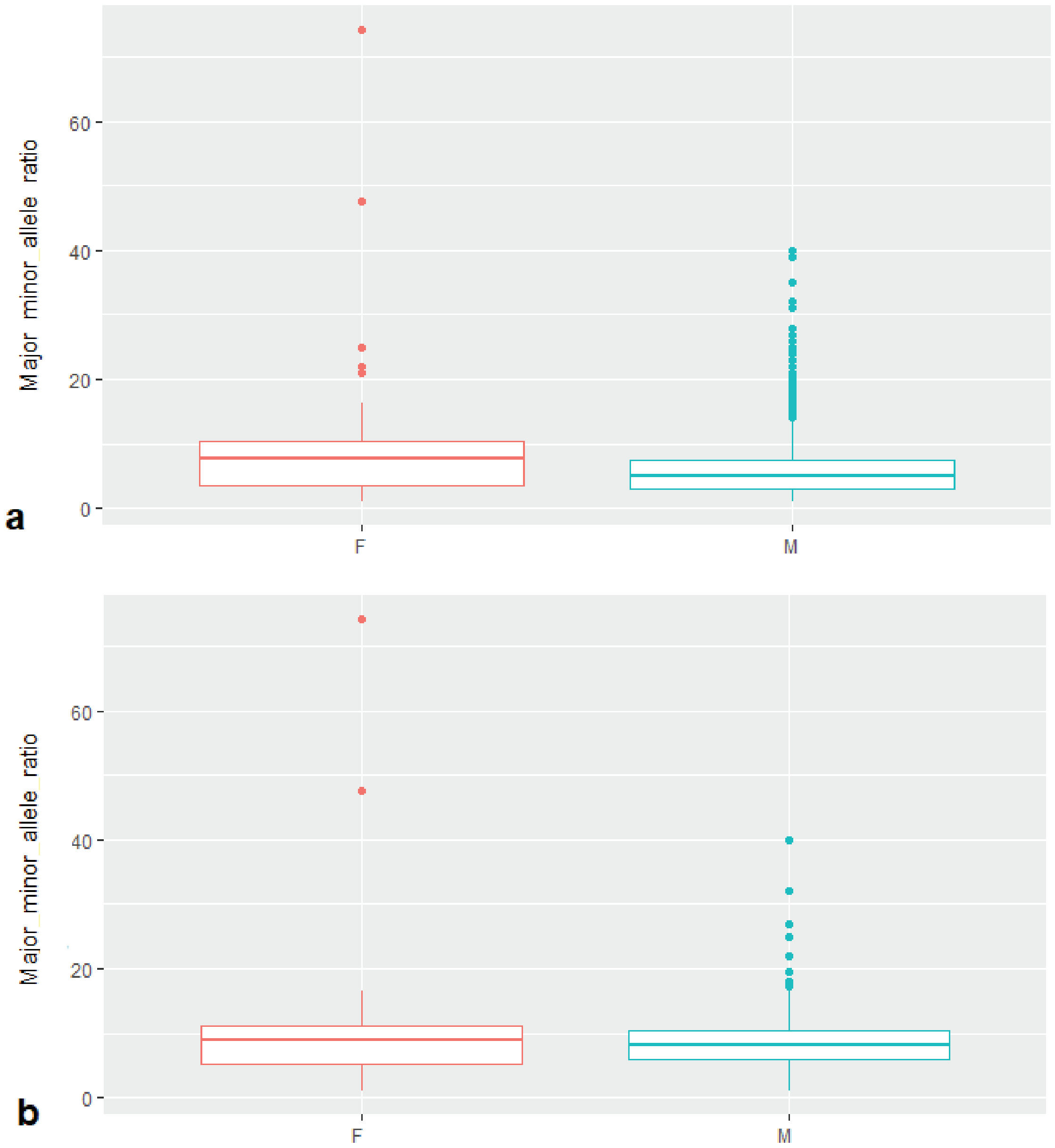

Figure S3. Box plots of allelic depth ratio of major and minor allele at the heteroplasmic sites of animals in two groups F (non-semen: females and males with non-semen tissues sampled for DNA extraction) and M (males with either semen for DNA extraction or unknown tissue sample origin) showing before (a) and after (b) filtering out animals with >150 heteroplasmic sites.

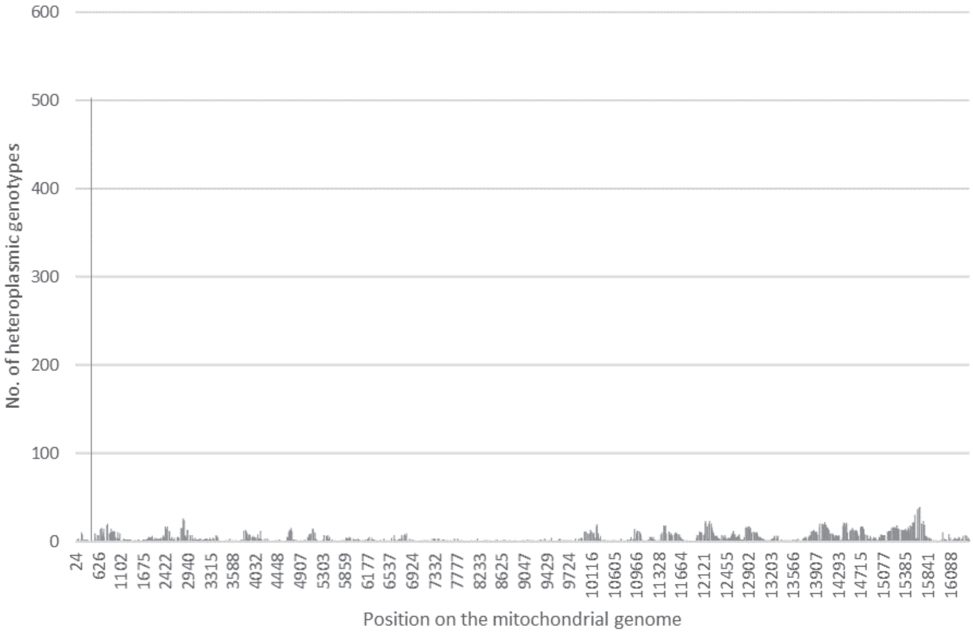

a

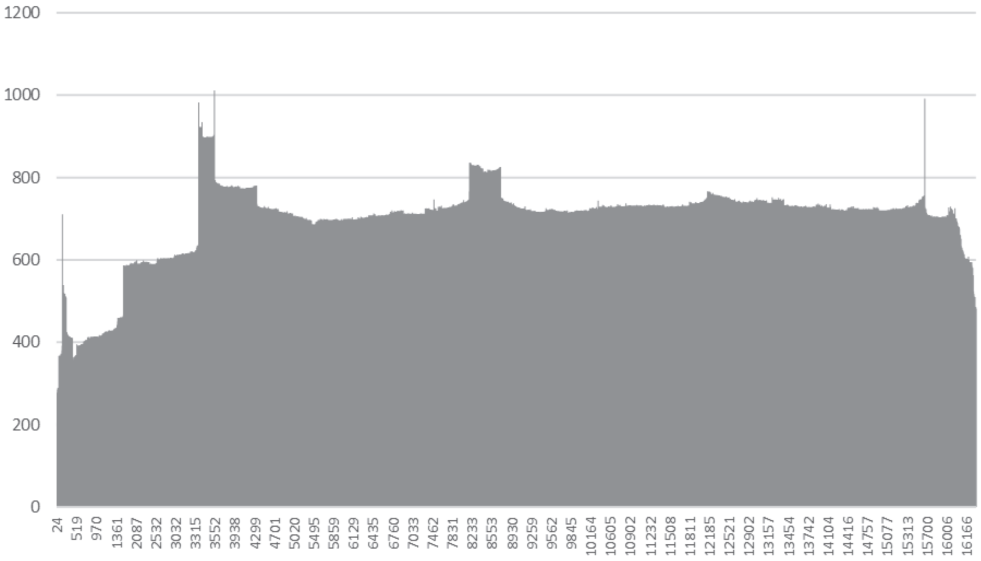

b

Figure S4. Distribution of mitochondrial heteroplasmic variants and average read depth in the final dataset (1883 animals) after removing all individuals with >150 heteroplasmic MT variants. a) Count of heteroplasmy in a position on cattle mitochondrial genome (a). Mean read depth (DP) across all animals at the positions in the mitochondrial genome (b).

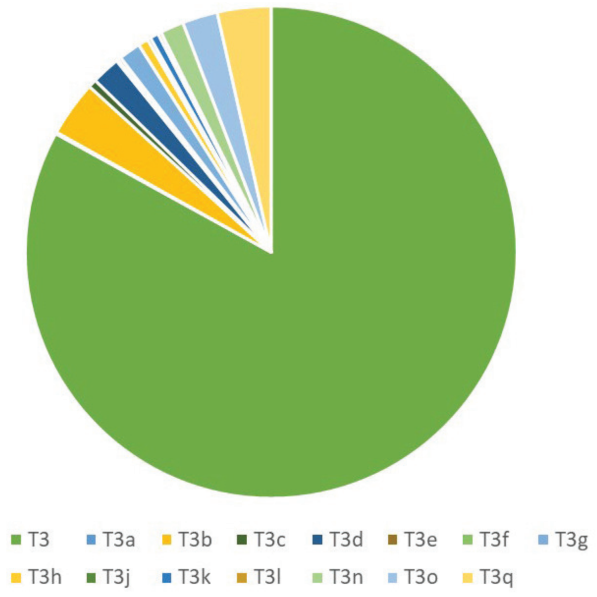

Figure S5. Pie-chart showing the subgroups within T3 major haplogroup and the composition in a total of 1502 animals.

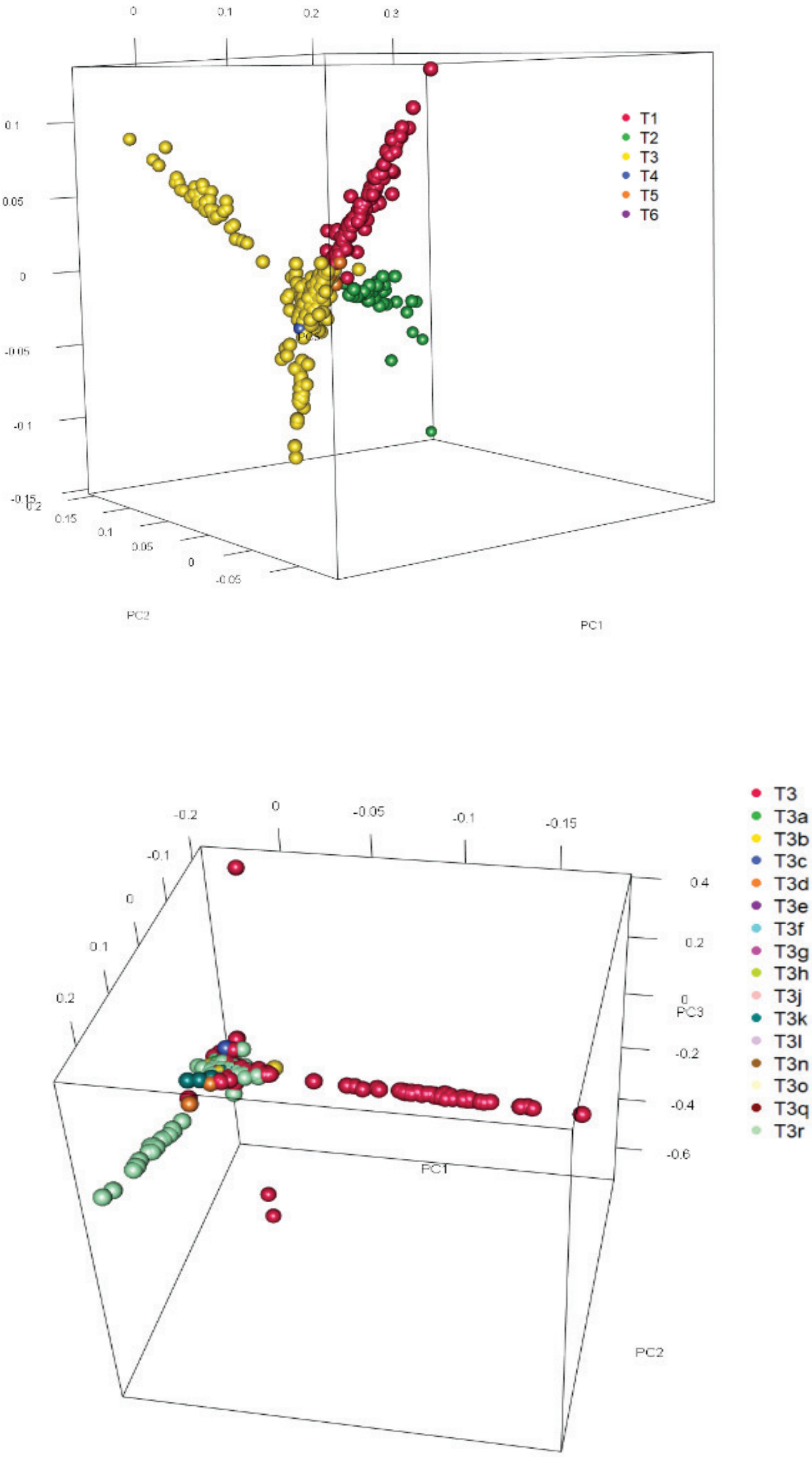

a

b

Figure S6. PC plot of PC1, 2 and 3 based on a genomic relationship matrix derived from whole mitochondrial DNA sequence variants annotated with haplogroup predicted from MitoToolPy. Plot annotated with T haplogroups (a) and T3 (b) sub-haplogroups.

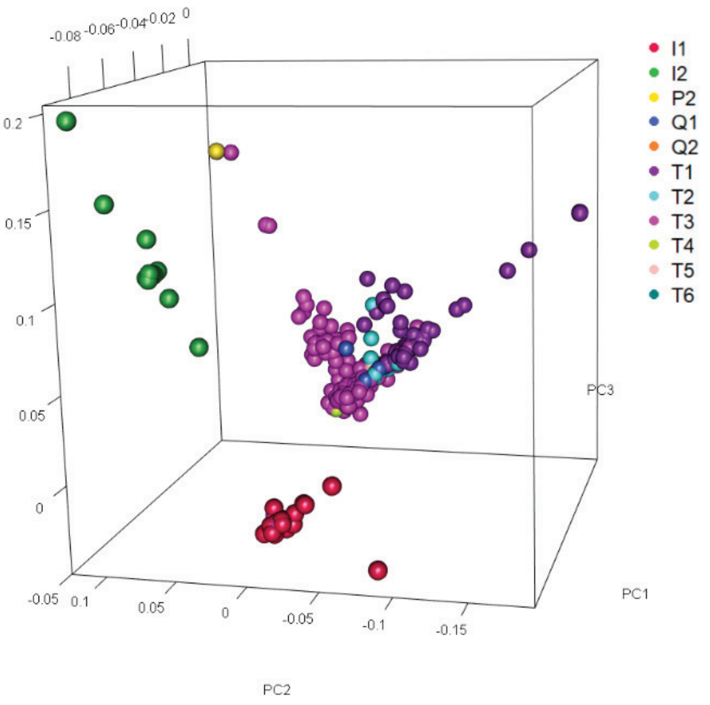

a

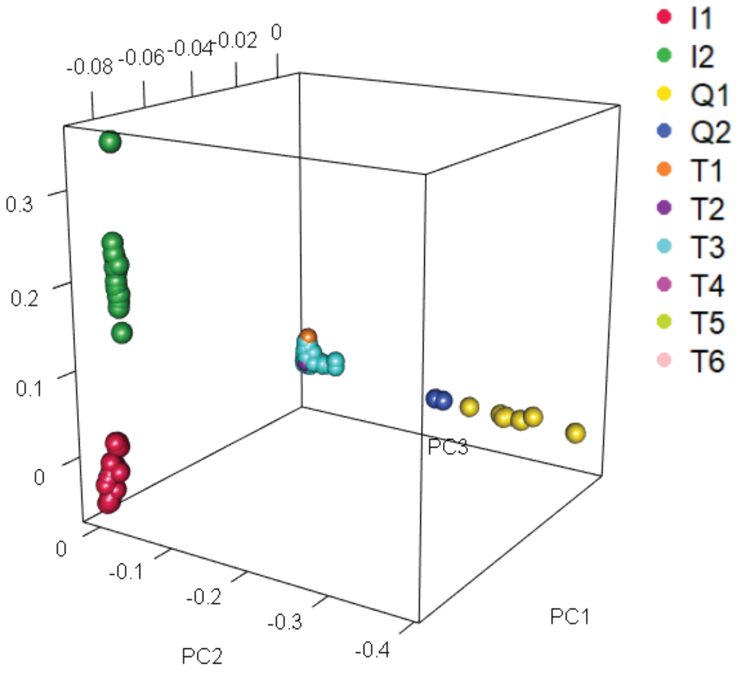

b

Figure S7. PC plot based on the genomic relationship matrix of mitochondrial D-loop sequence variants (a) and variants from the non-D-loop region (b) variant sequences annotated with haplogroups predicted using MitoToolPy.

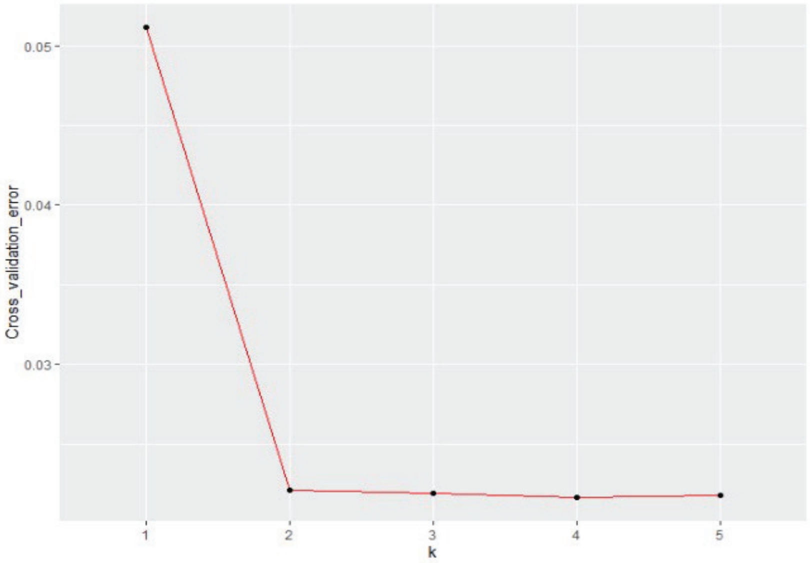

Figure S8. Determining the optimal *a priori* “k” value (i.e. number of population sub-groups) to use in Admixture software for 1883 mitogenomes in our study. Plot shows changes in cross-validation errors using prior values of 1 to 5 for k in Admixture with the optimal value indicating *k*=4.

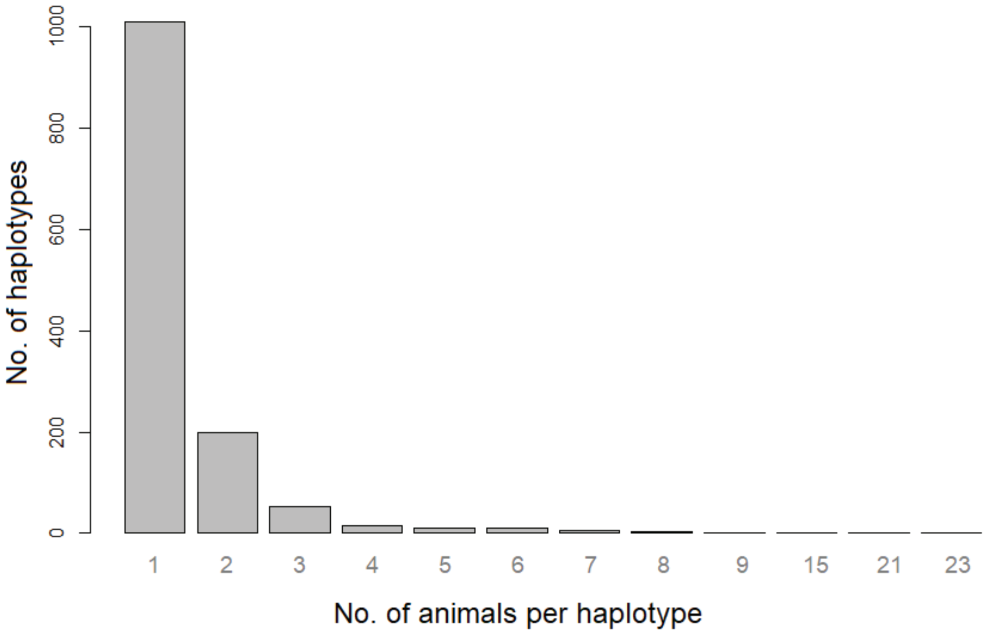

Figure S9. Frequency distribution of haplotypes with the number of animals per haplotype. Haplotypes were defined using all variant sites (excluding indels) across the mitogenome of the study animals.

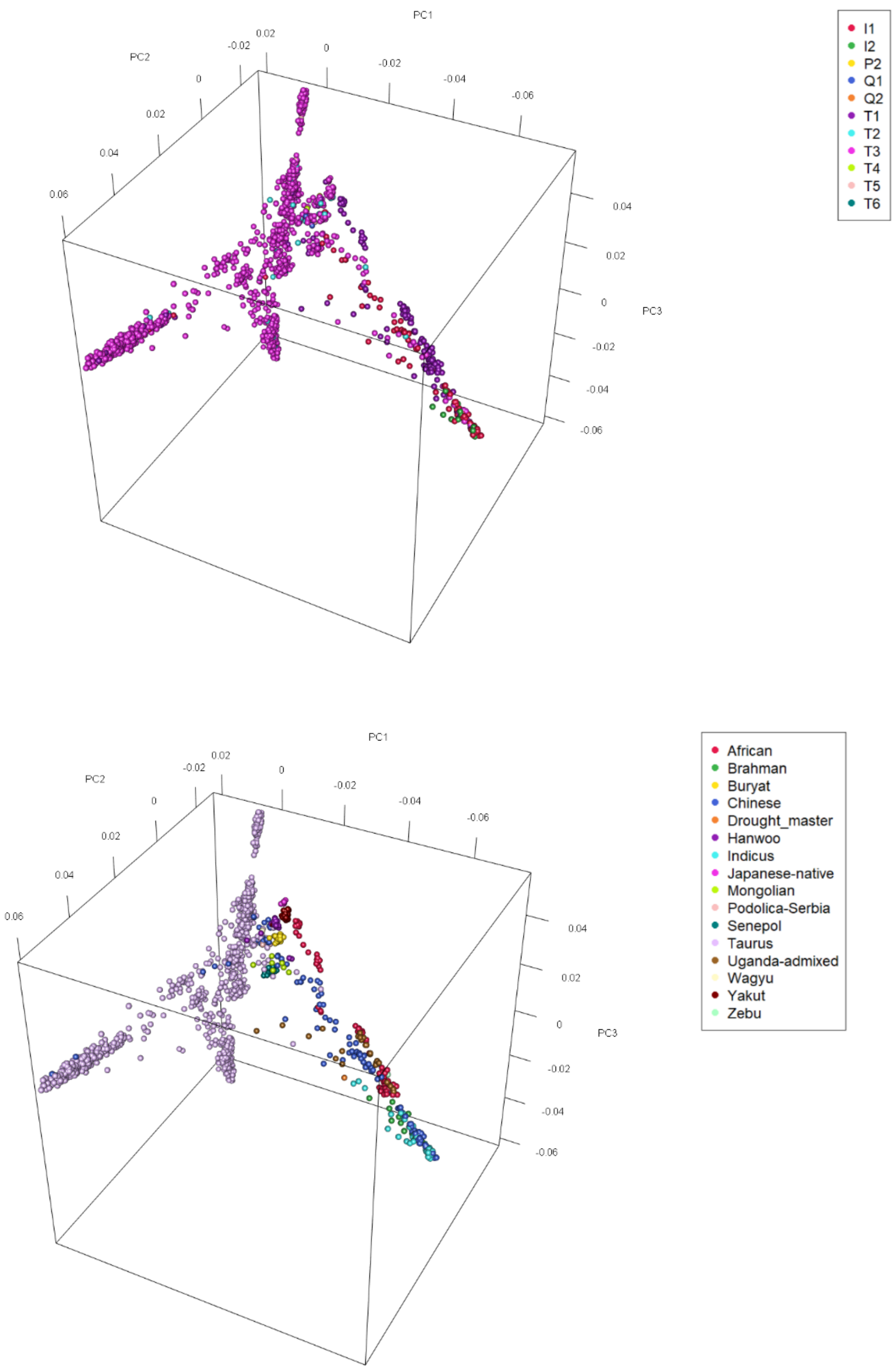

Figure S10. PC plot of animals from selected breeds based on a genomic relationship matrix (GRM) of taurus and indicus animals (N=1657) in the study set (N=1883). Each dot represents one animal and they have been annotated to mitochondrial haplogroups (a) and breeds/regions (b). The GRM was constructed from a custom 45K autosomal SNP panel using GCTA. Although all African breeds were assigned to the T1 haplogroup using their mitogenome, the PC plot using nuclear DNA demonstrates that these animals often appear to be an admixture of *Bos taurus* and *indicus*.

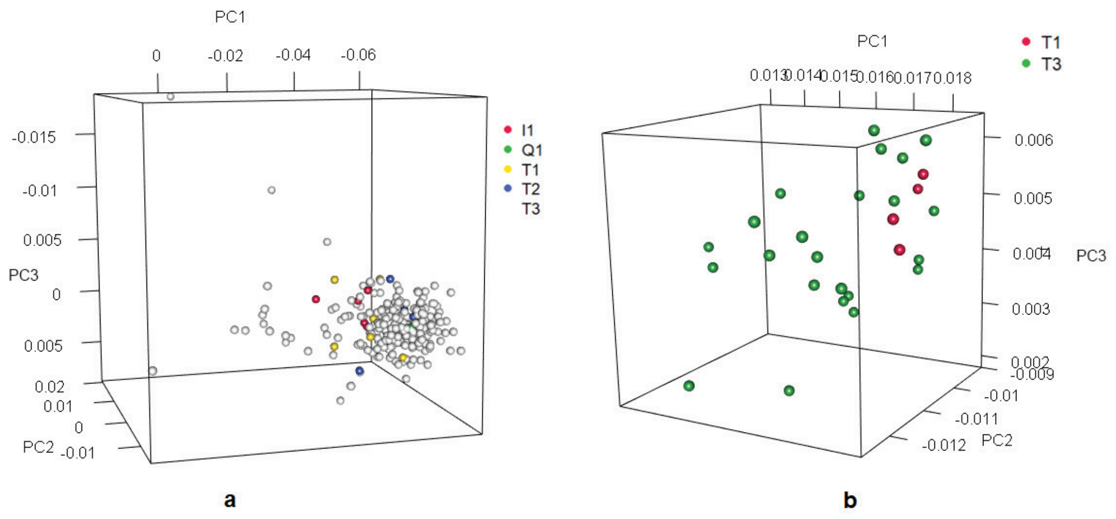

Figure S11. PC plot of Holstein (a) and Jersey (b) breeds based on a genomic relationship matrix (GRM) of the taurus only animals (N=1451) in the study set (N=1883). The GRM was constructed from a custom 45K autosomal SNP panel using GCTA.
