# Supplementary material for: Recovery of mitogenomes from whole genome sequences to infer maternal diversity in 1883 modern taurine and indicine cattle
Source: Sci Rep. 2022 Apr 4;12:5582. doi: 10.1038/s41598-022-09427-y (PMC8980051; doi:10.1038/s41598-022-09427-y)
Supplement: Supplementary file 4 — Supplementary Tables. [file 41598_2022_9427_MOESM4_ESM.pdf]

TableS1: Variants used in prediction of cattle mitochondrial haplogroup lifted over from Bovine Reference Sequence (V00654) to the ARS-UCD1.2_M reference sequence.

RPQT:8,106,166,250,297,301,2560,2981,3441,3552,3602,5503,5745,5892,6438,6774,7332,7358,7516,8190,8372C,8496,9007,
9980,10333,10693G,11002,11136,11421,11844,12236,12470,12471,12624,12677,12686,12752,12803,12902,13007,13058A,
14038,14827,15136,15619,15629,15820,15953,15957G,16059,16123,16124,16139,16250,16257,16303

PQT:106,166,169,250,301,2560,3552,5503,5745,5892,6438,7358,8372C,10693G,11002,12470,12677,12752,13007,14038,15136,
15629,15953,15955G,16124,16257

QT:169,2560,5503,8372C,11002,12470,12677,12752,13007,14038,15136,15629,15955G,16257

Q:169,2560,3240,3417T,5503,7920,8320,8372C,10719A,10929,11002,11091,12435,12470,12677,12752,13007,14038,14110,
15136,15629,15955G,16257

Q1:169,1459,2560,3240,3417T,5503,7920,8320,8372C,10719A,10929,11002,11091,12435,12470,12677,12752,13007,14038,
14110,15136,15629,15955G,16257

Q1a:169,244T,1459,2560,3240,3417T,5503,7920,8320,8372C,10719A,10929,11002,11091,11768,12435,12470,12677,12752,
13007,13622,14038,14110,15136,15629,15955G,16114,16257

Q1a_FJ971083_HQ184034_HQ184035:169,244T,1459,2560,3240,3417T,5503,7920,8320,8372C,8619,10719A,10929,11002,
11091,11768,12435,12470,12677,12752,13007,13508,13622,14038,14110,15136,15955G,16114,16257

Q1_HQ184039:169,471,1459,2560,3240,3417T,5503,5718,6660,7832,7920,8320,8372C,10719A,10929,11002,11091,12435,
12470,12482,12677,12752,13007,14038,14110,15092,15100A,15136,15629,15955G,16198,16257

Q1_HQ184036_HQ184037_HQ184038_EU177866_EU177867:1459,2560,3240,3417T,5503,7920,8320,8372C,8407,10719A,
10929,11002,11091,11478,12379,12435,12470,12677,12732,12752,12879,12926,13007,13821,14038,14110,15136,15629, 15955G,
16060,16081,16257

Q2:169,2560,3240,3417T,5503,6438,7920,8320,8372C,10719A,10929,11002,11091,12435,12470,12677,12752,13007,14038,
14110,15136,15158,15629,15923,15955G,16257

Q2_HQ184030_HQ184031_HQ184032:169,2560,3240,3417T,5503,6438,7920,8320,8372C,8881,10719A,10929,11002,11091,
12435,12470,12677,12752,13007,14038,14110,15136,15158,15629,15923,15955G,16257

P:106,166,169,190,222,250,301,302,1130,1483,2560,2587,3381,3552,4678,5158,5503,5745,5892,5901,6162,6438,7358,7954,
7996,8238,8360,8372C,10128,10693G,11002,11142,12018,12379,12470,12677,12752,13007,13823,14038,14131T,14875,15136,
15629,15675,15953,15955G,15996,16051,16053,16060,16076,16124,16233,16257,16266

P1:169,190,222,250,301,302,1130,1483,2560,2587,3381,3552,4678,5158,5503,5745,5892,5901,6162,6438,7358,7954,7996,
8238,8360,8372C,10128,10693G,11002,11142,12018,12379,12435,12470,12677,12752,13007,13823,14038,14131T,14875,
15136,15629,15675,15953,15955G,15996,16051,16053,16060,16076,16124,16233,16257,16266

P1_GU985279:190,222,250,301,302,1130,1483,2147,2560,2587,3381,3552,4254,4295,4678,5158,5503,5745,5892,5901,6162,
6438,7358,7954,7996,8238,8360,8372C,10128,10693G,11002,11142,11742,12018,12379,12435,12470,12471,12527,12677,
12752,13007,13823,14038,14131T,14582,14875,15136,15629,15675,15953,15955G,15996,16021,16051,16053,16060,16076,
16124,16143,16233,16257,16266,16303

P1_JQ437479:169,190,222,250,301,302,1130,1483,2560,2587,3190,3304,3381,3552,4678,5158,5503,5745,5892,5901,6162,
6438,6453,7358,7954,7996,8238,8360,8372C,10128,10693G,11002,11142,12018,12379,12435,12470,12677,12752,13007,13541,
13823,14038,14131T,14875,15136,15473,15629,15675,15953,15955G,15996,16051,16060,16076,16124,16233,16257,16266

P2:106,166,173,190,222,250,301,302,1130,1483,2173,2560,2587,3381,3552,4254,4295,4678,5158,5503,5683,5745,5892,5901,
6162,6438,7358,7954,7996,8238,8360,8372C,10128,10693G,11002,11142,11470,12018,12379,12470,12677,12740,12752,13007,
13823,14038,14131T,14875,15136,15675,15953,15955G,15996,16051,16053,16060,16076,16124,16233,16249,16257,16266

R:8,106,166,250,297,301,782,895,2044,2560,2570,2981,3441,3552,3602,4192,4855,5503,5745,5892,6081,6120,6438,6477,6774,
6985,7332,7358,7361,7516,8190,8372C,8496,9007,9178,9661,9866,9980,10333,10693G,11002,11136,11394,11421,11757,
11844,12047,12236,12470,12471,12624,12677,12686,12752,12803,12902,13007,13058A,13156,13262,13460,13508,14002,
14038,14626,14827,14908,14953,15067,15136,15619,15629,15820,15902,15953,15955G,16059,16078,16123,16124,16129,
16137,16139,16233,16250,16252,16266,16303

R1:8,106,166,173,201,250,297,301,782,895,2044,2560,2570,2981,3304,3441,3552,3602,4192,4363,4855,5503,5616,5745,5892,
5937,6081,6120,6162,6438,6477,6774,6985,7332,7358,7361,7516,7933,8190,8372C,8496,8769,9007,9178,9482,9661,9866,
9980,10333,10693G,10929,11002,11136,11394,11421,11757,11844,12047,12236,12470,12471,12624,12677,12686,12752,
12803,12902,13007,13058A,13130,13156,13262,13460,13508,13719,14002,14038,14053,14459,14626,14827,14908,14953,
15067,15136,15619,15626,15629,15820,15848,15902,15953,15955G,16059,16078,16123,16124,16129,16137,16139,16233,
16250,16252,16266,16303

R1a:8,106,166,173,201,250,297,301,782,895,2044,2560,2570,2981,3304,3441,3552,3602,4192,4363,4855,5503,5616,5745,5892,
5937,6081,6120,6162,6438,6477,6774,6985,7332,7358,7361,7516,7933,8190,8372C,8496,8769,9007,9178,9482,9661,9866,
9980,10333,10693G,10929,11002,11136,11394,11421,11757,11844,12047,12236,12470,12471,12624,12677,12686,12752,
12803,12902,13007,13058A,13130,13156,13262,13460,13508,13719,14002,14038,14053,14459,14626,14827,14908,14953,
15067,15136,15581,15619,15626,15629,15820,15848,15902,15953,15955G,16059,16078,16123,16124,16129,16137,16139,
16233,16250,16252,16266,16303

R1a_FJ971085:8,106,166,173,201,250,297,301,782,895,2044,2560,2570,2981,3304,3441,3552,3602,4192,4363,4855,5503,5616,5745,5892,5937,6081,6120,6162,6438,6477,6774,6985,7332,7358,7361,7516,7933,8190,8372C,8496,8769,9007,9178,9482,9661,9866,9980,10333,10693G,10929,11002,11136,11394,11421,11757,11844,12047,12236,12470,12471,12624,12677,12686,12752,12803,12902,13007,13058A,13130,13156,13262,13460,13508,13719,14002,14038,14053,14459,14626,14827,14908,14953,15067,15136,15581,15619,15626,15629,15820,15848,15902,15953,15955G,16059,16078,16123,16124,16129,16137,16139,16233,16250,16252,16266,16303

R1a_FJ971084:8,106,166,173,201,250,297,301,782,895,2044,2560,2570,2981,3304,3441,3552,3602,4192,4363,4855,5148,5503,5616,5745,5892,5937,6081,6120,6162,6438,6477,6774,6852,6985,7332,7358,7361,7516,7933,8190,8372C,8496,8769,9007,9178,9482,9661,9866,9980,10333,10693G,10929,11002,11136,11394,11421,11757,11844,12047,12236,12470,12471,12624,12677,12686,12752,12803,12902,13007,13058A,13130,13156,13262,13460,13508,13719,14002,14038,14053,14459,14626,14827,14908,14953,15067,15136,15581,15619,15626,15629,15820,15848,15902,15953,15955G,16059,16078,16123,16124,16129,16137,16139,16233,16250,16252,16266,16303

R2:8,24,106,166,250,297,301,782,895,2044,2560,2570,2981,3441,3552,3602,4192,4630,4855,4977,5503,5745,5892,6081,6120,6438,6477,6540,6774,6985,7332,7358,7361,7516,8190,8372C,8496,8522,9007,9178,9661,9807,9866,9980,10333,10693G,10986,11002,11136,11202,11394,11421,11751,11757,11844,12047,12236,12470,12471,12624,12677,12686,12752,12803,12902,13007,13058A,13091,13156,13262,13460,13508,14002,14038,14533,14626,14827,14843,14908,14953,15067,15136,15619,15629,15820,15902,15953,15955G,15986T,16059,16078,16123,16124,16129,16137,16139,16201+A,16202,16233,16250,16252,16266,16303

T:169,13007,16257

T1'2'3'6'7:169,16257

T1:169,16052,16115,16257

T1a:169,2057+C,16052,16115,16257

T1a1:169,2057+C,11190,16052,16115,16257

T1a1_JN817315_JN817332:8,169,2057+C,11190,16052,16115,16257

T1a1_JN817335:169,173,2057+C,4114,11190,16052,16115,16257

T1a2:169,2057+C,3406,10563,12732,16052,16115,16257

T1a2a:169,2057+C,3406,7299,10563,12732,16052,16115,16257

T1a2a_JN817342:169,2057+C,3406,7299,10563,12732,15656A,16052,16115,16257

T1a2a_JN817337:169,2057+C,3406,7299,10563,12732,16052,16115,16141,16257,16304

T1a2_JN817333:169,2057+C,3406,10563,12732,13164,16052,16115,16257

T1a3:169,2057+C,9840,16052,16115,16257

T1a3_JN817314:169,756,2057+C,6388,9840,14327,16052,16115,16257

T1a3_JN817331:169,2057+C,4167,8235,8251,9840,11791,16052,16115,16135,16233,16257

T1a4:169,2057+C,4742,7322,9604,16052,16115,16121,16169,16257

T1a4_JN817317:8,169,1300,2057+C,4742,7322,9604,16052,16115,16121,16169,16257

T1a4_JN817340:169,2057+C,4742,7322,8160,9604,11956,16052,16115,16121,16169,16257

T1a5:169,2057+C,7068,12476,16052,16115,16257

T1a5_JN817338:169,353G,2025,2057+C,7068,7511,9808,12452,12476,16052,16115,16143,16166,16257

T1a5_JN817318:169,2057+C,3546A,3942,7068,12476,16052,16115,16257

T1a6:8,169,2057+C,16052,16115,16257

T1a6_JN817336:8,169,2057+C,5017,5227,5814,13125,13673,14694,14905,15527,16052,16115,16257

T1a6_JN817308:8,169,2057+C,2080,10276,16052,16060,16110,16115,16124,16257

T1a7:106,169,2057+C,16052,16115,16257

T1a7a:106,169,2057+C,8669,16052,16115,16257

T1a7a_JN817345:106,169,1842,2057+C,8669,8861,16052,16115,16257

T1a7_EU177846:106,169,2057+C,8961,15715d,16052,16078,16115,16117,16257

T1a_JN817341:169,2057+C,16018,16052,16115,16257

T1a_GU947020:169,2057+C,4159,7029,8407,11430,14429,16052,16115,16141,16149,16257

T1a_JN817303:169,2057+C,3684,4453,11028,15064,16052,16115,16248,16249,16257

T1a_JN817347:169,2057+C,9686,12902,13425,15216,16052,16115,16257,16304

T1a_JN817316:169,2057+C,2584,10603,11024,14582,15976,16052,16115,16257

T1a_JN817312:169,665,2057+C,10519,13061,13898,16052,16115,16257,16262

T1a_JN817339:169,711,2057+C,9445,10857A,16052,16115,16257,16266

T1a_EU177843:2057+C,9920,13685,16052,16115,16257

T1a_EU177844:169,2057+C,6048,15084,16052,16115,16257

T1a_JN817313:169,267T,2057+C,13880,16052,16115,16257

T1b:169,7544,16052,16115,16257

T1b1:169,7544,16024,16052,16115,16257

T1b1a:169,178,7544,14758,16024,16052,16115,16257

T1b1a_JN817351:169,178,5638,7544,14758,16024,16052,16115,16257,16293

T1b1b:169,7544,14525T,16024,16052,16115,16257

T1b1b1:169,7544,13203,14525T,16024,16052,16115,16257

T1b1b1a:169,7544,13203,14525T,16024,16052,16058,16115,16257

T1b1b1a1:169,7544,13203,14525T,16024,16052,16058,16115,16149,16257

T1b1b1a1_JN817349:169,7544,13203,14525T,16024,16052,16058,16115,16140,16149,16257

T1b1b1a1_JN817305:169,2967,7544,9433,12362,13203,14525T,16024,16052,16058,16115,16149,16257,16304

T1b1b1a2:169,7544,12471,13203,14525T,16024,16052,16058,16115,16257

T1b1b1a2a:169,179,1483,7544,10462,12470,12471,13203,14525T,16024,16052,16058,16115,16257

T1b1b1a3:169,190,498,7544,8712,12527,13203,13302,14525T,16024,16052,16058,16115,16257,16318

T1b1b1a3a:169,190,498,7544,8712,12470,12471,12527,13203,13302,14525T,16024,16052,16058,16115,16257,16318

T1b1b1a3a1:169,190,498,7544,8712,12470,12471,12527,13203,13302,14525T,16024,16052,16058,16115,16124,16257,16318

T1b1b1a3a1_KF163080:169,190,498,6477,7544,8712,12470,12471,12527,13203,13302,14525T,16024,16052,16058,16115, 16124 ,16257,16318

T1b1b1a3a2:169,190,498,3071,7544,8712,12470,12471,12527,13203,13302,14525T,16024,16052,16058,16115,16318

T1b1b1a3a2_KF163066:169,190,498,3071,7544,8190,8712,12470,12471,12527,13203,13302,14525T,16024,16052,16058,16115, 16318

T1b1b1a3a2_KF163065:169,190,498,3071,4363,7544,8712,12470,12471,12527,13203,13302,14053,14525T,16024,16052,16058, 16115,16318

T1b1b1a3a3:169,190,498,3272,7544,8712,12470,12471,12527,13203,13302,14525T,16024,16052,16058,16115,16257,16318

T1b1b1a3a3a:169,190,498,3272,7544,8712,12363,12470,12471,12527,13203,13302,14525T,16024,16052,16058,16115,16257, 16318

T1b1b1a3a3a_KF163068:169,190,498,3272,7544,8712,12363,12470,12471,12527,13203,13302,14525T,16024,16052,16058, 16115,16257,16318

T1b1b1a3a3a_KF163078:169,190,498,3272,7544,8712,12363,12470,12471,12527,13203,13302,14525T,16024,16052,16058, 16115,16123,16124,16257,16318

T1b1b1a3a3a_KF163071:169,190,498,3272,7544,8712,12363,12470,12527,13203,13302,14525T,16024,16052,16058,16115, 16257,16318

T1b1b1a3a3_KF163090:169,190,498,3272,3483,7544,8712,12470,12471,12527,13203,13302,14525T,16024,16052,16058,16115, 16257,16318

T1b1b1a3a_KF163067:169,173,190,498,7544,8712,12470,12471,12527,13203,13302,14525T,16024,16052,16058,16115,16257, 16318

T1b1b1a3a_KF163070_KF163091:169,190,498,7544,7624,8712,12470,12471,12527,13203,13262,13302,14525T,16024,16052,16 058,16115,16257,16318

T1b1b1a3a_KF163085:169,190,250,498,7544,8712,12470,12471,12527,13007,13203,13302,14525T,15953,15955G,16024,16052, 16058,16115,16257,16318

T1b1b1a3_KF163086:169,190,498,7544,8712,12527,13203,13302,14525T,16024,16052,16058,16115,16124,16257,16318

T1b1b1a3_KF163079:169,173,190,498,7544,8712,12527,13203,13302,14525T,16024,16052,16058,16115,16257,16318

T1b1b1a3_KF163064:169,190,498,3071,7544,8712,12527,13203,13302,14525T,16024,16052,16058,16059,16115,16318

T1b1b1a3_KF163077:169,190,498,1075C,6393,7544,8712,9097,12527,13203,13302,14525T,16024,16052,16058,16115,16123, 16257,16318

T1b1b1a3_KF163083:169,190,498,3272,7516,8712,12363,12527,13203,13302,14525T,16024,16052,16058,16115,16257,16318

T1b1b1c:169,7544,13203,14525T,16024,16052,16115,16150,16257

T1b1b1c1:169,7544,12470,12471,13203,14525T,16024,16052,16115,16150,16257

T1b1b1c1_KF163073:169,7544,12470,12471,13203,14525T,15158,16024,16052,16115,16257

T1b1b_JN817302:169,7544,9102,14525T,14533,14992,15846,16024,16115

T1b1c:169,7544,16024,16115,16257

T1b1c_JN817324:169,7544,8350,12229,16024,16115,16124,16257

T1b1c_DQ124399:169,3967,7544,14749A,16024,16115,16250,16257

T1b1d:169,6052,7544,14350,16024,16052,16115,16257

T1b1d_KF163061_KF163076:169,6052,7544,12470,12471,14350,16024,16052,16115,16257

T1b1_EU177842:169,7544,15421,16024,16052,16115,16257

T1b1_KF163063:169,7544,16024,16052,16115,16257,16303

T1b1_JN817334:169,4947,7544,10579,11532,13373A,15848,16024,16052,16115,16257,16303

T1b1_JN817320:3210,7544,15149,15328,16024,16052,16115,16257

T1b1_KF163084:169,7544,13397,16024,16052,16059,16070,16115,16257

T1b1_KF163092:169,7544,9503,13262,16024,16052,16115,16257

T1b_JN817327:106,169,7544,7818,14053,15948,16115

T1b_JN817348:169,4141,5898,6705,7544,9136,9658,9731,10605,13295,13984,16052,16115,16249,16257

T1c:169,16052,16115,16124,16257

T1c1:169,16052,16115,16124,16198,16257

T1c1a:169,16052,16055,16115,16124,16198,16257

T1c1a1:169,1326,11544,16052,16055,16115,16124,16141,16198,16257

T1c1a1_JN817309:169,1326,11544,13884,16052,16055,16115,16124,16141,16198,16257

T1c1a1_JN817310:169,1326,11544,16052,16055,16115,16124,16141,16198,16257,16318

T1c1a1_JN817322:106,169,1326,1876,7657,9070,11544,16018,16052,16055,16115,16124,16141,16198,16257

T1c1a1_JN817311:169,1326,10895A,11544,15464,16052,16055,16115,16124,16141,16198,16257

T1c1a_JN817346:169,10530,10797,14459,14821,16052,16055,16115,16124,16135,16198,16257

T1c1b:169,15966,16052,16115,16124,16198,16257

T1c1b_EU177847:169,4977,6934,8893,12254,15966,16052,16115,16124,16198,16257

T1c1b_JN817300_JN817301:169,3877,11475,15966,16052,16081,16115,16124,16129,16198,16202,16257

T1c1_JN817323:169,353G,1881,4349,4882,6019,8328,9722,12407,12730,15421,15958,16052,16115,16124,16198,16257

T1c1_EU177848:169,3877,8525,8864,10327,11454,12653,16052,16115,16198,16257

T1c1_JN817307:24,169,3406,5032,9677,14386,14600G,15328,15811,16052,16115,16124,16198,16257

T1c_JN817319:169,10246A,16115,16124,16257

T1c_JN817328:169,5188,8796A,12362,13733,16052,16057,16115,16124,16257

T1c_JN817325:169,10498,10926,11791,16052,16115,16124,16137,16208,16257

T1c_JN817326:169,2192,8334,11963,15100,15158,16052,16114,16115,16124,16257

T1c_KF163072:169,4588,6027,9908,10909,15462,16052,16115,16123,16124,16257

T1d:169,6237,16052,16115,16257

T1d1:169,4858,6237,16052,16115,16257

T1d1_JN817330:169,4858,6237,11457,16052,16115,16257

T1d1_JN817321:8,169,4858,6237,9514,10516,16052,16115,16250,16257

T1d1_JN817304:169,4858,6237,6333,7762,15149,16052,16115,16257

T1d_KF163081:169,6237,10349,10659,12470,16052,16115,16123,16257

T1d_JN817298:106,169,1593,3191,6237,16052,16115,16257

T1d_JN817299:169,250,4372,6237,6453,7400,11070,16052,16115,16257

T1e:8,169,16115,16257

T1e_JN817306:8,169,15941,16115,16250,16257

T1e_EU177841:8,169,9562,15398,16115,16257

T1e_KC153973_KC153975:8,169,10599,11478,12986,13859,14327,15997+XCATTAATGTAATAAAGACATAA,16076

T1f:169,12494,16052,16257

T1f_JN817343:169,3363,9638,11850,12494,14212,14611,15412,16052,16257

T1f_JN817329:169,7757,9385,12494,14045,15092,15406,16052,16257

T1g:169,300,3684,9203,12470,12471,16052,16115,16257,16262

T1g_KF163082:169,300,3684,9203,12470,12471,16052,16115,16250,16257,16262

T1g_KF163093:169,300,3684,9203,12470,12471,16052,16115,16249,16257,16262

T1c1a1_AY526085:3561,3562G,4499G,4539,5720,5756,5855,7996,11235,12160,15512,16044,16095

T2:169,6204,6744,10710,16059C,16187,16257

T2a:169,1501,5240,6204,6744,8804,9385,10710,11289,16059C,16187,16257

T2a_DQ124393:169,1501,4358,5240,6204,6744,8804,9385,10710,11289,16059C,16187,16257

T2a_DQ124396:169,1501,5240,6744,8804,9385,10710,11289,15597,16059C,16187,16257

T2b:169,5148,6204,6744,10710,16059C,16187,16257

T2b_EU177855:169,5148,6204,6744,10710,16059C,16130,16187,16257

T2b_EU177856:169,173,5148,6744,8247,10261,10710,16051,16059C,16115,16187,16257

T2b_EU177857:169,1325,1696,2601,3720,4783,5148,6204,6744,7400,10603,10710,15159,16059C,16060,16187,16250,16257,16303

T2c:169,2087,3192,6204,6744,9989,10710,11736,14581,16059C,16187,16257

T2c_EU177860:169,2087,3192,6204,6744,9482,9989,10710,11736,13398,14581,15196,16059C,16187,16257

T2c_EU177861:2087,2749,3192,6204,6316,6744,8986G,9989,10710,11736,14581,16059C,16187,16257

T2d:169,6204,6744,10710,16059C,16076,16187,16257

T2d_EU177851:169,1461,2560,6204,6744,10710,11103,12653,15798,15987,16059C,16076,16187,16257

T2d_EU177852:169,6204,6744,10710,16059C,16069,16076,16123,16187,16257

T2_EU177853:169,170,3942,5158T,5763,6204,6744,10352,10710,13753,16052,16059C,16143,16187,16257

T2_EU177854:169,1653,6204,6744,9502,10710,14404,16059C,16064,16187,16249,16257,16303

T2_DQ124383:169,174,6204,6744,8516,10710,11370,15558,16010C,16059C,16124,16187,16257

T2_HQ025805:169,3561,3562G,4108,4327,5855,6204,6744,7960,10710,12272,14418,16059C,16187,16257

T2_EU177849:169,4955,5634C,6204,6744,10710,16059C,16133,16187,16257

T2_EU177850:169,6204,6744,7852,10710,13203,16059C,16060,16127,16187,16257

T2_EU177858:119,120,166,169,3351,3852,6204,6405,6744,8477,9310,10662,10710,11832,12294,12506,12767,13482,15885d,16059C,16082,16187,16233,16257

T2_EU177859:169,1555,4977,6204,6207,6744,8862,10710,16059C,16141,16187,16257

T2_AY676856:169,5603,6204,6744,10710,12545,15419,16059C,16079,16187,16257,16262

T3:169

T3a:169,12160

T3a1:169,12160,16044,16095

T3a1a:169,12160,15512,16044,16095

T3a1a_DQ124418:12160,15512,16044,16095

T3a1_DQ124417:9780T,12160,16044,16095

T3a2:169,8712,9482,12160,16051,16121

T3b:169,14065

T3b_DQ124385:169,3336,3852A,4771,11736,12225,14065,16129

T3b_DQ124416:169,6575,6994C,14065

T3c:169,12730

T3c_DQ124409:169,1483,8171,9022,10387,12730

T3c_DQ124410:8168A,10890,12730,14134,16250

T3d:169,13901

T3d1:169,5158,13691,13901

T3d1_AY676861:169,5158,13691,13901,16143

T3d1_AY676871:169,1134,5158,13691,13901,16304

T3d_GU947019:166,169,537,567,8927,13901

T3e:169,10884

T3e_AF492351:169,190,10884,12236,15581,16071,16143

T3e_KC153974:169,5226,8832,10884,11514,13487,16140

T3f:169,15987d,16059+A,16074A,16078

T3f1:169,9731,15987d,16059+A,16076A,16078

T3f1a:169,4333,9731,15987d,16059+A,16076A,16078

T3f1a_GU947008:169,4333,5044,9731,15987d,16059+A,16076A,16078

T3f1a_GU947014:169,4333,5226,9731,13784T,15987d,16059+A,16076A,16078

T3f1_GU947012_GU947015:169,6327,9731,12629,13280,15987d,16059+A,16076A,16078,16233

T3f2:169,190,1865,2632T,3810,5044,7241,9064,9238,15987d,16059+A,16070,16076A,16078

T3f2_GU947007:169,190,1865,2632T,3810,5044,7241,9064,9238,14218,14233,15987d,16059+A,16070,16076A,16078

T3f3:169,10462,15987d,16059+A,16076A,16078,16149

T3f3_GU947010:169,532+G,10462,15987d,16059+A,16076A,16078,16149

T3f3_GU947018:169,10462,14218,14233,15987d,16059+A,16076A,16078,16149

T3f4:5128,15196,15987d,16059+A,16076A,16078

T3g:169,5718,13520

T3g_GU947021:169,2055,4756C,4757C,5604,5718,7011,12025,12178,13091A,13520,16138,16225,16228C

T3g_AY676864:169,173,5146,5718,7125,8407,10602,12038,13520,15936

T3h:169,10743

T3h_DQ124390:169,816,2147,3304,3873,8927,10743,13403

T3h_DQ124411:106,3546,10743

T3i:169,1066+A,9449,14080,14563,15217

T3i_EU177822:169,1066+A,9449,14080,14524,14563,15217,15421

T3i_EU177823:169,1066+A,2222,9449,14080,14230,14563,15104,15217,15388,16024,16202

T3j:169,13526

T3j_EU177829:169,1292,6501,6948,7801,13526,16135,16252

T3j_EU177830:169,313A,8882,10872,13526,16044

T3k:169,12910

T3k1:169,10891,12910,15912,16057

T3k1_EU177837:169,3088,3798,9098,10891,12910,15912,16057

T3k1_DQ124376:169,1134,2571,6621,9731,10891,12910,15912,16057,16114,16169

T3k_DQ124386:106,169,3729,12910,14582,16044,16095

T3l:169,3343,7624,12025,12175,14038,15966

T3l_EU177834:169,3343,7624,12025,12175,14038,15966

T3n:169,16121

T3n1:169,5946,10085,13431,16121,16230

T3n1_DQ124391:169,5946,7781,10085,13431,16121,16230

T3f1a_DQ124397:169,3858,5946,7781,10085,13431,16121,16230

T3n_DQ124373:169,16115,16121

T3n_AY676857:169,190,4393A,5850,6636A,16055,16121

T3o:169,16124

T3o1:169,7778,11037,12686,16124

T3o1a:8,169,7778,11037,12686,16053,16124

T3o1a_DQ124388_DQ124394_DQ124398:8,169,7778,11037,12686,16053,16124,16249

T3o1_DQ124380:169,7778,8288,11037,12686,13630,16124

T3o1_DQ124382:169,7778,8668G,11037,12160,12686,13031,16124

T3o1_DQ124402:169,5683,7375G,7778,11037,12686,15760,16124

T3o_DQ124374:8,169,1195,3720,5222A,10993,15331,16069,16124

T3o_AY676873:169,8918,12803,16024,16124,16137

T3p:169,16059

T3p_EU177825:169,13496,16059

T3p_EU177824:169,5104,14596,16059,16060,16115

T3q:169,16060

T3q1:169,16060,16233

T3q1_EU177826:169,2080,2702+C,6135,8654,9517,12211,14466,16060,16231,16233

T3q1_EU177827:11674,12161,13697,13745,16060,16076,16142,16233,16252

T3q2:169,16060,16121

T3q2_DQ124384:169,9686,16060,16121

T3q2_EU177828:106,169,1554,4339,4387,5143,14293,16060,16121,16129,16249

T3r1:4903,16044

T3r1_AY676868_AY676869:173,811,3877,4903,16044,16233,16234

T3r1_AY676872:4903,10578,12060,16044

T3r2:1092

T3r2_EU177817:119,1092,4096,9070,16143

T3r2_EU177818:1092,5544+A,6570,11751,13166,13403,13832,13856,15838,16044

T3r2_AY676858:1092,3315,9731,12023,13772,15275,16064

T3r3:12707,16044

T3r3_EU177819:3729,12707,16044,16304

T3r3_EU177820:173,2029,4002,12707,15356,15962,16044,16124,16230

T3r4:12732

T3r4_DQ124408:7213,12732,15558

T3r4_DQ124413_AY676866:3345,8712,12167,12732,14908,16249

T3r4_EU177821:169,11250,12732,12881

T3r5:173

T3r5_DQ124415:173,1493,6932,9731,13175,16303

T3o1a_DQ124407:173,1493,6932,9731,13175,15597,16303

T3r5_AY676865:173,5140,7137,8384,13556,15963,16059

T3r6:353G

T3r6_GQ129208:353G,9136,12160

T3r6_KC153977:353G,10686,14418,16064,16249

T3r_AY676862:222,9076,9118C,10349,15742,16002,16143

T3r_GQ129207:166,4744,16133

T3r_HM045018:3189

T3r_AY676863:641,1483,11085,13312

T3r_EU177815:3304,6822,9259,13142,15879

T3r_EU177816:163,565,10698,11028,12452,16024

T3s:8,169

T3s_EU177836:8,169,514,4822,15094,16068,16111

T3s_JQ967333:8,169,13257,15989+GGACATAACATTAATGTAATAAAGACATAACATTAATGTAATAA,16078,16137

T3_EU177832:169,16135

T3_KF926377:169,4060,8063A,8065A,8066A,8069,13734,16053,16063,16131

T3_AY676859:169,687,14564,16114

T3_KC153972:169,177,16051,16052

T3_DQ124406:169,2127,3786,16250

T3_DQ124414:169,5473C,12236,15209,16071

T3_DQ124404:35,169,7573,7612,9178,14128,16112

T3_DQ124405:169,737,1522,5074,6082,11531C,15941

T3_AY676860:169,5047,7630,7920,9971,10462,15157,15494,15963,16149

T3_AY676855:169,6732,14849,15581,16207

T3_KC153976:169,236,3282,6336,9604

T3_DQ124387_DQ124395:169,3381,12175-12177d,14747,16088

T3_EU177831:169,1292T,6019,8900,13472,14180

T3_EU177835:169,13880,14155

T3_EU177838:169,532+G,1862+A,6690,7194,14368,16303

T3_EU177839:169,8242,11187,11802,16110,16304

T4:169,12160,15512,16044,16095,16304

T4a:169,11176,12160,15512,16044,16095,16304

T4a_DQ124372:169,6690,7705,8212,11176,12160,12494,14909C,15512,16044,16095,16304

T4a_DQ124375:169,9121,11176,12160,13233,15512,16044,16076,16095,16304

T4_DQ124377:169,7867,12160,15512,16044,16051,16059,16095,16106,16166,16304

T4_DQ124392:169,11010,12160,15512,16044,16095,16304

T4_DQ124400:169,5378,10469,12160,13511,14059,15512,16044,16095,16304

T4_DQ124401:169,353G,5599,12160,15512,16044,16095,16304

T4_DQ124412:169,12160,15512,16044,16095,16118,16304

T5:163,169,4254,12983,13007,16257

T5a:163,4254,8026,9091,9234,10881,12983,13007,15884,16199,16257

T5a_EU177863:163,4254,8026,9091,9234,10881,12782,12983,13007,15884,16199,16257

T5b:163,169,4254,9896A,12983,13007,16257

T5b_EU177864:163,169,4254,7660,9896A,12983,13007,16257

T5b_EU177865:163,169,204,1292T,4254,8047,9896A,12983,13007,16139,16257

T6:106,169,7933,11901,12925T,13376,14065,16111,16257

T7:169,3711T,8640,9305,16076,16257

I:8,106,166,206,233,234,249,250,297,301,519,739,763,818,1160,1476,1494,1679,1826,1862A,1871,2018,2101,2560,2955,2979,2981,2990,2991,2992,3053,3073,3138,3147,3243,3327,3337,3381,3441,3537,3552,3602,3795,3831,3876,3933,3978,3987,4002,4330,4444,4564,4732,4735,4771,4873,4939,5287,5503,5533,5616,5745,5785,5892,5919,6000,6117T,6237,6342,6369,6381,6438,6462,6729,6774,6883,6924,7306,7332,7358,7360,7363,7516,7832,7853,8047,8170,8190,8212,8287,8310,8372C,8468,8496,8505,8573,8751,8986,9007,9040,9070,9247,9583,9604,9769,9893,9930C,9980,10041,10068,10139,10155,10270,10324,10333,10447,10592,10623,10693G,10851,11002,11037,11070,11136,11202,11268,11331,11409,11421,11805,11844,12137,12180,12236,12379,12435,12470,12471,12515,12624,12674,12677,12686,12752,12803,12902,12925T,12926,13007,13058A,13100,13106,13277,13382,13435,13439,13556,13566,13586,13679,13691,13694,13884,13908A,13911,14038,14068,14122,14131,14140,14257,14317,14413,14418,14505,14608,14827,14860,14899,15107,15136,15148,15289,15310,15328,15565,15581,15595,15607,15619,15629,15631,15743,15753,15820,15953,15955G,15961,15996,16024,16051,16059,16060,16076,16084,16104,16111,16115,16118,16119,16121,16123,16124,16132,16139,16140,16145d,16149,16198,16231,16249,16250,16302,16303

I1:8,106,166,173,206,233,234,249,250,297,301,519,724,739,763,818,1160,1476,1494,1679,1826,1862A,1871,2018,2101,2560,2577,2636,2955,2979,2981,2990,2991,2992,3053,3073,3138,3147,3243,3327,3337,3381,3441,3537,3552,3602,3795,3831,3876,3933,3978,3987,4002,4330,4444,4564,4732,4735,4771,4873,4939,4990A,5287,5503,5533,5616,5745,5785,5892,5919,6000,6117T,6237,6342,6369,6381,6438,6462,6729,6774,6883,6924,7306,7332,7358,7360,7363,7516,7832,7853,8047,8170,8190,8196,8212,8287,8310,8372C,8468,8496,8505,8516,8573,8751,8986,9007,9040,9070,9247,9304,9482,9583,9604,9769,9893,9932C,9980,10041,10068,10073,10139,10155,10270,10324,10333,10447,10592,10623,10693G,10851,11002,11037,11070,11136,11202,11268,11331,11409,11421,11805,11844,12137,12180,12236,12379,12435,12470,12471,12515,12624,12674,12677,12686,12752,12803,12902,12925T,12926,13007,13058A,13100,13103A,13106,13277,13373,13382,13435,13439,13556,13566,13586,13630,13679,13691,13694,13884,13912A,13911,14038,14068,14122,14131,14140,14257,14317,14413,14418,14505,14608,14827,14860,14899,15107,15136,15148,15289,15310,15328,15565,15581,15595,15607,15619,15629,15631,15743,15753,15820,15953,15955G,15961,15996,16024,16051,16059,16060,16076,16084,16104,16111,16115,16118,16119,16121,16123,16124,16132,16139,16140,16145d,16149,16198,16231,16249,16250,16302,16303

I1_AY126697:8,106,166,173,206,233,234,249,250,297,301,519,724,739,763,818,1160,1476,1494,1679,1826,1862A,1871,2018,2101,2560,2577,2636,2955,2979,2981,2990,2991,2992,3053,3073,3138,3147,3243,3327,3337,3357,3381,3441,3537,3552,3602,3795,3831,3876,3933,3978,3987,4002,4330,4444,4564,4732,4735,4771,4873,4939,4990A,5287,5503,5533,5616,5745,5785,5892,5919,6000,6117T,6237,6342,6369,6381,6438,6462,6729,6774,6883,6924,7306,7332,7358,7360,7363,7516,7832,7853,7894,8047,8170,8190,8196,8212,8287,8310,8372C,8468,8496,8505,8516,8573,8751,8986,9007,9040,9070,9247,9304,9482,9583,9604,9769,9893,9932C,9980,10041,10068,10073,10139,10155,10270,10324,10333,10447,10592,10623,10693G,10851,11002,11037,11070,11136,11202,11268,11331,11409,11421,11805,11844,12137,12180,12236,12379,12435,12470,12471,12515,12624, 12674,12677,12686,12752,12803,12902,12925T,12926,13007,13058A,13100,13103A,13106,13277,13373,13382,13435,13439,13556,13566,13586,13630,13679,13691,13694,13884,13912A,13911,14038,14068,14122,14131,14140,14257,14317,14413,14418,14505,14608,14827,14860,14899,15107,15136,15148,15289,15310,15328,15565,15581,15589,15595,15607,15619,15629,15631,15743,15753,15820,15953,15955G,15961,15996,16024,16051,16059,16060,16076,16084,16104,16111,16115,16118,16119,16121,16123,16124,16132,16139,16140,16145d,16149,16198,16231,16249,16250,16302,16303

I1_EU177868:8,106,166,173,206,233,234,249,250,297,301,519,724,739,763,818,1160,1476,1494,1679,1826,1862A,1871,2018,
2101,2323A,2560,2577,2636,2955,2979,2981,2990,2991,2992,3053,3073,3138,3147,3243,3327,3337,3381,3441,3537,3552,
3602,3795,3831,3876,3933,3978,3987,4002,4330,4444,4564,4732,4735,4771,4873,4939,4990A,5287,5503,5533,5616,5745,
5785,5892,5919,6000,6117T,6237,6342,6369,6381,6438,6462,6729,6774,6883,6924,7306,7332,7358,7360,7363,7516,7832,7853,
8047,8170,8190,8196,8212,8287,8310,8372C,8468,8496,8505,8516,8573,8751,8986,9007,9040,9070,9247,9304,9482,9583,
9604,9769,9893,9932C,9980,10041,10068,10073,10139,10155,10270,10324,10333,10447,10592,10623,10693G,10851,11002,
11037,11070,11136,11202,11268,11331,11409,11421,11805,11844,12137,12180,12236,12379,12435,12470,12471,12515,
12624,12674,12677,12686,12752,12803,12902,12925T,12926,13007,13058A,13100,13103A,13106,13277,13373,13382,13435,
13439,13556,13566,13586,13630,13679,13691,13694,13884,13912A,13911,14038,14068,14122,14131,14140,14257,14317,
14413,14418,14505,14608,14827,14860,14899,15107,15136,15148,15289,15310,15328,15565,15581,15595,15607,15619,
15629,15631,15743,15753,15820,15953,15955G,15961,15996,16024,16051,16059,16060,16076,16084,16104,16111,16115,
16118.16119.16121.16123.16124.16132.16139.16140.16145d.16149.16198.16231.16249.16250.16302.16303

I1_FJ971088:8,106,166,173,206,233,234,249,250,297,301,519,724,739,763,818,1160,1476,1494,1679,1826,1862A,1871,2018,
2101,2560,2577,2636,2955,2979,2981,2990,2991,2992,3053,3073,3138,3147,3243,3312,3327,3337,3381,3441,3537,3552,3602,
3795,3831,3876,3933,3978,3987,4002,4330,4444,4564,4732,4735,4771,4873,4939,4990A,5287,5503,5533,5616,5745,5785,
5892,5919,6000,6117T,6237,6342,6369,6381,6438,6462,6729,6774,6883,6924,7306,7332,7358,7360,7363,7516,7832,7853,8047,
8170,8190,8196,8212,8287,8310,8372C,8468,8496,8505,8516,8573,8751,8986,9007,9040,9070,9247,9304,9482,9583,9604,
9769,9893,9932C,9980,10041,10068,10073,10139,10155,10270,10324,10333,10447,10592,10623,10693G,10851,11002,11037,
11070,11136,11202,11268,11331,11409,11421,11805,11844,12137,12139,12180,12236,12379,12435,12470,12471,12515,
12624,12674,12677,12686,12752,12803,12902,12925T,12926,13007,13058A,13100,13103A,13106,13277,13373,13382,13435,
13439,13556,13566,13586,13630,13679,13691,13694,13884,13912A,13911,14038,14068,14122,14131,14140,14257,14317,
14413,14418,14505,14608,14827,14860,14899,15107,15136,15148,15159,15289,15310,15328,15565,15581,15595,15607,
15619,15629,15631,15743,15753,15820,15953,15955G,15961,15996,16024,16051,16059,16060,16076,16084,16104,16111,
16115,16118,16119,16121,16123,16124,16132,16139,16140,16145d,16149,16198,16231,16249,16250,16302,16303

I2:8,39,106,166,206,233,234,249,250,297,301,519,739,763,818,1160,1476,1494,1679,1826,1862A,1871,2018,2101,2560,2955,
2979,2981,2990,2991,2992,3053,3073,3138,3147,3243,3318,3327,3337,3381,3441,3537,3552,3602,3795,3831,3876,3933,3978,
3987,4002,4330,4444,4564,4732,4735,4771,4873,4939,5287,5503,5533,5616,5745,5785,5892,5919,6000,6117T,6237,6342,6369,
6381,6438,6462,6729,6774,6883,6924,7306,7332,7358,7360,7363,7516,7832,7853,8047,8170,8190,8212,8287,8310,8372C,
8468,8496,8505,8573,8751,8986,9007,9040,9070,9247,9583,9604,9769,9893,9932C,9980,10041,10068,10139,10155,10270,
10324,10333,10447,10592,10623,10693G,10851,10890,10935,11002,11037,11070,11136,11202,11268,11331,11409,11421,
11805,11844,12137,12180,12236,12379,12435,12470,12471,12515,12624,12674,12677,12686,12752,12803,12902,12925T,
12926,13007,13058A,13100,13106,13277,13382,13435,13439,13556,13566,13586,13679,13691,13694,13884,13912A,13911,
14038,14068,14122,14131,14140,14257,14317,14371,14413,14418,14505,14608,14827,14860,14899,15107,15136,15148,
15289,15310,15328,15565,15581,15595,15607,15619,15629,15631,15743,15753,15820,15953,15955G,15961,15996,16024,
16051,16059,16060,16076,16084,16104,16111,16115,16118,16119,16121,16123,16124,16132,16139,16140,16143,16145d,
16149,16198,16231,16234,16249,16250,16302,16303

I2_AF492350:8,39,106,166,206,233,234,249,250,297,301,519,739,763,818,1160,1476,1494,1679,1826,1862A,1871,2018,2101,
2560,2955,2979,2981,2990,2991,2992,3053,3073,3138,3147,3243,3318,3327,3337,3381,3441,3537,3552,3602,3795,3831,3876,
3933,3978,3987,4002,4330,4444,4564,4732,4735,4771,4873,4939,5287,5503,5533,5616,5745,5785,5892,5919,6000,6117T,6237,
6342,6369,6381,6438,6462,6729,6774,6883,6924,7306,7332,7358,7360,7363,7516,7832,7853,7948,8047,8170,8190,8212,8287,
8310,8372C,8468,8496,8505,8573,8751,8986,9007,9040,9070,9247,9583,9604,9769,9893,9932C,9980,10041,10068,10139,
10155,10270,10324,10333,10447,10592,10623,10693G,10851,10890,10935,11002,11037,11070,11136,11202,11268,11331,
11409,11421,11805,11844,12137,12180,12236,12379,12435,12470,12471,12515,12624,12674,12677,12686,12752,12803,
12902,12925T,12926,13007,13058A,13100,13106,13277,13382,13435,13439,13556,13566,13586,13679,13691,13694,13884,
13912A,13911,14038,14068,14122,14131,14140,14257,14317,14371,14413,14418,14505,14608,14827,14860,14899,15107,
15136,15148,15289,15310,15328,15565,15581,15595,15607,15619,15629,15631,15743,15753,15820,15953,15955G,15961,
15996,16024,16051,16059,16060,16076,16084,16104,16111,16115,16118,16119,16121,16123,16124,16132,16139,16140,
16143.16145d.16149.16198.16231.16234.16249.16250.16302.16303

I2_EU177870:8,39,106,166,206,233,234,249,250,297,301,519,739,763,818,1160,1476,1494,1679,1826,1862A,1871,2018,2101,
2560,2955,2979,2981,2990,2991,2992,3053,3073,3138,3147,3243,3318,3327,3337,3381,3441,3537,3552,3602,3795,3831,3876,
3933,3978,3987,4002,4312,4330,4444,4564,4732,4735,4771,4873,4939,5287,5503,5533,5616,5745,5785,5892,5919,6000,6117T,
6237,6342,6369,6381,6438,6462,6729,6774,6883,6924,7306,7332,7358,7360,7363,7501,7516,7832,7853,8047,8170,8190,8212,
8287,8310,8372C,8468,8496,8505,8573,8751,8986,9007,9040,9070,9247,9583,9604,9769,9893,9932C,9980,10041,10068,10139,
10155,10270,10324,10333,10447,10592,10623,10690,10693G,10851,10890,10935,11002,11037,11070,11136,11202,11268,
11331,11409,11421,11805,11844,12137,12180,12236,12341,12379,12435,12470,12471,12515,12624,12674,12677,12686,
12752,12803,12902,12925T,12926,13007,13058A,13100,13106,13277,13382,13435,13439,13556,13566,13586,13679,13691,
13694,13884,13912A,13911,14038,14068,14122,14131,14140,14257,14317,14371,14413,14418,14505,14608,14827,14860,
14899,15107,15136,15148,15289,15310,15328,15565,15581,15595,15607,15619,15629,15631,15743,15753,15820,15953,
15955G,15961,15996,16024,16051,16059,16060,16076,16084,16104,16111,16115,16118,16119,16121,16123,16124,16132,
16139,16140,16143,16145d,16149,16198,16231,16234,16249,16250,16302,16303

I2_EU177869:8,39,106,166,173,206,233,234,249,250,297,301,519,739,763,818,1160,1476,1494,1679,1826,1862A,1871,2018,210
1,2560,2955,2979,2981,2990,2991,2992,3053,3073,3138,3147,3243,3318,3327,3337,3381,3441,3537,3552,3602,3795,3831,387
6,3933,3978,3987,4002,4330,4444,4564,4732,4735,4771,4873,4939,5275T,5287,5503,5533,5616,5745,5785,5892,5919,6000,611
7T,6237,6342,6369,6381,6438,6462,6498,6729,6774,6883,6924,7306,7332,7358,7360,7363,7516,7832,7853,8047,8170,8190,821
2,8287,8310,8372C,8468,8496,8505,8573,8751,8986,9007,9040,9070,9247,9583,9604,9769,9893,9932C,9980,10041,10068,1013
9,10155,10270,10324,10333,10447,10592,10623,10693G,10851,10890,10935,11002,11037,11070,11136,11202,11268,11331,11
409,11421,11805,11844,12137,12180,12236,12379,12435,12470,12471,12515,12624,12674,12677,12686,12752,12803,12902,1
2925T,12926,13007,13058A,13100,13106,13277,13382,13435,13439,13556,13566,13586,13679,13691,13694,13884,13912A,139
11,14038,14068,14122,14131,14140,14234,14257,14317,14371,14413,14418,14505,14608,14827,14833,14860,14899,15107,15
136,15148,15289,15310,15328,15565,15581,15595,15607,15619,15629,15631,15743,15753,15820,15953,15955G,15961,15996,
16024,16051,16052A,16059,16060,16076,16084,16104,16111,16115,16118,16119,16121,16123,16124,16132,16139,16140,1614
3,16145d,16149,16198,16231,16234,16249,16250,16302,16303

Table S2: List of variants from animal sequences available at NCBI, aligned to ARS-UCD1.2_M Ref and used by MitoToolsPy to determine haplogroup.

cattleRef_V00654:222d,364C,589d,2538C,3345,3387,3541,4321,8190,8712,9684G,12167,13312A,15637

AY126697:8,106,166,173,206,233,234,249,250,297,301,364+CC,364C,519,724,739,763,818,1160,1476,1494,1602d,1679,1826,
1862A,1871,2018,2101,2560,2577,2636,2955,2979,2981,2990,2991,2992,3053,3073,3138,3147,3243,3327,3337,3345,3357,
3381 ,3387,3441,3537,3541,3552,3602,3795,3831,3876,3933,3978,3987,4002,4321,4330,4444,4564,4732,4735,4771,4873,4939,
4990A,5287,5503,5533,5616,5745,5785,5892,5919,6000,6117T,6237,6342,6369,6381,6438,6462,6729,6774,6883,6924,7306,
7332,7358,7360,7363,7516,7832,7853,7894,8047,8170,8196,8212,8287,8310,8372C,8468,8496,8505,8516,8573,8712,8751,
8986,9007 ,9040,9070,9247,9304,9482,9583,9604,9769,9893,9932C,9980,10041,10068,10073,10139,10155,10270,10324,10333,
10447,10592,10623,10693G,10851,11002,11037,11070,11136,11202,11268,11331,11409,11421,11805,11844,12137,12167,
12180,12236,12379,12435,12470,12471,12515,12624,12674,12677,12686,12752,12803,12902,12925T,12926,13007,13058A,
13100,13103A,13106,13277,13373,13382,13435,13439,13556,13566,13586,13630,13679,13691,13694,13884,13910A,13911,
14038,14068,14122,14131,14140,14257,14317,14413,14418,14505,14608,14827,14860,14899,15107,15136,15148,15289,
15310,15328,15565 ,15581,15589,15595,15607,15619,15629,15631,15637,15743,15753,15820,15953,15955G,15961,15996,
16024,16051,16059,16060,16076,16084,16086,16104,16111,16115,16118,16119,16121,16123,16124,16132,16139,16140,
16145d,16149,16198, 16202+A,16231,16249,16250,16302,16303

AY526085:222d,364C,589d,3345,3387,3541,3561,3562G,4321,4499G,4539,5720,5756,5855,7996,8190,8712,11235,12160,
12167, 15512,15637,16044,16095

AY676855:169,364C,1602d,3345,3387,3541,4321,6732,8190,8712,12167,14849,15581,15637,16207

AY676856:169,222d,364C,3345,3387,3541,4321,5603,6204,6744,8190,8712,10710,12167,12545,15419,15637,16059C,16079,
16187,16257,16262

AY676857:169,190,364C,364+C,1602d,3345,3387,3541,4321,4393A,5850,6636A,8190,8712,12167,15637,16055,16121

AY676858:222d,364C,1092,1602+AA,3315,3345,3387,3541,4321,8190,8712,9731,12023,12167,13772,15275,15637,16064

AY676859:169,687,3345,3387,3541,4321,8190,8712,12167,14564,15637,16114

AY676860:169,222+C,364C,1602d,3345,3387,3541,4321,5047,7630,7920,8190,8712,9971,10462,12167,15157,15494,15637,
15963,16149

AY676861:169,364+C,364C,3345,3387,3541,4321,5158,8190,8712,12167,13691,13901,15637,16143

AY676862:224d,3345,3387,3541,4321,8190,8712,9076,9118C,10349,12167,15637,15742,16002,16087,16143

AY676863:222d,641,1483,1602d,3345,3387,3541,4321,8190,8712,11085,12167,15637

AY676864:169,173,222d,364C,364+CC,1602d,3345,3387,3541,4321,5146,5718,7125,8190,8407,8712,10602,12038,12167,13520,
15637,15936

AY676865:173,222d,365d,1602d,3345,3387,3541,4321,5140,7137,8190,8384,8712,12167,13556,15637,15963,16059

AY676868:173,222d,811,3345,3387,3541,3877,4321,4903,8190,8712,12167,15637,16044,16233,16234

AY676871:169,364C,1134,3345,3387,3541,4321,5158,8190,8712,12167,13691,13901,15637,16304

AY676872:222+C,364C,3345,3387,3541,4321,4903,8190,8712,10578,12060,12167,15637,16044

AY676873:169,365d,3345,3387,3541,4321,8190,8712,8918,12167,12803,15637,16024,16124,16137

DQ124372:169,364C,3345,3387,3541,4321,6690,7705,8190,8212,8712,11176,12160,12167,12494,14909C,15512,15637,16044,
16095,16304

DQ124373:169,364C,3345,3387,3541,4321,8190,8712,12167,15637,16115,16121

DQ124374:8,169,364C,1195,3345,3387,3541,3720,4321,5222A,8190,8712,10993,12167,15331,15637,16069,16124

DQ124375:169,364C,3345,3387,3541,4321,8190,8712,9121,11176,12160,12167,13233,15512,15637,16044,16076,16095,16304

DQ124376:169,364C,1134,2571,3345,3387,3541,4321,6621,8190,8712,9731,10891,12167,12910,15637,15912,16057,16114,
16169

DQ124377:169,364C,3345,3387,3541,4321,7867,8190,8712,12160,12167,15512,15637,16044,16051,16059,16095,16106,16166,
16304

DQ124380:169,364C,3345,3387,3541,4321,7778,8190,8288,8712,11037,12167,12686,13630,15637,16124

DQ124382:169,364C,3345,3387,3541,4321,7778,8190,8668G,8712,11037,12160,12167,12686Y,13031,15637,16124

DQ124383:169,174,364C,3345,3387,3541,4321,6204,6744,8190,8516,8712,10710,11370,12167,15558,15637,16010C,16059C,
16124,16187,16257

DQ124384:169,364C,3345,3387,3541,4321,8190,8712,9686,12167,15637,16060,16121

DQ124385:169,364C,3336,3345,3387,3541,3852A,4321,4771,8190,8712,11736,12167,12225,14065,15637,16129

DQ124386:106,169,364C,3345,3387,3541,3729,4321,8190,8712,12167,12910,14582,15637,16044,16095

DQ124387:169,364C,3345,3381,3387,3541,4321,8190,8712,12167,12173-12175d,14747,15637,16088

DQ124388:8,169,364C,3345,3387,3541,4321,7778,8190,8712,11037,12167,12686,15637,16053,16124,16249

DQ124390:169,364C,816,2147,3304,3345,3387,3541,3873,4321,8190,8712,8927,10743,12167,13403,15637

DQ124391:169,364C,3345,3387,3541,4321,5946,7781,8190,8712,10085,12167,13431,15637,16121,16230

DQ124392:169,364C,3345,3387,3541,4321,8190,8712,11010,12160,12167,15512,15637,16044,16095,16304

DQ124393:169,364C,1501,3345,3387,3541,4321,4358,5240,6204,6744,8190,8712,8804,9385,10710,11289,12167,15637,16059C,16187,16257

DQ124396:169,364C,1501,3345,3387,3541,4321,5240,6744,8190,8712,8804,9385,10710,11289,12167,15597,15637,16059C,16187,16257

DQ124397:169,364C,3345,3387,3541,3858,4321,5946,7781,8190,8712,10085,12167,13431,15637,16121,16230

DQ124399:169,364C,3345,3387,3541,3967,4321,7544,8190,8712,12167,14749A,15637,16024,16115,16250,16257

DQ124400:169,364C,3345,3387,3541,4321,5378,8190,8712,10469,12160,12167,13511,14059,15512,15637,16044,16095,16304

DQ124401:169,353G,364C,3345,3387,3541,4321,5599,8190,8712,12160,12167,15512,15637,16044,16095,16304

DQ124402:169,364C,3345,3387,3541,4321,5683,7375G,7778,8190,8712,11037,12167,12686,12940Y,15637,15760,16124

DQ124404:35,169,364C,3345,3387,3541,4321,7573,7612,8190,8712,9178,12167,14128,15637,16112

DQ124405:169,364C,737,1522,3345,3387,3541,4321,5074,6082,8190,8712,11531C,12167,15637,15941

DQ124406:169,364C,2127,3345,3387,3541,3786,4321,8190,8712,12167,15637,16250

DQ124407:173,364C,1493,3345,3387,3541,4321,6932,8190,8712,9731,12167,13175,15597,15637,16303

DQ124408:364C,3345,3387,3541,4321,7213,8190,8712,12167,12732,15558,15637

DQ124409:169,364C,1483,3345,3387,3541,4321,8171,8190,8712,9022,10387,12167,12730,15637

DQ124410:364C,3345,3387,3541,4321,8168A,8190,8712,10890,12167,12730,14134,15637,16250

DQ124411:106,364C,3345,3387,3541,3546,4321,8190,8712,10743,12167,15637

DQ124412:169,364C,3345,3387,3541,4321,8190,8712,12160,12167,15512,15637,16044,16095,16118,16304

DQ124413:364C,3387,3541,4321,8190,12732,14908,16249

DQ124414:169,364C,3345,3387,3541,4321,5473C,8190,8712,12167,12236,15209,15637,16071

DQ124415:173,364C,1493,3345,3387,3541,4321,6932,8190,8712,9731,12167,13175,15637,16303

DQ124416:169,364C,3345,3387,3541,4321,6575,6996C,8190,8712,12167,14065,15637

DQ124417:364C,3345,3387,3541,4321,8190,8712,9780T,12160,12167,15637,16044,16095

DQ124418:364C,3345,3387,3541,4321,8190,8712,12160,12167,15512,15637,16044,16095

EU177815:363+C,3304,3345,3387,3541,4321,6822,8190,8712,9259,12167,13142,15637,15879

EU177816:163,363+C,565,3345,3387,3541,4321,8190,8712,10698,11028,12167,12452,15637,16024

EU177817:119,363+C,1092,3345,3387,3541,4096,4321,8190,8712,9070,12167,15637,16143

EU177818:222+C,363+C,1092,1602+A,3345,3387,3541,4321,5544+A,6570,8190,8712,11751,12167,13166,13403,13832,13856,15637,15838,16044

EU177819:364C,3345,3387,3541,3729,4321,8190,8712,12167,12707,15637,16044,16304

EU177820:173,222d,364C,2029,3345,3387,3541,4002,4321,8190,8712,12167,12707,15356,15637,15962,16044,16124,16230

EU177821:169,364C,1602d,3345,3387,3541,4321,8190,8712,11250,12167,12732,12881,15637

EU177822:169,222d,363+C,1068+A,1602d,3345,3387,3541,4321,8190,8712,9449,12167,14080,14524,14563,15217,15421,15637

EU177823:169,222d,363+C,1068+A,1602d,2222,3345,3387,3541,4321,8190,8712,9449,12167,14080,14230,14563,15104,15217,15388,15637,16024,16202

EU177824:169,363+C,1602d,3345,3387,3541,4321,5104,8190,8712,12167,14596,15637,16059,16060,16115

EU177825:169,364C,1602d,3345,3387,3541,4321,8190,8712,12167,13496,15637,16059

EU177826:169,364C,2080,2702+C,3345,3387,3541,4321,6135,8190,8654,8712,9517,12167,12211,14466,15637,16060,16231,16233

EU177827:363+C,3345,3387,3541,4321,8190,8712,11674,12161,12167,13697,13745,15637,16060,16076,16142,16233,16252

EU177828:106,169,222d,364C,1554,1602d,3345,3387,3541,4321,4339,4387,5143,8190,8712,12167,14293,15637,16060,16121,16129,16249

EU177829:169,363+C,1292,1602d,3345,3387,3541,4321,6501,6948,7801,8190,8712,12167,13526,15637,16135,16252

EU177830:169,222d,313A,364C,1602d,3345,3387,3541,4321,8190,8712,8882,10872,12167,13526,15637,16044

EU177831:169,222d,364C,1292T,1602d,3345,3387,3541,4321,6019,8190,8712,8900,12167,13472,14180,15637

EU177832:169,222d,364C,1602d,3345,3387,3541,4321,8190,8712,12167,15637,16135

EU177834:169,222d,364C,3343,3345,3387,3541,4321,7624,8190,8712,12025,12167,12175,14038,15637,15966,16167Y

EU177835:169,364C,3345,3387,3541,4321,8190,8712,12167,13880,14155,15637

EU177836:8,169,222d,364C,514,1602d,3345,3387,3541,4321,4822,8190,8712,12167,15094,15637,16068,16087,16111

EU177837:169,222d,364C,1602d,3088,3345,3387,3541,3798,4321,8190,8712,9098,10891,12167,12910,15637,15912,16057

EU177838:169,222d,352+G,364C,1602d,1860+A,3345,3387,3541,4321,6690,7194,8190,8712,12167,14368,15637,16303

EU177839:169,364C,1602d,3345,3387,3541,4321,8190,8242,8712,11187,11802,12167,15637,16110,16304

EU177841:8,169,364C,3345,3387,3541,4321,8190,8712,9562,12167,15398,15637,16115,16257

EU177842:169,222d,364C,3345,3387,3541,4321,7544,8190,8712,12167,15421,15637,16024,16052,16115,16257

EU177843:222d,364C,2057+C,3345,3387,3541,4321,8190,8712,9920,12167,13685,15637,16052,16115,16257

EU177844:169,222d,364C,2057+C,3345,3387,3541,4321,6048,8190,8712,12167,15084,15637,16052,16115,16257

EU177846:106,169,364C,2057+C,3345,3387,3541,4321,8190,8712,8961,12167,15637,15715d,16052,16078,16115,16117,16257

EU177847:169,364C,1602+AA,3345,3387,3541,4321,4977,6934,8190,8712,8893,12167,12254,15637,15966,16052,16115,16124,16198,16257

EU177848:169,364C,3345,3387,3541,3877,4321,8190,8525,8712,8864,10327,11454,12167,12653,15637,16052,16115,16198,16257

EU177849:169,222d,364C,3345,3387,3541,4321,4955,5634C,6204,6744,8190,8712,10710,12167,15637,16059C,16133,16187,16257

EU177850:169,364C,3345,3387,3541,4321,6204,6744,7852,8190,8712,10710,12167,13203,15637,16059C,16060,16127,16187,16257

EU177851:169,222d,364C,1461,2560,3345,3387,3541,4321,6204,6744,8190,8712,10710,11103,12167,12653,15637,15798,15987,16059C,16076,16187,16257

EU177852:169,364C,3345,3387,3541,4321,6204,6744,8190,8712,10710,12167,15637,16059C,16069,16076,16123,16187,16257

EU177853:169,170,222d,364C,3345,3387,3541,3942,4321,5158T,5763,6204,6744,8190,8712,10352,10710,12167,13753,15637,16052,16059C,16143,16187,16257

EU177854:169,222d,364C,1602d,1653,3345,3387,3541,4321,6204,6744,8190,8712,9502,10710,12167,14404,15637,16059C,16064,16187,16249,16257,16303

EU177855:169,222d,364C,3345,3387,3541,4321,5148,6204,6744,8190,8712,10710,12167,15637,16059C,16130,16187,16257

EU177856:169,173,222d,364C,364+C,3345,3387,3541,4321,5148,6744,8190,8247,8712,10261,10710,12167,15637,16051,16059C ,16115,16187,16257

EU177857:169,222d,364C,1325,1602d,1696,2601,3345,3387,3541,3720,4321,4783,5148,6204,6744,7400,8190,8712,10603,10710,12167,15159,15637,16059C,16060,16187,16250,16257,16303

EU177858:119,120,166,169,364C,3345,3351,3387,3541,3852,4321,6204,6405,6744,8190,8477,8712,9310,10662,10710,11832,12167,12294,12506,12767,13482,15637,15885d,16059C,16082,16187,16233,16257

EU177859:169,222d,364C,1555,3345,3387,3541,4321,4977,6204,6207,6744,8190,8712,8862,10710,12167,15637,16059C,16141,16187,16257

EU177860:169R,222d,364C,2087,3192,3345,3387,3541,4321,6204,6744,8190,8712,9482,9989,10710,11736,12167,13398,14581,15196,15637,16059C,16187,16257

EU177861:222d,364C,2087,2749,3192,3345,3387,3541,4321,6204,6316,6744,8190,8712,8986G,9989,10710,11736,12167,14581,15637,16059C,16187,16257

EU177863:163,364C,3345,3387,3541,4254,4321,8026,8190,8712,9091,9234,10881,12167,12782,12983,13007,15637,15884,16199,16257

EU177864:163,169,364C,3345,3387,3541,4254,4321,7660,8190,8712,9896A,12167,12983,13007,15637,16257

EU177865:163,169,204,364C,1292T,3345,3387,3541,4254,4321,8047,8190,8712,9896A,12167,12983,13007,15637,16139,16257

EU177866:222d,364C,1459,2560,3240,3345,3387,3417T,3541,4321,5503,7920,8190,8320,8372C,8407,8712,10719A,10929,11002,11091,11478,12167,12379,12435,12470,12677,12732,12752,12879,12926,13007,13821,14038,14110,15136,15629,15637,15955G,16060,16081,16257

EU177867:222d,364C,1459,2560,3240,3345,3387,3417T,3541,4321,5503,7920,8190,8320,8372C,8407,8712,10719A,10929,11002,11091,11478,12167,12379,12435,12470,12677,12732,12752,12879,12926,13007,13821,14038,14110,15136,15629,15637,15955G,16060,16081,16257

EU177868:8,106,166,173,206,233,234,249,250,297,301,364C,519,724,739,763,818,1160,1476,1494,1602d,1679,1826,1862A,1871,2018,2101,2323A,2560,2577,2636,2955,2979,2981,2990,2991,2992,3053,3073,3138,3147,3243,3327,3337,3345,3381,3387,3441,3537,3541,3552,3602,3795,3831,3876,3933,3978,3987,4002,4321,4330,4444,4564,4732,4735,4771,4873,4939,4990A,5287,5503,5533,5616,5745,5785,5892,5919,6000,6117T,6237,6342,6369,6381,6438,6462,6729,6774,6883,6924,7306,7332,7358,7360,7363,7516,7832,7853,8047,8170,8196,8212,8287,8310,8372C,8468,8496,8505,8516,8573,8712,8751,8986,9007,9040,9070,9247,9304,9482,9583,9604,9769,9893,9932C,9980,10041,10068,10073,10139,10155,10270,10324,10333,10447,10592,10623,10693G,10851,11002,11037,11070,11136,11202,11268,11331,11409,11421,11805,11844,12137,12167,12180,12236,12379,12435,12470,12471,12515,12624,12674,12677,12686,12752,12803,12902,12925T,12926,13007,13058A,13100,13103A,13106,13277,13373,13382,13435,13439,13556,13566,13586,13630,13679,13691,13694,13884,13910A,13911,14038,14068,14122,14131,14140,14257,14317,14413,14418,14505,14608,14827,14860,14899,15107,15136,15148,15289,15310,15328,15565,15581,15595,15607,15619,15629,15631,15637,15743,15753,15820,15953,15955G,15961,15996,16024,16051,16059,16060,16076,16084,16086,16104,16111,16115,16118,16119,16121,16123,16124,16132,16139,16140,16145d,16149,16198,16202+A,16231,16249,16250,16302,16303

EU177869:8,39,106,166,173,206,233,234,249,250,297,301,364C,364+C,519,739,763,818,1160,1476,1494,1602d,1679,1826,
1862A,1871,2018,2101,2560,2955,2979,2981,2990,2991,2992,3053,3073,3138,3147,3243,3318,3327,3337,3345,3381,3387,
3441,3537,3541,3552,3602,3795,3831,3876,3933,3978,3987,4002,4321,4330,4444,4564,4732,4735,4771,4873,4939,5275T,
5287,5503,5533,5616,5745,5785,5892,5919,6000,6117T,6237,6342,6369,6381,6438,6462,6498,6729,6774,6883,6924,7306,
7332,7358,7360,7363,7516,7832,7853,8047,8170,8212,8287,8310,8372C,8468,8496,8505,8573,8712,8751,8986,9007,9040,
9070,9247,9583,9604,9769,9893,9932C,9980,10041,10068,10139,10155,10270,10324,10333,10447,10592,10623,10693G,10851,
10890,10935,11002,11037,11070,11136,11202,11268,11331,11409,11421,11805,11844,12137,12167,12180,12236,12379,
12435,12470,12471,12515,12624,12674,12677,12686,12752,12803,12902,12925T,12926,13007,13058A,13100,13106,13277,
13382,13435,13439,13556,13566,13586,13679,13691,13694,13884,13910A,13911,14038,14068,14122,14131,14140,14234,
14257,14317,14371,14413,14418,14505,14608,14827,14833,14860,14899,15107,15136,15148,15289,15310,15328,15565,
15581,15595,15607,15619,15629,15631,15637,15743,15753,15820,15953,15955G,15961,15996,16024,16051,16052A,16059,
16060,16076,16084,16086,16087,16104,16111,16115,16118,16119,16121,16123,16124,16132,16139,16140,16143,16145d,
16149,16198,16202+A,16231,16234,16249,16250,16302,16303

EU177870:8,39,106,166,206,233,234,249,250,297,301,364C,519,739,763,818,1160,1476,1494,1602d,1679,1826,1862A,1871,
2018,2101,2560,2955,2979,2981,2990,2991,2992,3053,3073,3138,3147,3243,3318,3327,3337,3345,3381,3387,3441,3537,3541,
3552,3602,3795,3831,3876,3933,3978,3987,4002,4312,4321,4330,4444,4564,4732,4735,4771,4873,4939,5287,5503,5533,5616,
5745,5785,5892,5919,6000,6117T,6237,6342,6369,6381,6438,6462,6729,6774,6883,6924,7306,7332,7358,7360,7363,7501,
7516,7832,7853,8047,8170,8212,8287,8310,8372C,8468,8496,8505,8573,8712,8751,8986,9007,9040,9070,9247,9583,9604,
9769,9893,9932C,9980,10041,10068,10139,10155,10270,10324,10333,10447,10592,10623,10690,10693G,10851,10890,10935,
11002,11037,11070,11136,11202,11268,11331,11409,11421,11805,11844,12137,12167,12180,12236,12341,12379,12435,
12470,12471,12515,12624,12674,12677,12686,12752,12803,12902,12925T,12926,13007,13058A,13100,13106,13277,13382,
13435,13439, 13556,13566,13586,13679,13691,13694,13884,13910A,13911,14038,14068,14122,14131,14140,14257,
14317,14371,14413,14418,14505,14608,14827,14860,14899,15107,15136,15148,15289,15310,15328,15565,15581,15595,
15607,15619,15629,15631,15637,15743,15753,15820,15953,15955G,15961,15996,16024,16051,16059,16060,16076,16084,
16087,16104,16111, 16115,16118,16119,16121,16123,16124,16132,16139,16140,16143,16145d,16149,16198,16202+A,16231,
16234,16249,16250, 16302,16303

FJ971083:169,222d,244T,364C,1459,2560,3240,3345,3387,3417T,3541,4321,5503,7920,8190,8320,8372C,8619,8712,10719A,
10929,11002,11091,11768,12167,12435,12470,12677,12752,13007,13508,13622,14038,14110,15136,15637,15955G,16114,
16257

FJ971084:8,106,166,173,201,215+TC,235+T,250,297,301,364C,782,895,1601-1602d, 2044,2560,2570,2981,3304, 3345,3387,
3441,3541,3552,3602,4192,4321,4363,4855,5148,5503,5616,5745,5892,5937,6081,6120,6162,6438,6477,6774,6852,6985,7332,
7358,7361,7516,7933,8372C,8496,8712,8769,9007,9178,9482,9661,9866,9980,10333,10693G,10929,11002,11136,11394,11421,
11757,11844,12047,12167,12236,12470,12471,12624,12677,12686,12752,12803,12902,13007,13058A,13130,13156,13262,
13460,13508,13719,14002,14038,14053,14459,14626,14827,14908,14953,15067,15136,15581,15619,15626,15629,15637,
15820,15848,15902,15953,15955G,16059,16078,16086,16087,16123,16124,16129,16137,16139,16202+A,16233,16250,16252,
16266,16303

FJ971085:8,106,166,173,201,215+TC,235+T,250,297,301,364C,782,895,1601-1602d,2044,2560,2570,2981,3304,3345,3387,3441,
3541,3552,3602,4192,4321,4363,4855,5503,5616,5745,5892,5937,6081,6120,6162,6438,6477,6774,6985,7332,7358,7361,7516,
7933,8372C,8496,8712,8769,9007,9178,9482,9661,9866,9980,10333,10693G,10929,11002,11136,11394,11421,11757,11844,
12047,12167,12236,12470,12471,12624,12677,12686,12752,12803,12902,13007,13058A,13130,13156,13262,13460,13508,
13719,14002,14038,14053,14459,14626,14827,14908,14953,15067,15136,15581,15619,15626,15629,15637,15820,15848,
15902,15953Y,15955S,16059,16078,16086,16087,16123,16124,16129,16137,16139,16202+A,16233,16250,16252,16266,16303
FJ971088:8,106,166,173,206,233,234,249,250,297,301,364C,519,724,739,763,818,1160,1476,1494,1602d,1679,1826,1862A,
1871,2018,2101,2560,2577,2636,2955,2979,2981,2990,2991,2992,3053,3073,3138,3147,3243,3312,3327,3337,3345,3381,3387,
3441,3537,3541,3552,3602,3795,3831,3876,3933,3978,3987,4002,4321,4330,4444,4564,4732,4735,4771,4873,4939,4990A,
5287 ,5503,5533,5616,5745,5785,5892,5919,6000,6117T,6237,6342,6369,6381,6438,6462,6729,6774,6883,6924,7306,7332,
7358, 7360,7363,7516,7832,7853,8047,8170,8196,8212,8287,8310,8372C,8468,8496,8505,8516,8573,8712,8751,8986,9007,
9040, 9070,9247,9304,9482,9583,9604,9769,9893,9932C,9980,10041,10068,10073,10139,10155,10270,10324,10333,10447,
10592, 10623,10693G,10851,11002,11037,11070,11136,11202,11268,11331,11409,11421,11805,11844,12137,12139,12167,
12180, 12236,12379,12435,12470,12471,12515,12624,12674,12677,12686,12752,12803,12902,12925T,12926,13007,13058A,
13100, 13103A,13106,13277,13373,13382,13435,13439,13556,13566,13586,13630,13679,13691,13694,13884,13910A,13911,
14038, 14068,14122,14131,14140,14257,14317,14413,14418,14505,14608,14827,14860,14899,15107,15136,15148,15159,
15289, 15310,15328,15565,15581,15595,15607,15619,15629,15631,15637,15743,15753,15820,15953,15955G,15961,15996,
16024, 16051,16059,16060,16076,16084,16086,16104,16111,16115,16118,16119,16121,16123,16124,16132,16139,16140,1
6145d, 16149,16198,16202+A,16231,16249,16250,16302,16303
GQ129207:166,363+C,3345,3387,3541,4321,4744,8190,8712,12167,15637,16087,16133
GQ129208:353G,364C,3345,3387,3541,4321,8190,8712,9136,12160,12167,15637

GU947007:169,190,222d,364C,1602d,1865,2632T,3345,3387,3541,3810,4321,5044,7241,8190,8712,9064,9238,12167,14218,
14233,15637,15987d,16059+A,16070,16076A,16078

GU947008:169,364C,1602d,3345,3387,3541,4321,4333,5044,8190,8712,9731,12167,15637,15987d,16059+A,16076A,16078

GU947010:169,352+G,364C,1602d,3345,3387,3541,4321,8190,8712,10462,12167,15637,15987d,16059+A,16076A,16078,16149

GU947012:169,364C,1602d,3345,3387,3541,4321,6327,8190,8712,9731,12167,12629,13280,15637,15987d,16059+A,16076A,
16078,16233

GU947014:169,364C,1602d,3345,3387,3541,4321,4333,5226,8190,8712,9731,12167,13784T,15637,15987d,16059+A,16076A,
16078

GU947018:169,364C,1602d,3345,3387,3541,4321,8190,8712,10462,12167,14218,14233,15637,15987d,16059+A,16076A,16078,16149

GU947019:166,169,222d,537,567,3345,3387,3541,4321,8190,8712,8927,12167,13901,15637

GU947020:169,364C,1602d,2057+C,3345,3387,3541,4159,4321,7029,8190,8407,8712,11430,12167,14429,15637,16052,16115,16141,16149,16257

GU947021:169,364C,1602d,2055,3345,3387,3541,4321,4756C,4757C,5604,5718,7011,7167R,8190,8712,12025,12167,12178,13091A,13520,15637,16138,16225,16228C

GU985279:190,224d,250,301,302,365d,1130,1483,2147,2560,2587,3345,3381,3387,3541,3552,4254,4295,4321,4678,5158,5503,5745,5892,5901,6162,6438,7358,7954,7996,8190,8238,8360,8372C,8712,10128,10693G,11002,11142,11742,12018,12167,12379,12435,12470,12471,12527,12677,12752,13007,13823,14038,14131T,14582,14875,15136,15629,15637,15675,15953,15955G,15996,16021,16051,16053,16060,16076,16087,16123R,16124,16143,16233,16257,16266,16303

HM045018:3189,3345,3387,3541,4321,8190,8712,12167,15637

HQ025805:169,222d,364C,589d,3345,3387,3541,3561,3562G,4108,4321,4327,5855,6204,6744,7960,8190,8712,10710,12167,12272,14418,15637,16059C,16187,16257

HQ184030:169,222d,364C,2560,3240,3345,3387,3417T,3541,4321,5503,6438,7920,8190,8320,8372C,8712,8881,10719A,10929,11002,11091,12167,12435,12470,12677,12752,13007,14038,14110,15136,15158,15629,15637,15923,15955G,16257

HQ184031:169,222d,364C,2560,3240,3345,3387,3417T,3541,4321,5503,6438,7920,8190,8320,8372C,8712,8881,10719A,10929,11002,11091,12167,12435,12470,12677,12752,13007,14038,14110,15136,15158,15629,15637,15923,15955G,16257

HQ184032:169,222d,364C,2560,3240,3345,3387,3417T,3541,4321,5503,6438,7920,8190,8320,8372C,8712,8881,10719A,10929,11002,11091,12167,12435,12470,12677,12752,13007,14038,14110,15136,15158,15629,15637,15923,15955G,16257

HQ184034:169,222d,244T,364C,1459,2560,3240,3345,3387,3417T,3541,4321,5503,7920,8190,8320,8372C,8619,8712,10719A,10929,11002,11091,11768,12167,12435,12470,12677,12752,13007,13508,13622,14038,14110,15136,15637,15955G,16114,16257

HQ184035:169,222d,244T,364C,1459,2560,3240,3345,3387,3417T,3541,4321,5503,7920,8190,8320,8372C,8619,8712,10719A,10929,11002,11091,11768,12167,12435,12470,12677,12752,13007,13508,13622,14038,14110,15136,15637,15955G,16114,16257

HQ184036:222d,364C,1459,2560,3240,3345,3387,3417T,3541,4321,5503,7920,8190,8320,8372C,8407,8712,10719A,10929,11002,11091,11478,12167,12379,12435,12470,12677,12732,12752,12879,12926,13007,13821,14038,14110,15136,15629,15637,15955G,16060,16081,16257

HQ184037:222d,364C,1459,2560,3240,3345,3387,3417T,3541,4321,5503,7920,8190,8320,8372C,8407,8712,10719A,10929,11002,11091,11478,12167,12379,12435,12470,12677,12732,12752,12879,12926,13007,13821,14038,14110,15136,15629,15637, 15955G,16060,16081,16257

HQ184038:222d,364C,1459,2560,3240,3345,3387,3417T,3541,4321,5503,7920,8190,8320,8372C,8407,8712,10719A,10929,11002,11091,11478,12167,12379,12435,12470,12677,12732,12752,12879,12926,13007,13821,14038,14110,15136,15629,15637,15955G,16060,16081,16257

HQ184039:169,222d,364C,471,1459,2560,3240,3345,3387,3417T,3541,4321,5503,5718,6660,7832,7920,8190,8320,8372C,8712,10719A,10929,11002,11091,12167,12435,12470,12482,12677,12752,13007,14038,14110,15092,15100A,15136,15629,15637,15955G,16198,16257

JN817298:106,169,222d,364C,1593,3191,3345,3387,3541,4321,6237,8190,8712,12167,15637,16052,16115,16257

JN817299:169,222d,250,364C,3345,3387,3541,4321,4372,6237,6453,7400,8190,8712,11070,12167,15637,16052,16115,16257

JN817300:169,222d,364C,3345,3387,3541,3877,4321,8190,8712,11475,12167,15637,15966,16052,16081,16115,16124,16129,16198,16202,16257

JN817302:169,364C,3345,3387,3541,4321,7544,8190,8712,9102,12167,14525T,14533,14992,15637,15846,16024,16115

JN817303:169,222d,364C,2057+C,3345,3387,3541,3684,4321,4453,8190,8712,11028,12167,15064,15637,16052,16115,16248,16249,16257

JN817304:169,364C,1602d,3345,3387,3541,4321,4858,6237,6333,7762,8190,8712,12167,15149,15637,16052,16115,16257

JN817305:169,364C,2967,3345,3387,3541,4321,7544,8190,8712,9433,12167,12362,13203,14525T,15637,16024,16052,16058,16115,16149,16257,16304

JN817306:8,169,364C,3345,3387,3541,4321,8190,8712,12167,15637,15941,16115,16250,16257

JN817307:24,169,222d,364C,3345,3387,3406,3541,4321,5032,8190,8712,9677,12167,14386,14600G,15328,15637,15811,16052,16115,16124,16198,16257

JN817308:8,169,222d,364C,1602+A,2057+C,2080,3345,3387,3541,4321,8190,8712,10276,12167,15637,16052,16060,16110,16115,16124,16257

JN817309:169,222d,364C,1326,3345,3387,3541,4321,8190,8712,11544,12167,13884,15637,16052,16055,16115,16124,16141,16198,16257

JN817310:169,222d,364C,1326,3345,3387,3541,4321,8190,8712,11544,12167,15637,16052,16055,16115,16124,16141,16198,16257,16318

JN817311:169,222d,364C,1326,3345,3387,3541,4321,8190,8712,10895A,11544,12167,15464,15637,16052,16055,16115,16124,16141,16198,16257

JN817312:169,222d,364C,665,2057+C,3345,3387,3541,4321,8190,8712,10519,12167,13061,13898,15637,16052,16115,16257,16262

JN817313:169,222d,267T,364C,2057+C,3345,3387,3541,4321,8190,8712,12167,13880,15637,16052,16115,16257

JN817314:169,222d,364C,756,2057+C,3345,3387,3541,4321,6388,8190,8712,9840,12167,14327,15637,16052,16115,16257

JN817315:8,169,222d,364C,2057+C,3345,3387,3541,4321,8190,8712,11190,12167,15637,16052,16115,16257

JN817316:169,222d,364C,2057+C,2584,3345,3387,3541,4321,8190,8712,10603,11024,12167,14582,15637,15976,16052,16115,16257

JN817317:8,169,364C,1300,2057+C,3345,3387,3541,4321,4742,7322,8190,8712,9604,12167,15637,16052,16115,16121,16169,16257

JN817318:169,222d,364C,2057+C,3345,3387,3541,3546A,3942,4321,7068,8190,8712,12167,12476,15637,16052,16115,16257

JN817319:169,364C,3345,3387,3541,4321,8190,8712,10246A,12167,15637,16115,16124,16257

JN817320:222d,364C,3210,3345,3387,3541,4321,7544,8190,8712,12167,15149,15328,15637,16024,16052,16115,16257

JN817321:8,169,364C,1602d,3345,3387,3541,4321,4858,6237,8190,8712,9514,10516,12167,15637,16052,16115,16250,16257

JN817322:106,169,364C,1326,1876,3345,3387,3541,4321,7657,8190,8712,9070,11544,12167,15637,16018,16052,16055,16115,16124,16141,16198,16257

JN817323:169,222d,353G,364C,1602d,1881,3345,3387,3541,4321,4349,4882,6019,8190,8328,8712,9722,12167,12407,12730,15421,15637,15958,16052,16115,16124,16198,16257

JN817324:169,364C,3345,3387,3541,4321,7544,8190,8350,8712,12167,12229,15637,16024,16115,16124,16257

JN817325:169,364C,3345,3387,3541,4321,8190,8712,10498,10926,11791,12167,15637,16052,16115,16124,16137,16208,16257

JN817326:169,364C,2192,3345,3387,3541,4321,8190,8334,8712,11963,12167,15100,15158,15637,16052,16114,16115,16124,16257

JN817327:106,169,222d,364C,3345,3387,3541,4321,7544,7818,8190,8712,12167,14053,15637,15948,16115

JN817328:169,364C,1602d,3345,3387,3541,4321,5188,8190,8712,8796A,12167,12362,13733,15637,16052,16057,16115,16124,16257

JN817329:169,222d,364C,3345,3387,3541,4321,7757,8190,8712,9385,12167,12494,14045,15092,15406,15637,16052,16257

JN817330:169,364C,1602d,3345,3387,3541,4321,4858,6237,8190,8712,11457,12167,15637,16052,16115,16257

JN817331:169,222d,364C,2057+C,3345,3387,3541,4167,4321,8190,8235,8251,8712,9840,11791,12167,15637,16052,16115,16135,16233,16257

JN817332:8,169,222d,364C,2057+C,3345,3387,3541,4321,8190,8712,11190,12167,15637,16052,16115,16257

JN817333:169,222d,364C,2057+C,3345,3387,3406,3541,4321,8190,8712,10563,12167,12732,13164,15637,16052,16115,16257

JN817334:169,222d,364C,3345,3387,3541,4321,4947,7544,8190,8712,10579,11532,12167,13373A,15637,15848,16024,16052,16115,16257,16303

JN817335:169,173,222d,364C,2057+C,3345,3387,3541,4114,4321,8190,8712,11190,12167,15637,16052,16115,16257

JN817336:8,169,222d,364C,2057+C,3345,3387,3541,4321,5017,5227,5814,8190,8712,12167,13125,13673,14694,14905,15527,15637,16052,16115,16257

JN817337:169,222d,364C,2057+C,3345,3387,3406,3541,4321,7299,8190,8712,10563,12167,12732,15637,16052,16115,16141,16257,16304

JN817338:169,222d,353G,364C,2025,2057+C,3345,3387,3541,4321,7068,7511,8190,8712,9808,12167,12452,12476,15637,16052 ,16115,16143,16166,16257

JN817339:169,222d,364C,711,2057+C,3345,3387,3541,4321,8190,8712,9445,10857A,12167,15637,16052,16115,16257,16266

JN817340:169,222d,364C,2057+C,3345,3387,3541,4321,4742,7322,8160,8190,8712,9604,11956,12167,15637,16052,16115,16121,16169,16257

JN817341:169,364C,2057+C,3345,3387,3541,4321,8190,8712,12167,15637,16018,16052,16115,16257

JN817342:169,222d,364C,2057+C,3345,3387,3406,3541,4321,7299,8190,8712,10563,12167,12732,15637,15656A,16052,16115,16257

JN817343:169,364C,3345,3363,3387,3541,4321,8190,8712,9638,11850,12167,12494,14212,14611,15412,15637,16052,16257

JN817345:106,169,222d,364C,1842,2057+C,3345,3387,3541,4321,8190,8198Y,8669,8712,8861,12167,15637,16052,16115,16257

JN817346:169,222d,364C,3345,3387,3541,4321,8190,8712,10530,10797,12167,14459,14821,15637,16052,16055,16115,16124,16135,16198,16257

JN817347:169,222d,364C,2057+C,3345,3387,3541,4321,8190,8712,9686,12167,12902,13425,15216,15637,16052,16115,16257,16304

JN817348:169,222d,364C,3345,3387,3541,4141,4321,5898,6705,7544,8190,8712,9136,9658,9731,10605,12167,13295,13984,15637,16052,16115,16249,16257

JN817349:8R,169,364C,3345,3387,3541,4321,7544,8190,8712,12167,13203,14525T,15637,16024,16052,16058,16115,16140,16149,16257

JN817351:169,178,222d,364C,3345,3387,3541,4321,5638,7544,8190,8712,12167,14758,15637,16024,16052,16115,16257,16293

JQ437479:169,190,224d,250,301,302,365d,1130,1483,2560,2587,3190,3304,3345,3381,3387,3541,3552,4321,4678,5158,5503,5745,5892,5901,6162,6438,6453,7358,7954,7996,8190,8238,8360,8372C,8712,10128,10693G,11002,11142,12018,12167,12379,12435,12470,12677,12752,13007,13541,13823,14038,14131T,14875,15136,15473,15629,15637,15675,15953,15955G,15996,16051,16060,16076,16087,16124,16233,16257,16266

KC153972:169,177,364C,1602d,3345,3387,3541,4321,8190,8712,12167,15637,16051,16052

KC153974:169,364C,1602d,3345,3387,3541,4321,5226,8190,8712,8832,10884,11514,12167,13487,15637,16140

KC153976:169,236,364C,1602d,3282,3345,3387,3541,4321,6336,8190,8712,9604,12167,15637

KC153977:222d,353G,364C,3345,3387,3541,4321,8190,8712,10686,12167,14418,15637,16064,16249

KF163061:169,215+TC,232+C,364C,1601-1602d,3345,3387,3541,4321,6052,7544,8190,8712,12167,12470,12471, 14350,15637,16024,16052,16086,16087,16115,16202+A,16257

KF163063:169,215+TC,235+T,364C,1601-1602d,3345,3387,3541,4321,7544,8190,8712,12167,15637,16024, 16052,16115,16202+A,16257,16303

KF163064:169,190,215+TC,235+T,364C,498,1601-1602d,3071,3345,3387,3541,4321,7544,8190,12167,12527,13203,13302,14525T,15637,16024,16052,16058,16059,16086,16115,16204+G,16318

KF163065:169,190,215+TC,235+T,364C,498,1601-1602d,3071,3345,3387,3541,4321,4363,7544,8190,12167,12470,12471,12527,13203,13302,14053,14525T,15637,16024,16052,16058,16086,16087,16115,16202+A,16318

KF163066:169,190,215+TC,235+T,364C,498,1601-1602d,3071,3345,3387,3541,4321,7544,12167,12470,12471,12527,13203,13302,14525T,15637,16024,16052,16058,16087,16115,16202+A,16318

KF163067:169,173,190,215+TC,235+T,364C,498,1601-1602d,3345,3387,3541,4321,7544,8190,12167,12470,12471,12527, 13203,13302,14525T,15637,16024,16052,16058,16086,16087,16115,16202+A,16257,16318

KF163068:169,190,215+TC,235+T,364C,498,1601-1602d,3272,3345,3387,3415-3454N,3541,4321,4842Y,7544,8190,12167,12363,12470,12471,12527,13203,13302,14525T,15637,16024,16052,16058,16086,16087,16115,16202+A,16257,16318

KF163070:169,190,215+TC,235+T,364C,498,1601-1602d,3345,3387,3541,4321,7544,7624,8190,12167,12470,12471,12527,13203,13262,13302,14525T,15637,16024,16052,16058,16086,16087,16115,16202+A,16257,16318

KF163071:169,190,216Y,222+CC,235+T,364C,498,1601-1602d,3272,3345,3387,3541,4321,7544,8190,12167,12363,12470,12527,13203,13302,14525T,15637,16024,16052,16058,16115,16204+G,16257,16318

KF163072:169,215+TC,235+T,364C,1601-1602d,3345,3387,3541,4321,4588,6027,8190,8712,9908,10909,12167, 15462,15637,16052,16086,16087,16115,16123,16124,16202+A,16257

KF163073:169,215+TC,235+T,364C,1601-1602d,3345,3387,3541,4321,7544,8190,8712,12167,12470,12471,13203, 14525T,15158,15637,16024,16052,16115,16202+A,16257

KF163075:169,215+TC,235+T,364C,1601-1602d,3345,3387,3541,4321,5227,7544,8190,8712,12167,12404,12471, 13203, 14525T,14773,15637,16024,16052,16058,16059,16115,16202+A,16257

KF163077:169,190,215+TC,235+T,364C,498,1077C,1601-1602d,3345,3387,3541,4321,6393,7544,8190,9097,12167,12527,13203,13302,14525T,15637,16024,16052,16058,16086,16087, 16115,16123,16202+A,16257,16318

KF163078:169,190,215+TC,235+T,364C,498,1601-1602d,3272,3345,3387,3541,4321,7544,8190,12167,12363,12470,12471,12527,13203,13302,14525T,15637,16024,16052,16058,16087,16115,16123,16124,16202+A,16257,16318

KF163079:169,173,190,215+TC,235+T,364C,498,1601-1602d,3345,3387,3541,4321,7544,8190,12167,12527,13203,13302,14525T, 15637,16024,16052,16058,16115,16202+A,16257,16318

KF163080:169,190,215+TC,235+T,364C,498,1601-1602d,3345,3387,3541,4321,6477,7544,8190,12167,12470,12471,12527,13203,13302,14525T,15637,16024,16052,16058,16086,16087,16115,16124,16202+A,16257,16318

KF163081:169,215+TC,235+T,364C,1601-1602d,3345,3387,3541,4321,6237,8190,8712,10349, 10659,12167,12470, 15637,16052,16086,16087,16115,16123,16202+A,16257

KF163082:169,215+TC,235+T,300,364C,1601-1602d,3345,3387,3541,3684,4321,8190,8712,9203,12167,12470,12471,15637,16052,16115,16202+A,16250,16257,16262

KF163083:169,190,215+TC,235+T,364C,498,1601-1602d,3272,3345,3387,3541,4321,7516,8190,12167,12363,12527,13203,13302,14525T,15637,16024,16052,16058,16086,16087,16115,16202+A,16257,16318

KF163084:169,215+TC,235+T,364C,1601-1602d,3345,3387,3541,4321,7544,8190,8712,12167,13397,15637, 16024,16052, 16059,16070,16086,16087,16115,16202+A,16257

KF163085:169,190,215+TC,235+T,250,297-298N,364C,498,1601-1602d,3345,3387,3541,4321,7544,8190,12167,12470,12471,12527,13007,13203,13302,14525T,15637,15953,15955G,16024,16052,16058,16087,16115,16202+A,16257,16318

KF163086:169,190,215+TC,235+T,364C,498,1601-1602d,3345,3387,3541,4321,7544,8190,12167,12527,13203,13302,14525T,15637,16024,16052,16058,16115,16124,16202+A,16257,16318

KF163090:169,190,215+TC,235+T,364C,498,1601-1602d,3272,3345,3387,3483,3541,4321,7544,8190,12167,12470,12471,12527,13203,13302,14525T,15637,16024,16052,16058,16086,16087,16115,16202+A,16257,16318

KF163092:169,215+TC,235+T,364C,1601-1602d,3345,3387,3541,4321,7544,8190,8712,9503,12167,13262,15637,16024,16052, 16086,16087,16115,16202+A,16257

KF163093:169,215+TC,235+T,300,364C,1601-1602d,3345,3387,3541,3684,4321,8190,8712,9203,12167,12470, 12471,15637, 16052,16115,16202+A,16249,16257,16262

KF163094:169,179,215+TC,235+T,364C,1483,1601-1602d,3155Y,3345,3387,3541,4321,7544,8190,8712,10462,12167, 12470, 12471,13203,14525T,15637,16024,16052,16058,16115,16202+A,16257

KF926377:169,364C,1602d,3345,3387,3541,4060,4321,8063A,8065A,8066A,8069,8190,8712,12167,13734,15637,16053,16063, 16131
