# Supplementary material for: Recovery of mitogenomes from whole genome sequences to infer maternal diversity in 1883 modern taurine and indicine cattle
Source: Sci Rep. 2022 Apr 4;12:5582. doi: 10.1038/s41598-022-09427-y (PMC8980051; doi:10.1038/s41598-022-09427-y)
Supplement: Supplementary file 5 — Supplementary Tables. [file 41598_2022_9427_MOESM5_ESM.pdf]

Table S3. Haplogroups and source of samples of 1883 animals in the filtered dataset determined from the variant sequence using MitoToolPy

Breed	Source	Haplogroups											
		N	I1	I2	P2	Q1	Q2	T1	T2	T3	T4	T5	T6
Achai	PAK	4	1	1						2			
Afrikander	AUS	3						3					
Alentejana		1								1			
Angus	AUS,CAN,NZL,USA	103								97			6
Angus German	USA	1								1			
Angus Lowline	AUS	2						2					
Angus Red	CAN,NZL,USA	10								10			
Angus Simmental		1								1			
Ankole	UGA	9						9					
Australian Lowline	GBR	1											1
Ayrshire Finnish	FIN	1								1			
Beef master	USA	2						1		1			
Belgian Blue Holstein		3								3			
Belgian Blue Limousin	IRE	1								1			
Benishangul	ETH	3						3					
Bhagnari	PAK	4	3	1									
Blonded Aquitaine	FRA	15						2		13			
Bohai Black		5	2							3			
Bohuskulla	SWE	3								3			
Boran	KEN	19						19					
Boskarin		1								1			
Brahman	AUS	18	1					12		5			
Brown Swiss	FRA,CHE,DEU,ITA,USA	84						1		81		2	
Buryat	RUS	20	2						2	16			
Busa	FIN	8								8			
Cabannina	ITA	2								2			
Chaidamu Yellow		5	2							3			
Charolais	CAN,FRA,USA	33							1	32			
Charolais Angus		1								1			
Charolais Red Angus		1								1			
Chianina	ITA	5				2	2			1			
Cholistani	PAK	5	4	1									
Cloned-polled Dairy Bull		2								2			
Composite	CAN,AUS	13	4					6		3			
Corriente		2								2			
Costeno ConCuernos		2						1		1			
Crossbreed	CAN,FRA	21								21			
Crossbreed(HO62.5%;MO25%;JE12.5%)		1								1			
Dabieshan		3	2							1			
Dajal	PAK	4	4										
Danish Red Dairy		2								2			
Dengchuan	CHN	1								1			
Deutsches Schwarzbuntes Niederungsrind	DEU	47							1	46			
Dexter	AUS	2						1		1			

Breed	Source		Haplogroups											
			N	I1	I2	P2	Q1	Q2	T1	T2	T3	T4	T5	T6
Dhanni	PAK		5	3	2									
Dianzhong			5	2	1						2			
Drought master	AUS		1								1			
Eastern Finncattle		0	5								5			
Eringer	CHE		3								3			
Evolčne	CHE		2							1	1			
Fjäll	SWE		11								11			
Fleckvieh	DEU		15							1	14			
Fogera	ETH		1						1					
Gabrialli	PAK		4	1	1						2			
Galloway			1								1			
Galloway Belted	AUS		3	1							2			
Gelbvieh	CAN		13								13			
Gir			1		1									
Goffa	ETH		3						3					
Guangfeng			4	3							1			
Guanling	CHN		1								1			
Hanwoo			24			1			2	2	19			
Hariana			1	1										
Hasake	CHN		5								5			
Hereford	AUS,CAN,NZL,RUS,USA		44	2	1						41			
Hereford Miniature	AUS		2								2			
Holstein	AUS,CAN,CHE,CHN,DEU,DNK , FRA,GBR, NLD, USA		267	5			1		6	4	251			
Holstein Charolais	DEU,		19								19			
Holstein Friesian	DEU,USA		35							1	34			
Holstein Hereford	USA		9								9			
Holstein Jersey F1Crossbred	USA		1								1			
Holstein LimousinF1 Crossbred	USA		2								2			
Holstein Red	DEU		3						1		2			
Holstein Simmental			4								4			
Iran Admixed			9				4		1		4			
Japanese Native			9						1		8			
Jersey	AUS,USA		27						4		23			
Jian			4	3							1			
Jiaxian Red			5	3							2			
Jinjiang			4	3							1			
Kalmykian	FIN		10							1	8		1	
Kangayam			1		1									
Kazakh			9	2					1	3	3			
Kazakh Whiteheaded	RUS		5								5			
Kenana	SUD		5						5					
Lagune			1						1					
Leiqiong			3	3										
Limia			1								1			
Limonero			9						1		8			
Limousin	CHE,FRA,		27								26		1	
Limousin Hereford			2								2			
Limousin Holstein	CHE		1								1			
Limousin x Brown Swiss		0	1								1			

Breed	Source	Haplogroups											
		N	I1	I2	P2	Q1	Q2	T1	T2	T3	T4	T5	T6
Lingnan		7	6						1				
Lohani	PAK	1	1										
Luxi		5	5										
Maine Anjou	USA	1								1			
Maremmana	USA	1								1			
Maronesa	USA	1								1			
Menggu	CHN	11							1	10			
Modern Danish Red	DNK	23							1	22			
Mongolian		7	1						1	5			
Montbeliarde	FRA	17							1	15		1	
Muturu	NGR	10						10					
Nanyang		4	3						1				
Nari Master	PAK	4	2	1						1			
NDama	BEN,GUI,	12						12					
Nelore		2						1		1			
Nordic Red Cattle		2								2			
Normande	FRA	7								7			
Northern Finncattle	FIN	9								9			
Norwegian Red	NOR	222							6	216			
Nuer	ETH	1						1					
Ogaden	ETH	9						9					
Original Braunvieh	CHE	17							3	12		2	
Ottonese	ITA	1							1				
Pajuna		1								1			
Parthenaise	FRA	2								2			
Pezzata Rossa Italiana		1								1			
Piedmontese	CHE	2								2			
Piedmontese Normande		1								1			
Pinzgauer	CHE	1								1			
Podolian-Serbia	FIN	10								4		6	
Podolica		1								1			
Qinchuan	CHN	2								2			
RedSindhi	PAK	3	2	1									
Rendena	ITA	2								2			
Ringamålako	SWE	2								2			
Rödkulla	SWE	9							1	8			
Romagnola	IRL,ITA	10				1	1	2	1	5			
RougeDesPres	FRA	3								3			
Sahiwal	PAK	5	2	3									
Salers	FRA	4								4			
San Martinero		2						1		1			
Santa Gertrudis	AUS	1								1			
Sayaguesa		1								1			
Scottish Highland	CHE, GBR	7								7			
Senepol	AUS	12						5		7			
Shaiwal	IND	2		2									
Sheko	ETH	3						3					
Shorthorn		1	1										
Sichuan Indigenous		1	1										

Breed	Source	Haplogroups											
		N	I1	I2	P2	Q1	Q2	T1	T2	T3	T4	T5	T6
Sikias		1							1				
Simmental	AUT,CAN,CHE	32								32			
Simmental Fleckvieh Pezzatarossa	AUT,CHE,ITA	3								3			
Simmental x Angus	CHE	1								1			
Somba Tongalese Modern		1						1					
South Anatolian Red		1							1				
Swedish Red	SWE	1								1			
Swiss Fleckvieh	CHE	1								1			
Texas Longhorn		1								1			
Tharparkar	PAK,IND	7	7										
Tharparker Modern		1	1										
Tibetan Cattle		2								2			
Traditional DanishRed	DNK	1								1			
Tuxer	CHE	1								1			
Tyrolean Grey	AUT,CHE,ITA	7								7			
Uganda Admixed		26						26					
Ukrainian Grey	FIN	8								8			
Unknown		126	4	1				6	7	108			
Väneko	SWE	5								5			
Vechur		1		1									
Vorderwaelder	DEU	1								1			
Wagyu Modern		1									1		
Wandong		2	2										
Wannan		7	3							4			
Weining		5	3							2			
Wenshan		4	4										
Western Finncattle		4								4			
Xizang	CHN	2								2			
Xuanhan		5	2							3			
Yakut	FIN,RUS,	35							6	29			
Yanbian	CHN	8						1		7			
Yaroslavl	RUS	10								10			
Zaobei		5	4							1			
Zebu	ETH	2						2					
Zebu Indian		1	1										
	Total	*1883*	*112*	*19*	*1*	*8*	*3*	*167*	*50*	*1502*	*1*	*13*	*7*

AUS Australia, AUT Austria, BEN Benin, CAN Canada, CHN China, DNK Denmark, ETH Ethopia, FIN Finland. FRA France, GUI Guinea, DEU Germany, IND India, IRE Ireland, NLD Netherlands, NZL New Zealand, GBR United Kingdom, NGR Nigeria, PAK Pakistan, RUS Russia, SCL Scotland, SPN Spain, SWE Sweden, CHE Switzerland, UGA Uganda, USA United States of America

Table S4. Genotypes and allele depth at key position distinguishing the haplogroup T1b1 and T3r for Jersey with T1 haplogroup.

		POS				
	**Alleles**	**7544**	**16024**	**16052**	**16115**	**16257**
	**REF:**	**G**	**G**	**C**	**T**	**T**
**Animal**	**ALT:**	**A, C**	**A, T**	**T**	**C**	**C**
SAMN08612491		1/1:9,234	1/1:4,302	1/1:3,309	1/1:0,272	1/1:3,201
SAMN19491856		0/1:3,32,0	1/1:0,23	1/1:0,22	1/1:0,16	1/1:0,22
SAMN08612497		1/1:5,132	1/1:1,122	1/1:0,127	1/1:0,125	1/1:2,96
SAMN19491865		1/1:1,205	1/1:0,174	1/1:0,172	1/1:0,152	1/1:1,139

Table S5. Annotation of the private variants* common to a specific groups of animals within a haplogroup.

Haplogroup (N)	Bp position on ARS (BRS)	N	Annotation
I_1 (112)	1497(1495)	64	Spec, NCTE, Mt rRNA
	5707(5705)	9	Spec, Syno, COX1
	8648(8646)	64	Spec, Miss, ATP6
	14029 (14027)	64	NonS, Miss, ND6
	16086(16084)	115	NonS, Up/down
	16112 (16110)	4	NonS, Up/down
I_2 (19)	14833 (14831)	13	Spec, Miss, CYTB
	16087 (16085)	19	NonS, Up/down
Q_1 (6)	5718 (5716)	2	NonS, Miss, COX1
T_1 (57)	6388 (6386)	3	Syno, COX1
	3684 (3682)	13	Spec, Syno, ND1
	15675 (15673)	8	NonS, NCTE,tRNA
	16262 (16260)	9	NonS, Up/Down
	2579 (2577)	7	Spec, NCTE rRNA
	4714 (4712)	7	Spec, Miss, ND2
	6882 (6880)	7	Spec, Miss, COX1
	10435 (10433)	7	Spec, Miss, ND4L
T_1b (3)	4984 (4982)	2	NonS, Miss, tRNA
	5898 (5896)	2	NonS, T Miss, G Stop_gained COX1
	10605 (10603)	3	Spec, Synon, ND4
	12416 (12414)	2	NonS, Miss, ND5
	15961 (15959)	2	NonS, Up/down
$T_1b_1b_1$(24)	12404(12402)	9	NonS, Miss, ND5
	12740 (12738)	4	Spec, Miss, ND5
	14773 (14771)	5	Spec, Miss, ND5
$T_1b_1b_1a_3$ (9)	4742(4740)	3	NonS, Syno, ND2
$T_1b_1b_1c_1$(3)	13464 (13466)	2	Miss, ND5
T_1C (11)	15462 (15460)	2	Spec, Miss,CYTB
	9569 (9567)	5	NonS, Syno, COX3
T_2 (45)	16137(16135)	12	NonS, Up/down
	5703 (5701)	13	Spec, T Miss, A Stop gained
	11370 (11368)	9	Spec, Miss, ND4
T_3 (55)	16006 (16004)	59	Spec, Up/down
	8245 (8243)	32	Spec, Syno, ATP8
	6237 (6235)	5	Miss, COX1
	16076 (16074)	15	NonS, Up/down
	16233 (16231)		~Spec, Up/down

T_3c (5)	4002 (4000)	5	NonS, Miss, ND1
T_3k (5)	16114 (16112)	3	NonS, Up/down
T_3r_5	16044 (16042)	39	~ Spec, (T3a1a), Up/down
	16234 (16232)	4	NonS, Up/down
T_5	9234 (9232)	3	NonS, Miss, COX3
	10881 (10879)	3	NonS, Miss, ND4

*variants not used for determining haplogroups in MitoToolPy,
N = No. of animals in the haplogroup,
n = number of animals showing private variants,
Spec = Specific (i.e. Private variants from MitoToolPy only found within the group of individuals within a identified haplogroup),
NonS = Nonspecific (i.e. Private variants from MitoToolPy within a group of animals within a haplogroup but also present among other individuals in other haplogroups),
Variants: up/down upstream/downstream gene variants,
NCTE = Non-coding transcript exon, Miss Missense,
Syno = Synonynous

Table S6. Haplotypes specific to breed and prevalent in samples sourced from two countries.

**Shared haplotypes**	**No. of animals**	**Breed**	**Country**
Hap_12	3	Angus	Canada, USA
Hap_20	8	Angus	Canada, USA
Hap_121	2	Brown Swiss	Switzerland, Germany
Hap_145	3	Brown Swiss	Italy, USA
Hap_306	6	Holstein	Canada, Switzerland
Hap_308	3	Holstein	Canada, Switzerland
Hap_332	3	Holstein	Denmark, Germany
Hap_404	3	Holstein	France, The Netherlands
Hap_415	2	Holstein	France, The Netherlands
Hap_446	2	Jersey	Australia, USA
Hap_901	2	TyroleanGrey	Austria, Switzerland

Table S7. Number of animals in common (≥ 5) between each specific Haplotype (determined by DnaSP) and Cluster (based on hierarchical clustering of the nucleotide difference between pairs)

Haplotype No.	No. of Anim$^+$ in_hap	Cluster No.	No. of Anim$^+$ in Cluster	No. of Anim$^+$ common	% Concordance*
324	23	328	23	23	100%
7	21	7	21	21	100%
8	17	8	15	15	88%
197	15	198	14	14	93%
9	8	9	8	8	100%
855	8	866	8	8	100%
243	8	246	8	8	100%
20	8	20	8	8	100%
549	7	554	7	7	100%
24	7	24	7	7	100%
23	7	23	7	7	100%
19	7	19	7	7	100%
857	6	868	6	6	100%
825	6	835	6	6	100%
62	7	63	6	6	86%
505	6	510	6	6	100%
269	6	272	6	6	100%
1193	6	1209	6	6	100%
1192	6	1208	6	6	100%
1191	6	1207	6	6	100%
1080	6	1093	6	6	100%
786	5	795	5	5	100%
658	9	750	5	5	56%
544	5	549	5	5	100%
517	5	522	5	5	100%
515	5	521	5	5	100%
419	5	423	5	5	100%
306	6	310	5	5	83%
1138	5	1152	5	5	100%
10	5	10	5	5	100%
1029	5	1041	5	5	100%

Anim$^+$ = Animal, * based on No. of animals in each Haplotype

Table S8. Number and percentage of genotypes correctly imputed in 307 masked sites per animal in 333 animals (102231 sites per run) and correct prediction of haplogroup* from the imputed genotypes.

Run	Genotypes (out of 102231)		Haplogroups of 333 imputed animals	
	Correctly imputed	% Correctly imputed	No. correctly predicted	% of animal correct*
1	101951	99.73%	330	99.10%
2	101963	99.74%	333	100.00%
3	101987	99.76%	333	100.00%
4	101984	99.76%	333	100.00%
5	101921	99.70%	333	100.00%
6	101959	99.73%	332	99.70%
7	102036	99.81%	330	99.10%
8	101972	99.75%	333	100.00%
9	101938	99.71%	332	99.70%
10	101972	99.75%	332	99.70%
11	101983	99.76%	332	99.70%
12	101910	99.69%	333	100.00%
13	101953	99.73%	332	99.70%
14	102021	99.79%	330	99.10%
15	101963	99.74%	331	99.40%
16	101987	99.76%	332	99.70%
17	101976	99.75%	333	100.00%
18	101973	99.75%	332	99.70%
19	101991	99.77%	330	99.10%
20	101948	99.72%	328	98.50%
21	101999	99.77%	332	99.70%
22	101985	99.76%	332	99.70%
23	101964	99.74%	331	99.40%
24	101986	99.76%	332	99.70%
25	102005	99.78%	331	99.40%
26	101970	99.74%	331	99.40%
27	101978	99.75%	333	100.00%
28	101968	99.74%	333	100.00%
29	101925	99.70%	329	98.80%
30	102020	99.79%	333	100.00%
31	101993	99.77%	331	99.40%
32	101965	99.74%	331	99.40%
33	101985	99.76%	331	99.40%
34	101977	99.75%	331	99.40%
35	102000	99.77%	331	99.40%
36	101995	99.77%	331	99.40%
37	101957	99.73%	331	99.40%
38	101951	99.73%	331	99.40%

39	101995	99.77%	331	99.40%
40	101919	99.69%	331	99.40%
41	101990	99.76%	328	98.50%
42	102017	99.79%	328	98.50%
43	101973	99.75%	328	98.50%
44	101978	99.75%	328	98.50%
45	101938	99.71%	328	98.50%
46	101955	99.73%	333	100.00%
47	101960	99.73%	333	100.00%
48	102008	99.78%	333	100.00%
49	101985	99.76%	333	100.00%
50	102027	99.80%	332	99.70%
**Mean**	**101975**	**99.75%**	**331**	**99.49%**
